# Characterization of membrane structures regulating primary ciliogenesis by quantitative isotropic ultrastructure imaging

**DOI:** 10.1038/s41467-026-73638-4

**Published:** 2026-06-13

**Authors:** Quanlong Lu, Huijie Zhao, Ziam Khan, Adam Harned, Erina Kamiya, Valentin Magidson, Abhi Senthilkumar, Avaneesh Kilnagar, Phuong Thi Bich Doan, Sumeth Perera, Kedar Narayan, Christopher J. Westlake

**Affiliations:** 1 Laboratory of Cellular and Developmental Signaling, Center for Cancer Research, National Cancer Institute, National Institutes of Health, Frederick, MD USA; 2 Center for Molecular Microscopy, Center for Cancer Research, National Cancer Institute, National Institutes of Health, Frederick, MD USA; 3 Cancer Research Technology Program, Frederick National Laboratory for Cancer Research, Frederick, MD USA; 4 Optical Microscopy and Analysis Laboratory, Cancer Research Technology Program, Frederick National Laboratory for Cancer Research, Frederick, MD USA; 5 Department of Biochemistry, Faculty of Medicine, Sabaragamuwa University of Sri Lanka, Balangoda, Sri Lanka

**Keywords:** Cilia, Membrane curvature, Super-resolution microscopy

## Abstract

The trafficking, docking, and fusion of membrane vesicles at the mother centriole (MC) are important for primary cilium construction. Here, we determined the three-dimensional (3D) membrane ultrastructures, and associated proteins, involved in this primary cilium assembly mechanism upstream of axoneme growth. Our work suggests that the enlargement of small vesicles docked to the MC is a key trigger for ciliogenesis progression, a process requiring the MC distal appendage protein CEP164. These vesicles appear to fuse to form tubular C-shaped intermediates and an unprecedented toroidal membrane intermediate. The formation of these previously uncharacterized tubular membrane ciliogenesis intermediates is orchestrated by the membrane trafficking regulators EHD1 and RAB8, and is associated with the IFT-B complex protein IFT88. Remarkably, we show that EHD1, through its membrane tubulation function, regulates ciliogenesis progression by directly promoting CP110/CEP97 removal from the MC cap. The establishment of these tubular membrane structures is also associated with the recruitment of the ciliary gate transition zone proteins. Together, these findings redefine the architectural framework of early ciliogenesis and underscore the utility of isotropic ultrastructural imaging combined with quantitative 3D analysis for elucidating mechanisms of membrane trafficking and organelle biogenesis.

## Abstract (web summary)

This study describes a new mechanistic framework for the initiation of primary cilium formation, based on the characterization of unique vesicular membrane intermediates at the mother centriole revealed by three-dimensional ultrastructural microscopy.

## Introduction

Primary cilia extend from the surface of most eukaryotic cells, serving as sensory structures important for detecting and transducing mechanical and chemical signals which are essential for embryonic development, cellular polarity, and organogenesis^1–3^. Impairment in the formation and function of primary cilia causes human disease that can affect multiple organs and tissues. The primary cilium develops from the mother centriole (MC) and consists of a microtubule-based axoneme surrounded by a ciliary membrane^3^. The process of ciliogenesis is a tightly regulated sequence of events involving MC to basal body (BB) transition, transition zone (TZ) formation, and axoneme assembly^2,4^. In some cells, including cultured fibroblasts and human retinal pigment epithelial (RPE1) cells^5,6^, the initial stages of cilium biogenesis occur in the cytoplasm and requires the transport of preciliary vesicles (PCV) to the MC, a mechanism referred to as intracellular ciliogenesis^4,7–9^. In this pathway, membranes dock to the distal appendages (DA) on the MC and are referred to as DA vesicles (DAV). The DA proteins (DAP) are crucial for cilia formation and have been proposed to mediate the docking of DAVs to the MC by interacting with proteins on PCVs^10–12^. The hallmark of early ciliogenesis is a ~ 300 nm ciliary vesicle (CV)^4,13,14^ that covers the distal end of the MC that is assembled from smaller DAVs^7^. The intraflagellar transport (IFT)-B complex functions in axoneme formation at the CV stage^7^, and this vesicular structure reorganizes into a double membrane sheath around the extending axoneme. Initiation of axoneme growth requires the proteolytic removal of CP110 and CEP97, the MC cap, from the MC distal end via a process facilitated by DAPs^15–17^ and linked to the establishment of the CV^2,7,18^. TZ protein recruitment to the MC is also associated with the CV stage^7,19^ and leads to establishment of the TZ at the base of the cilium which serves to regulate ciliary transport.

Several membrane trafficking regulators have been identified as crucial for primary ciliogenesis, including members of the RAB small GTPase family^4,20–24^. RAB8 was the first RAB shown to function in ciliogenesis and was linked to assembly from the CV stage^7,25,26^. A RAB cascade involving RAB11 and RAB8 is integral to this process. RAB11 facilitates the vesicular transport of RABIN8, a RAB8 guanine nucleotide exchange factor (GEF), to the MC resulting in RAB8 activation^8,27^. Highlighting a complex poorly understood mechanism of membrane trafficking regulation of ciliogenesis RAB34 is also essential for CV formation^5,28–30^ and RAB19 has been linked to early stages of primary cilium assembly^31^. The RAB-associated membrane shaping and fusion proteins Eps15 homology domain-containing (EHD) proteins EHD1 and EHD3, and their associated factors PACSIN1/2, SNAP29 and MICAL-L1 are also important for pre-CV stages of ciliogenesis^7,19,32^. Additionally, EHD1 and PACSIN1 membrane tubulation function is important for the fusion of the CV and ciliary sheath membranes with the plasma membrane (PM) via the formation of extracellular membrane channels (EMC), thus exposing the ciliary-associated membrane to the extracellular region^19^. EHD1, PACSIN1 and MICAL-L1 are also essential for the removal of CP110/CEP97 from the MC cap^7,18,19,32,33^. How these trafficking regulators and associated membranes coordinate to initiate ciliogenesis on the MC through assembly of the CV is not clear.

Transmission electron microscopy (TEM) imaging has contributed greatly to our understanding of ciliogenesis pathways and has been used to identify all the known early ciliogenesis intermediate structures, such as DAV, CV, and ciliary sheath^2,4,13,15,34^. However, TEM is limited to two-dimensional (2D) imaging of ~70–80 nm thin sections, which complicates the interpretation of spatial relationships in three dimensions^35^. Furthermore, TEM resolution is insufficient to fully capture the MC, approximately 300 nm in diameter and 500 nm in height, plus associated ciliogenesis membrane structures. In contrast, volume electron microscopy (vEM) enables the reconstruction of 3D models from a series of 2D images^36^. The application of vEM in life sciences has significantly enhanced our understanding of the intricate details and spatial relationships of structures at the cellular level^37^. Among vEM techniques, focused ion beam scanning electron microscopy (FIB-SEM) enables precise sectioning at intervals of ~10 nm, providing isotropic resolution that minimizes distortion in 3D images^38^. This level of detail is particularly advantageous for visualizing cellular vesicles which can be as small as 30 nm in diameter^39^, as well as other structures generally below the resolution limits of most super-resolution light microscopy (SRM) techniques. Furthermore, FIB-SEM has been employed to describe membrane structures at the CV and sheath stages in ciliogenesis and in cilia resorption^19,40^. However, the characterization of MC docked pre-CV intermediates important in initiating ciliogenesis has not been defined by this approach.

In this study, we investigated the assembly of membranes on the MC during the early stages of ciliogenesis using FIB-SEM and SRM in RPE1 and human fibroblast cells. We quantitatively investigated membrane docking and organization at the MC DAs and identified previously uncharacterized intermediate processes of ciliogenesis, including DAV docking and expansion, the organization of asymmetric tubular membranes, and a toroidal membrane structure that we propose are upstream of CV formation and axoneme growth. By combining our vEM approach with protein depletion studies, we define ciliogenic roles for membrane trafficking regulators EHD1 and RAB8, and the DAP CEP164, and the IFT-B complex protein IFT88. We found that EHD1, in association with MC-associated tubular membranes, biochemically interacts with CP110 and CEP97 and directs their removal from the MC to enable ciliogenesis to progress. Additionally, we find tubular membrane assembly processes on the MC are linked to the recruitment of TZ proteins. This study demonstrates the application of isotropic vEM with quantitative structural analysis for understanding the molecular mechanisms of ciliogenesis.

## Results

### Identification of MC docked C-shaped membranes associated with ciliogenesis

**Fig. 1:**
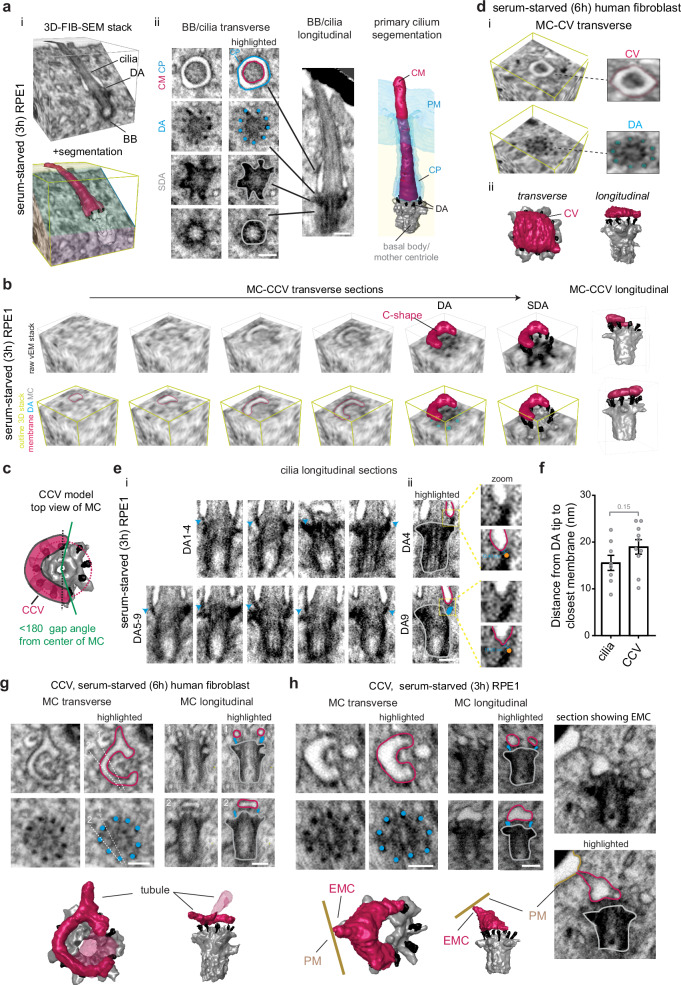
Identification of a CCV structure during ciliogenesis by vEM. **a** FIB-SEM image stack and segmentation of primary cilium structure from a RPE1 GFP-CETN1 cell serum-starved (3 h). (i) 3D FIB-SEM image stack. Segmented BB, DA and cilium (bottom image) from the volume integrated into the raw FIB-SEM image stack (top image). *xyz* planar sections are colored differently in the bottom image. (ii) Longitudinal and transverse sections showing CM: ciliary membrane (magenta), PM: plasma membrane, CP: ciliary pocket (outlined in cyan in vEM image). DAs are highlighted with cyan colored dots, and the MC/basal body is traced in grey in vEM images. PM and CP are shown in cyan in segmented image. Representative image from three independent experiments. Scale bars (white line): 200 nm. **b** MC transverse FIB-SEM 3D image stack from a serum-starved (3 h) RPE1 GFP-CETN1 cell with a C-shaped membrane above the DAs of the MC. 3D vEM planes show (top and bottom images are identical positions) above the MC and at the MC DA ends and at SDAs. To visualize MC associated structures unobstructed, membranes and MC structures are highligted. Right images show 3D segmented structures for two different longitudinal views of the MC and C-shaped membrane. **c** Model showing C-shaped MC docked membranes covering 50% or greater of the DA circumference are classified as CCVs. **d** (i) MC transverse FIB-SEM 3D image stack containing a CV above the MC DA from a human fibroblast cell expressing GFP-CETN1 serum-starved for 6 h. (ii) Longitudinal and transverse 3D segmentation of MC and CV from (i). **e** Longitudinal sections showing DA-ciliary membrane interactions from the serum-starved RPE1 cell described in **a**. (i) Image sections showing all nine individual DAs, identified by a blue arrowhead. (ii) Highlighted DAs (cyan), BB (grey) ciliary membrane (magenta), and DA proximal tip (orange dot). Magnified images (zoom) show representative 3D measurements for the closest position between the membrane and the proximal end of the DA. The distances measured are 12 nm and 18 nm in the top and bottom panels, respectively. **f** Plot showing distance from DA ends to membrane in cilia and CCV structures in RPE1 (GFP-CETN1 and GFP-CETN1 + SMO-tRFP; 3 or 6 h serum-starved) and human fibroblast (GFP-CETN1; 6 h serum-starved) cells. Cells were identified from three independent experiments (8 cells with cilia; 10 cells with CCV). Mean ± SD, two-tailed *t*-test. **g, h** 3D structural analyses of the CCV at the distal end of the MC in RPE1 GFP-CETN1 (**h**) and human fibroblast GFP-CETN1 (**g**) serum-starved (**g**: 6 h; **h**: 3 h) cells. Individual DA-membrane associations are shown as described in (**e**). White dotted lines in (**g**) correspond to longitudinal sections of the centriole shown. Membrane tubules extending from the CCV (**g**) and an EMC connection to the PM (**h**) are shown. Representative images from two (**g**) and three (**h**) independent experiments. Scale bars: 200 nm. Yellow lines are used in the vEM image stacks to highlight the 3D position of the section of the MC-associated structures shown (**a,** **b,** **d**).

The MC DAs and associated membranes of the primary cilium can be visualized by FIB-SEM (Fig. 1a, Supplementary Movie 1). To investigate the 3D organization of the MC and associated membranes during ciliogenesis by FIB-SEM, we employed a correlative light and electron microscopy (CLEM) approach with cells expressing the centriole/basal body (BB) marker GFP-Centrin 1 (GFP-CETN1) to precisely target regions of the cell for imaging. To enrich for cells at pre-axonemal ciliogenesis stages, we expressed a tagRFP (tRFP)-fused SMO, a ciliary transmembrane receptor involved in the Hedgehog pathway, to facilitate monitoring of cilia formation^7,19^. Serum starvation promotes asynchronous ciliation in RPE1 cells, with early ciliogenesis structures such as DAVs and CVs expected after 3 or 6 h serum removal^7^. FIB-SEM imaging was performed on cells subjected to 3 and 6 h of serum-starvation, generating high resolution aligned image stacks for five and six cells, respectively, under these treatment conditions. Consistent with starvation-dependent ciliogenesis initiation, two of the six cells serum-starved for 6 h exhibited short cilia (1.3 and 1.1 μm) and one cell displayed a CV, while these ciliogenic structures were not observed in the five cells serum-starved for 3 h or in four serum-fed control cells (Supplementary Table 1). Interestingly, a C-shaped tubulovesicular structure was present near the MC in a single cell in each of the serum-starved conditions (Supplementary Table 1), but not in the serum-fed RPE1 cells. To rule out that the formation of these C-shaped MC-associated membrane structures was associated with SMO-tRFP expression, we performed similar studies in RPE1 GFP-CETN1 cells. Strikingly, six out of sixteen (37.5%) randomly selected 3 h serum-starved cells imaged by FIB-SEM had C-shaped membranes near the DAs of the MC (Fig. 1b, Supplementary Table 1, Supplementary Movie 2), whereas only one of the nine (11%) serum-fed RPE1 GFP-CETN1 cells displayed a C-shaped membrane. C-shaped membranes were defined as covering >50% of the circumference of the DA region but not fully encircling the MC (Fig. 1c). Likewise, FIB-SEM studies in nine randomly selected serum-starved (6 h) primary human fibroblasts expressing GFP-CETN1 showed two C-shaped membranes (Supplementary Table 1), one CV (Fig. 1d, Supplementary Fig. 1a and Supplementary Movie 3), and three cilia (Supplementary Table 1). Together these findings suggest that the C-shaped membrane is a previously uncharacterized intermediate structure in ciliogenesis, that forms upstream of the CV stage.

To further evaluate if the C-shaped membrane is associated with ciliogenesis on the MC, we determined the minimal distances between these membranes and the proximal ends of the nearest DAs and compared these 3D spatial determinations to similar measurements performed on cilia (Fig. 1e and Supplementary Movie 4). The average minimal distance from the C-shaped membrane to the closest DA was found to be 19 ± 2 nm, which was not significantly different from that observed in cilia (Fig. 1f). These results support the conclusion that the C-shaped membrane is docked to the MC and is a ciliogenesis intermediate, which we refer to as the “C”-ciliary vesicle (CCV). The structure of the CCV suggests this membrane possesses tubulogenic membrane properties. This is further supported by observations that 5 out of 11 reporter cells (RPE1 and human fibroblast) with CCVs extend membrane tubules away from the MC (Fig. 1g, h, Supplementary Fig. 1b). Of particular interest, three of these CCV containing RPE1 cells display tubular EMCs connecting the CCV to the PM (Fig. 1h and Supplementary Fig. 1b). The identification of CCV-EMCs suggests that membrane connections to the PM occur prior to the CV stage of ciliogenesis. The more prominent detection of CCV compared to CV in serum-starved RPE1 and human fibroblasts expressing GFP-CETN1 (Supplementary Table 1) suggests the CCV is a longer-lived intermediate than the CV, which is surprising given CVs are a hallmark structure of the intracellular ciliogenesis pathway from previous TEM studies^7,13^. However, 3D structural analysis suggests that the CCV can be misidentified as CV and DAV stages of ciliogenesis depending on the specific longitudinal EM section of the MC being examined (Fig. 1g, h and Supplementary Fig. 1b), which is typical of TEM studies. Likewise, the ciliary sheath/cilia could be misidentified as a CV depending on the sectioning plane, as can be seen in FIB-SEM sections shown in Fig.1e (i, image for DA3). These observations underscore the importance of employing vEM to elucidate the organization of MC-associated membranes during ciliogenesis. Together, these findings reveal a previously unrecognized role for tubular membranes in cilium assembly.

### Characterization of DAV docking and organization in ciliogenesis progression

**Fig. 2:**
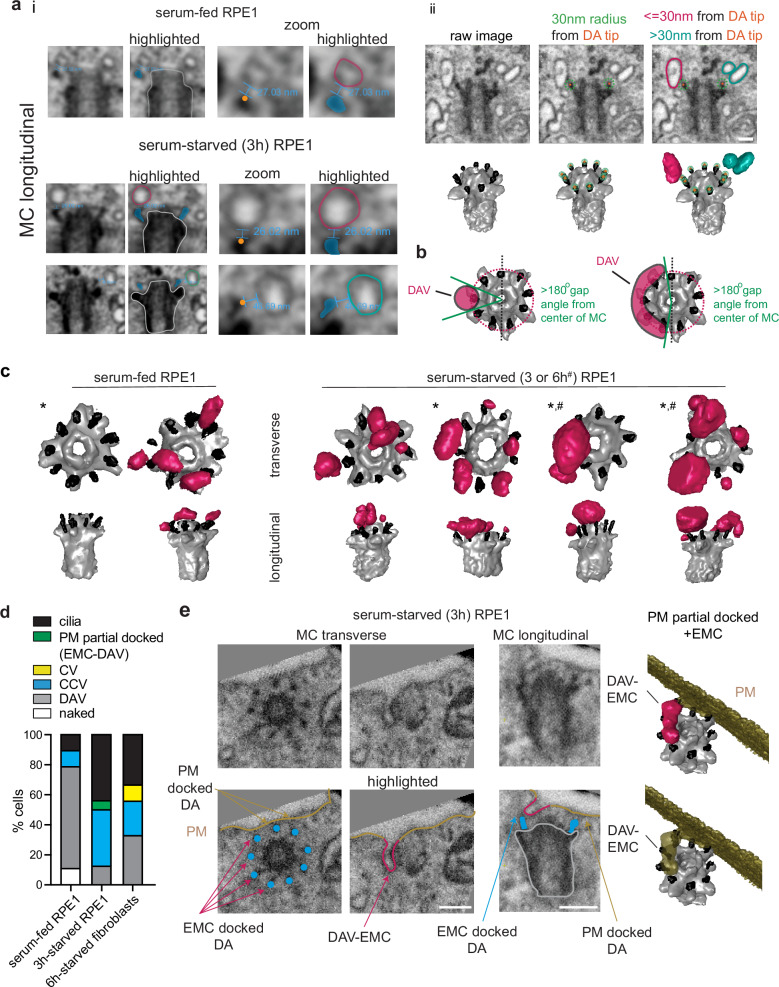
Characterization of DAV docking under ciliating and non-ciliating conditions. **a** (i) FIB-SEM images of RPE1 GFP-CETN1 cells serum-fed or serum-starved (3 h) showing membrane vesicle distances to the proximal DA ends (orange dots). Measurements shown are 27 nm in serum-fed cell, 26 nm in top panel of starved cell and 47 nm in bottom panel of starved cell. (ii) FIB-SEM and segmented images show a 30 nm spherical radius (green dotted line or mesh sphere) at the ends of the DA (orange dot). DAVs within 30 nm of the DA ends (magenta) and undocked vesicles more than 30 nm from the DA ends (teal) are shown. Representative image from two independent experiments. Scale bar: 200 nm. **b** Model showing DAV covering less than 50% (>180° gap from center of MC) of circumference of the DA region. **c** Representative FIB-SEM segmentation of MCs from RPE1 GFP-CETN1 and RPE1 GFP-CETN1 + SMO-tRFP (*) cells grown in serum or serum-starved for 3 or 6 h(#) showing the absence (naked) and presence of DAV docked within 30 nm of the end of the DAs. Segmentations for all DAV containing cells for these conditions are shown in Supplementary Table 1. **d** Quantification of structures observed at the MCs in RPE1 GFP-CETN1 (serum-fed = 9 cells; serum-starved 3 h = 16 cells) and human fibroblasts GFP-CETN1 (serum-starved 6 h = 9 cells) shown in Supplementary Table 1. RPE1 cells imaged from two or more experiments, human fibroblasts from one experiment. PM-partial docked corresponds to MC where some DAs are docked to the PM without an axoneme protrusion into the membrane as shown in (**e**). **e** vEM identification of partial MC docking to the PM via an EMC not associated with a CCV or later ciliogenesis stage. RPE1 GFP-CETN1 cells were serum starved for 3 h as described in (**c**). FIB-SEM images (*left panels*) and segmented images (*right panels*) suggest short EMC structures are associated with a DAV stage; referred to as DAV-EMC. Another example of an RPE1 reporter cell with a MC showing a DAV-EMC structure is shown in Supplementary Table 1. Scale bars: 200 nm. Representative image from three independent experiments.

The discovery of CCVs and their potential misidentification as DAVs in 2D ultrastructure images raises questions about the organization and assembly of membranes on the DAs at earlier stages in ciliogenesis. To investigate if DAVs are associated with CCV assembly, we examined whether smaller membrane structures were present on the MC during ciliogenesis in all the FIB-SEM imaged RPE1 and human fibroblast reporter cells described. The lower size limit of cellular vesicles is 30 nm in diameter, which can be resolved by FIB-SEM. Vesicles >30 nm were identified in FIB-SEM datasets after measuring their diameters and observing a continuous structure across two consecutive vEM sections. Membranes were considered docked to the MC if they were within 30 nm of the DA-ends (Fig. 2a, Supplementary Fig. 2 and Supplementary Movie 5), which corresponds to the average CCV-DA docking distance determined (Fig. 1f) plus an additional 10 nm to account for the resolution limit of FIB-SEM. MC docked spherical or tubular vesicular membranes were defined as DAVs if a single structure covered less than 50% of the circumference of the DA region (Fig. 2b). Analysis of the nine serum-starved (3 and 6 h) RPE1 cells (GFP-CETN1 and GFP-CETN1 + SMO-tRFP) lacking cilia, CV, and CCV showed that six cells had DAVs, while the other three cells displayed full or partial MC docking to the PM (Fig. 2c, d, Supplementary Table 1). Similarly, DAVs were observed at the MC in the serum-starved human fibroblasts lacking these larger ciliary membranes (Fig. 2d and Supplementary Table 1). Interestingly, only a single RPE1 GFP-CETN1 cell and RPE1 GFP-CETN1 + SMO-tRFP cell (Fig. 2d, Supplementary Table 1) tested under serum-fed conditions did not have DAVs, referred to as naked, while nine out of these thirteen combined reporter cells displayed DAVs (Fig. 2c, d and Supplementary Table 1). These results support a model whereby increased DAV docking and membrane reorganization at the MC are associated with ciliogenesis progression. Interestingly, a fully PM docked MC was observed in a 3 h serum-starved RPE1 reporter cell suggestive of the extracellular ciliogenesis pathway, whereby DAs can directly dock to the PM prior to axoneme growth^6^. However, two similarly serum-starved RPE1 reporter cells showed MCs partially docked to the PM through EMC-like structures, with some DAs more than 30 nm away from the PM (Fig. 2d, e, and Supplementary Movie 6). These partially PM docked MCs with EMC-like structures support requirements for vesicular docking to DAs consistent with the intracellular ciliogenesis pathway. Importantly, in these cases, 2D EM images could lead to an incorrect interpretation that either the MC is not associated with the PM/ciliary membrane or that it is fully docked to the PM (Fig. 2e). Overall, our results indicate that both non-ciliating and ciliating cells typically contain DAVs within 30 nm of the MC DA ends, which we hypothesize are precursor membranes for the CCVs and are also capable of fusion with the PM.

**Fig. 3:**
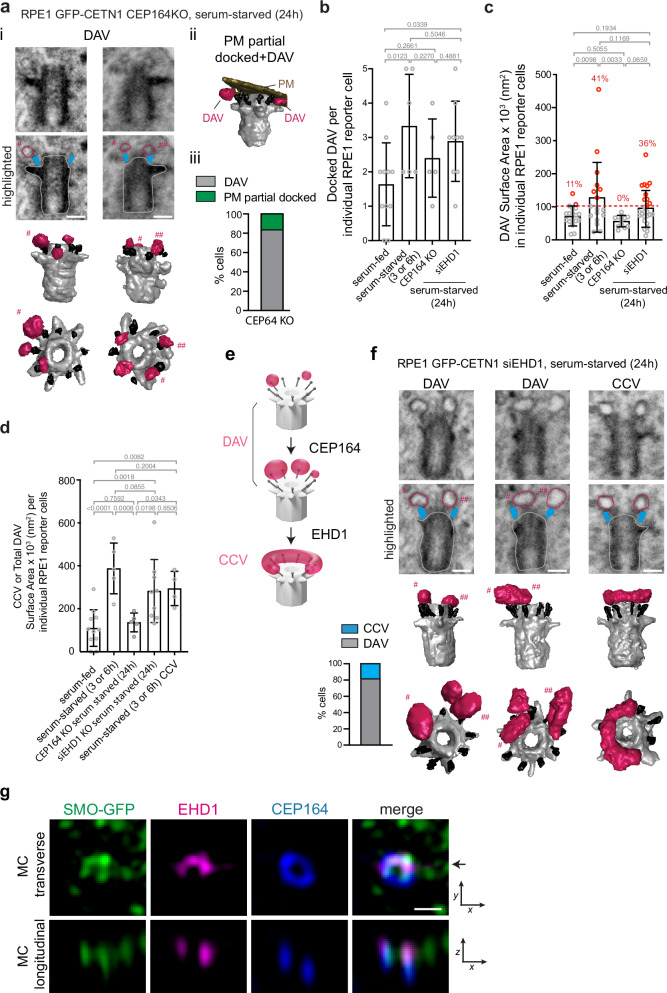
CEP164 and EHD1 function in ciliogenesis after DAV docking to the MC. **a** CEP164 knockout blocks ciliogenesis at the DAV stage. Representative segmented MCs (*top panel*) from RPE1 GFP-CETN1 CEP164 CRISPR KO cells (6 cells, shown in Supplementary Table 1) serum starved for 24 h. (i) shows DAV stage, (ii) shows a MC partially docked to the PM containing DAVs. (iii) shows quantification of MC structures detected in these cells. # and ## denote specific DAVs shown. Scale bar: 200 nm. **b** Quantification of the total number of DAVs docked to individual MCs in RPE1 (GFP-CETN1 and GFP-CETN1 + SMO-tRFP) cells treated with serum (11 cells), serum-starved for 3 or 6 h (6 cells), lacking CEP164 serum-starved for 24 h (5 cells), and treated with siEHD1 (9 cells, described in **f**) in cells found to only have DAVs and lacking EMCs. All described MCs are shown in Supplementary Table 1. Mean ± SD, One-way ANOVA. **c** Plot showing the individual DAVs docked to MCs in RPE1 reporter cells described in (**b**). Data points in red show DAVs with surface area 1 SD greater than the average surface area for serum treated cells (denoted by red dashed line). Mean ± SD. One-way ANOVA. **d** Plot showing the total surface area of DAVs and CCVs (6 cells, without tubule extensions and EMC described in Supplementary Fig. 2b) docked to each MC from RPE1 reporter cells treated as described in (**b**). Mean ± SD, One-way ANOVA. **e** Model showing ciliogenesis requirements for CEP164 and EHD1 in DAV expansion and CCV formation, respectively. **f** EHD1 depletion blocks ciliogenesis upstream of the CCV stage. Representative segmented MCs (*top panel*) from EHD1 GFP-CETN1 siRNA treated (72 h) cells (11 cells, shown in Supplementary Table 1) serum-starved for the last 24 h. Plot (*bottom*) shows quantification of pre-CV MC structures detected in these cells. # and ## denote specific DAVs shown. **g** SRM SIM image showing endogenous EHD1 localization to asymmetric membrane structures along with SMO-GFP in RPE1 cells serum-starved for 6 h. DAs are labeled with a CEP164 antibody. MC transverse view shows a projection of image slices along the Z-axis, and the MC longitudinal view shows a projection of image slices along X-axis. Directional arrows indicate imaging plane. Samples were imaged with Nikon NSIM. Scale bar: 500 nm. Representative image from 3 independent experiments.

To investigate the MC-DAV docking mechanism, we examined the effects of ablation of the DAP CEP164, which has been reported to prevent membrane docking to the MC in TEM studies^41,42^. CEP164 was knocked out of RPE1 cells using CRISPR-Cas9 (Supplementary Fig. 3a, b) and we confirmed this protein’s absence prevented cilia development, which was restored by expressing GFP-CEP164 (Supplementary Fig. 3a, c, d). Nine DAs were observed in the CEP164 KO cells based on FIB-SEM analysis (Fig. 3a, Supplementary Table 1). Remarkably, FIB-SEM analysis of CEP164 KO cells identified DAVs within 30 nm of the DAs at the MC in all cells that were serum-starved for 24 h. These findings suggest that CEP164 is dispensable for MC docking to ciliary associated membranes. Furthermore, a partially PM-docked MC with two DAVs was observed in one of the cells imaged (Fig. 3a and Supplementary Fig. 3e), which supports a model wherein fusion with PM can occur at the DAV stage.

To examine the ciliogenic potential of DAVs, we evaluated the distribution and organization of these membranes in the RPE1 reporter cell lines imaged by FIB-SEM grown under non-ciliating (serum-fed) and ciliating conditions (serum-starved). Our analysis indicates that serum starvation promotes increased DAV docking to the MC compared to serum-fed cells (Fig. 3b). Quantification showed that the average surface area of individual DAVs observed in cells under ciliating conditions (128 ×10^3^ ± 105×10^3 ^nm²) was significantly larger than in serum-fed cells (71×10^3^ ± 31 ×10^3 ^nm²) (Fig. 3c). These results suggest that ciliogenesis progression is associated with bigger DAVs, which could result either from the docking of larger vesicles to the MC or from DAV fusion with smaller vesicles transported to the MC. The latter DAV expansion model is supported by observations that smaller DAVs coexist on the same MC with DAVs that are 3 to 4 times larger (Fig. 2c and Supplementary Table 1). Examination of the size of the DAVs in serum-starved CEP164 KO cells showed these membranes were typically less than 60×10^3 ^nm², significantly smaller in size than found in serum-starved (3-6 h) wild-type RPE1 reporter cells (Fig. 3c). These results suggest that CEP164 is required to organize larger DAVs on the MC during ciliogenesis. Notably, there is sufficient membrane in the DAVs of serum-starved (3 h and 6 h) wild-type RPE1 fluorescent reporter cells to form a CCV based on comparison of the surface areas of these membranes (Fig. 3d), but not in starved CEP164 KO RPE1 cells. Together these findings support a ciliogenesis progression model wherein larger DAVs are organized on the MC, a process requiring CEP164, followed by their fusion to form a CCV membrane structure (Fig. 3e).

### EHD1 functions in fusion of enlarged DAVs into the CCV

Because EHD1 function has been linked to DAVs at pre-CV stages by TEM-based analysis^7^ and membrane tubule formation and vesicle fusion^43–45^, we further investigated its ciliogenesis function by FIB-SEM in RPE1 GFP-CETN1 cells treated with siRNA that depletes >90% of EHD1 protein^7^. Ciliogenesis disruption at pre-CV stages was observed in EHD1-knockdown serum-starved (24 h) cells, with nine out of these eleven cells displaying DAVs and two cells having CCVs (Fig. 3f, Supplementary Table 1). The predominant arrest at the DAV stage suggests a requirement for EHD1 upstream of CCV assembly. Consistent with this EHD1 function, RPE1 GFP-CETN1 EHD1-knockdown cells did not differ significantly in the proportion of cells with DAVs relative to CCVs compared with RPE1 GFP-CETN1 CEP164 KO cells starved for 24 h (*p* = 1). In contrast, a significant difference in the proportion of these structures (*p* = 0.024) was observed between EHD1-knockdown cells and WT RPE1 GFP-CETN1 cells starved for 3 h (Supplementary Table 1). One explanation for the presence of CCVs in a minority of EHD1-knockdown cells is that residual EHD1 protein levels remaining after siRNA treatment are sufficient to drive CCV membrane assembly. EHD1 functioning at the CCV stage is further supported by structured illumination microscopy (SIM) observations of EHD1 on CCV-like structures that partially colocalized with asymmetrically accumulated SMO-GFP (Fig. 3g). EHD1-knockdown serum-starved cells exhibited significantly greater numbers of DAVs and total DAV surface area compared to untreated RPE1 cells grown under serum-fed non-ciliating conditions (Fig. 3b, d), while the number and average size of these MC membranes in the EHD1 knockdown cells were comparable to serum-starved (3 and 6 h) untreated RPE1 cells (Fig. 3b, c). EHD1 function in CCV assembly on enlarged DAVs is further supported by the observation that the total amount of DAV membranes in serum starved (24 h) EHD1-knockdown cells were significantly higher than in similarly treated CEP164 KO cells (Fig. 3d). Moreover, sufficient DAV membranes are present on the MC of these EHD1-knockdown cells to account for the size of an average CCV (Fig. 3d), yet these membranes largely failed to organize into the CCV. Collectively, these results support a model in which EHD1 facilitates the organization of DAVs into the CCV during ciliogenesis (Fig. 3e).

### Assembly of a toroidal intermediate from the CCV and DAVs

**Fig. 4:**
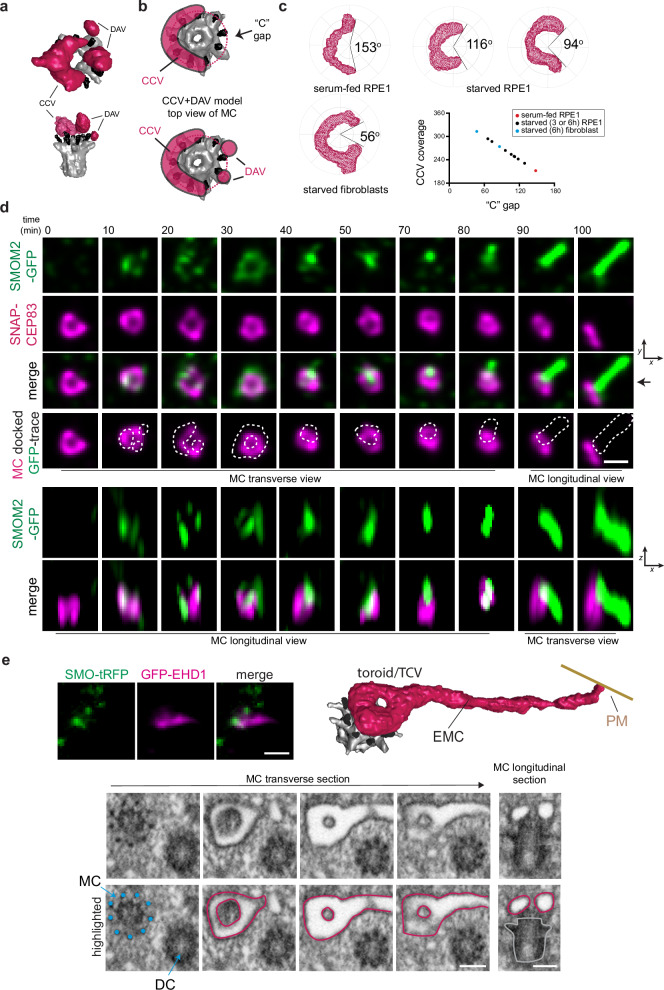
Ciliogenesis progression and a membrane toroid TCV stage. **a** Representative image of DAV docking in the CCV “C”-shaped gap. FIB-SEM segmentation from a serum-starved RPE1 GFP-CETN1 cell shown in Supplementary Table 1. **b** Model showing DAVs in the CCV “C”-shaped gap on the MC. **c** “C”-shaped gap angle determination from the center of the MC to the two ends of tubular CCV from RPE1 (9 cells) and human fibroblast (2 cells) reporter cells shown in Supplementary Fig. 2b. Plot shows distribution of CCV coverage and “C”-shaped gaps for each cell. ° = degree “C”-gap measured. **d** SRM SIM^2^ live cell time-lapse imaging of a RPE1 cell showing ciliogenesis progression. Cells stably expressed SMOM2-GFP to monitor vesicle docking and ciliary membrane assembly at the MC marked by SNAP-CEP83 (labelled with SNAP-Cell647-SiR). Cells were imaged every 10 min following serum starvation. Images show pre- and post- SMOM2-GFP detection on MC DAs more than 10 min after serum withdrawal. Note that the MC has rotated to a side view in the imaging plane at the 70, 80, and 90 min time points. Panels with dotted lines show the outline of SMOM2-GFP structures associated with the DA. MC transverse and longitudinal images correspond to the planar position of the MC. Top image panels show projections of image slices associated with the MC in the Z-axis and the bottom image panels show the orthogonal view along the X-axis corresponding to the position of the black arrow. Directional arrows indicate imaging plane. Scale bar: 500 nm. **e** CLEM identification of a TCV structure in an RPE1 cell expressing GFP-EHD1 and SMO-tRFP serum-starved for 6 h. Cells were imaged by SD confocal (*top left panels*). FIB-SEM images (bottom panels) showing transverse and longitudinal sections of the MC through the toroid membrane with and without traced DAs (cyan), TCV associated membrane (magenta) and the MC (grey). Additional FIB-SEM images of this cell are shown in Supplementary Fig. 5. Segmented image of the TCV with an EMC (top right panel). Six total cells were imaged and analyzed showing 1 TCV, 4 CV or short cilia, and 1 naked MC. DC: daughter centriole. Scale bar for confocal: 2 µm. Scale bars for vEM: 200 nm.

The identification of the CCV as a prominent intermediate structure in ciliogenesis prompted further investigation into how these membranes could be organized into a CV. FIB-SEM analysis revealed that ~40% of all cells observed with CCVs had associated DAVs in the gap between the ends of the CCV (Fig. 4a, Supplementary Fig. 2b, Supplementary Movie 7 and Supplementary Table 1), that we refer to as the “C” gap (Fig. 4b). This finding raised the possibility that these DAVs fuse with the CCV to create larger structures with a reduced “C” gap. Indeed, a CCV expansion process is supported by comparison of the “C” gap in different cells (Fig. 4c). Specifically, the lone CCV observed in serum-fed RPE1 cells showed the largest “C” gap at 153°, while the membrane gap in serum-starved cells was smaller ranging from 129° to 47° (average 93 ± 24°). Thus, progressive fusion of the CCV with DAVs in the “C” gap could form a continuous membrane around the MC upstream of CV organization.

To further investigate this ciliogenesis membrane assembly progression mechanism in live cells, we established an RPE1 cell line expressing the M2 variant of SMO (SMOM2-GFP), which shows less cytoplasmic signal around the MC than SMO-GFP, and the DAP CEP83 fused to a SNAP-tag. Using SRM-based SIM^2^ live-cell time-lapse imaging, we could monitor membrane organization on the MC during ciliogenesis (Fig. 4d, Supplementary Fig. 4 and Supplementary Movie 8). When the DAs faced upward or downward in the plane of imaging fluorescently labelled SNAP-CEP83 appeared as a ring structure. Importantly, SMOM2-GFP was detected accumulating as small punctate structures on one side of the DA-ring that progressed into CCV-like structures before consolidating into a more central structure at the end of the MC. SMOM2-GFP localization shifted from overlapping with CEP83 to centering within the CEP83 ring; this shift may coincide with axoneme growth, given SMOM2-GFP stronger accumulation in the ciliary membrane than in the preceding vesicular ciliogenesis structures^7^. Remarkably, prior to this ciliogenic state the DA associated SMOM2-GFP shifts from an asymmetric DAV or CCV-like appearance to a ring-like structure within a 10-min interval (Fig. 4d, Supplementary Fig. 4 and Supplementary Movie 8). These structures are consistent with either DAVs or CCV, but they may also suggest the presence of a toroidal membrane, resulting from DAV docking and fusion with the CCV in the “C” gap.

To investigate whether a toroidal intermediate is associated with ciliogenesis, we performed CLEM FIB-SEM on RPE1 cells expressing GFP-EHD1 and SMO-tRFP. These fluorescent reporters were used to screen for cells with potential tubular membrane intermediates at pre-axonemal growth stages on the MC. Strikingly, in one cell, we identified a membrane toroid docked to the DAs of the MC, which we designated the toroidal ciliary vesicle (TCV) (Fig. 4e, Supplementary Fig. 5 and Supplementary Movie 9). This TCV structure also displayed an EMC, consistent with PM connections being established at pre-CV membrane ciliogenesis stages. Together these observations support a ciliogenesis mechanism where the CCV can organize into a TCV upstream of the CV.

### Ciliogenesis tubular membrane organization directs MC uncapping and is associated with transition zone protein recruitment

**Fig. 5:**
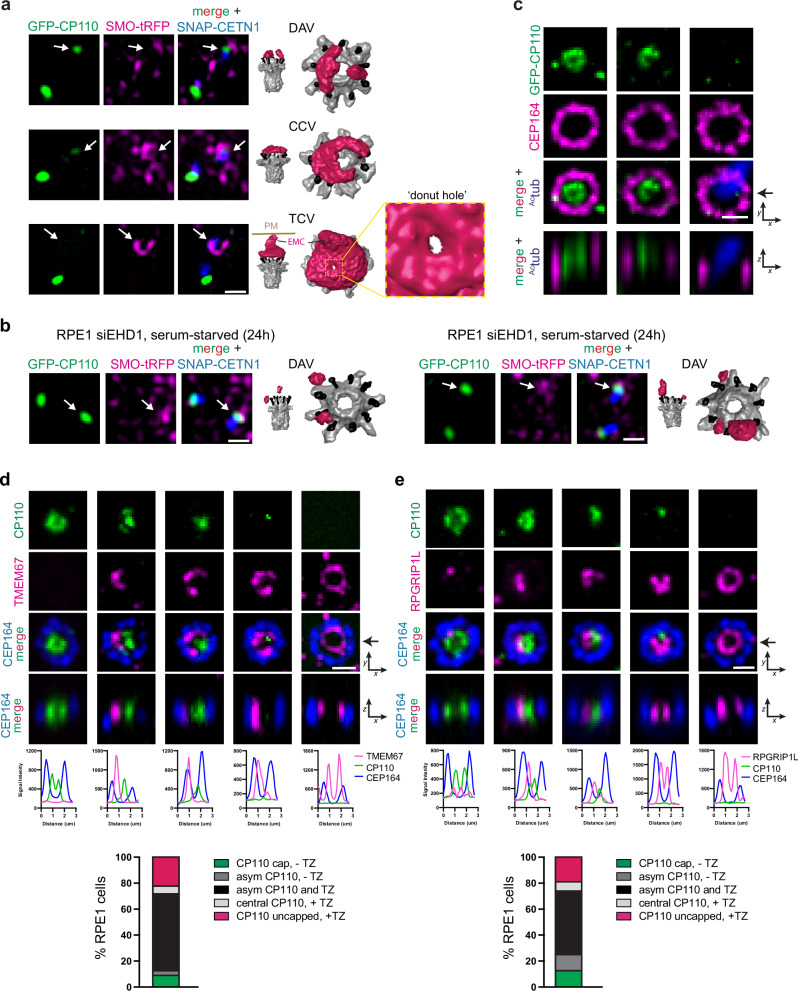
Asymmetric CP110 uncapping is associated with tubular membrane assembly and TZ protein recruitment on the MC. **a** DAV, CCV and TCV stages are associated with CP110 uncapping of the MC. RPE1 cells stably expressing GFP-CP110/SMO-tRFP/SNAP-CETN1 were serum starved for 6 h. Cells were fixed and imaged by SIM (Nikon) (*left panels*) followed by CLEM FIB-SEM (6 cells; 3 representative cells shown) and segmentation (*right panels*). Zoomed region (*bottom panels*) shows ‘donut-hole’ region—see additional vEM images in Supplementary Fig. 7. SIM images of the bottom panel appear to show the side view of the TCV and associated EMC. White arrows show MC position. Scale bar: 500 nm. **b** CLEM FIB-SEM analysis of CP110 capped MC and DC in EHD1-knockdown cells. RPE1 cells described in (**a**) were treated with EHD1 siRNA for 72 h with serum starvation for the last 24 h. Cells were fixed and imaged by SIM (Nikon, *left panels*) followed by CLEM FIB-SEM and segmentation (*right panels*). The number of DAV per cell (2.75 ± 0.95) and the average surface area of the DAVs (71 × 10^3^ ± 53 × 10^3 ^nm²) from the four cells imaged (shown in Supplementary Table 1) was not significantly different than RPE1 CETN1 cells treated with siEHD1 described in Fig. 3c (DAV per cell *p *= 0.83, DAV surface area *p* = 0.27). Scale bar: 1 μm. **c** SRM STED imaging demonstrates that CP110 removal from the MC can occur asymmetrically. RPE1 cells were serum starved for 6 h and stained with CP110, CEP164, and ^Ac^tub antibodies and imaged by STED microscopy for the centriole markers and epifluorescence imaging for the cilia marker. Top images show a projection of images across the MC distal end region. Bottom panels show the orthogonal slice along the Z-axis corresponding to the position of the black arrow. Scale bar: 500 nm. Representative image from 3 independent experiments. **d** pro-ExM SRM imaging showing asymmetric CP110 removal from the MC distal end in relation to the TZ protein TMEM67. Representative RPE1 cells serum starved for 6 h from three independent experiments showing progression of MC uncapping. Directional arrows indicate imaging plane. Top images show a projection of images across the MC distal end region. Bottom images show the orthogonal view along the Z-axis corresponding to the direction of the black arrow. Fluorescence intensity plot profiles correspond to the position of the black arrow. Bottom plot shows the CP110 and TMEM67 localizations observed in the 41 cells imaged (Green: MC fully capped with CP110, no TZ; Dark Grey: asymmetrically localized CP110, no TZ; Black: asymmetrically localized CP110 and TZ; Light Grey: asymmetrical TZ, CP110 localizes at the center of DA; Red: TZ with CP110 fully removed). Additional images showing reported localizations in the plot are included in Supplementary Fig. 8f. All three experiments showed CP110 levels at the MC exhibited a negative relationship with MC TMEM67 by linear regression analysis (Experiment 1: *R*^2^ = 0.27, slope = −2.45 ± 0.87, *p* = 0.010, slope error = 0.87, cells = 24; Experiment 2: *R*^2^ = 0.41, −5.91 ± 2.36, *p* = 0.033, slope error = 2.4, cells = 11; Experiment 3: *R*^2^ = 0.66, −3.77 ± 1.34, *p* = 0.049, slope error = 1.4, cells = 6). Pairwise comparisons of regression slopes revealed no significant differences among experiments (all *p* ≥ 0.19). Scale bar 1 μm. **e** TZ protein RPGRIP1L accumulates at the MC where CP110 has been removed. RPE1 cells were processed for pro-ExM and stained with RPGRIP1L, CP110 and CEP164 antibodies and imaged and displayed as described in (**d**). Fluorescence intensity plot profiles correspond to the direction of the black arrow. Plots show CP110 and RPGRIP1L localizations observed as described in (**d**) from three independent experiments for 35 cells imaged. Additional images showing reported localizations in the plot are included in Supplementary Fig. 8f. All three experiments showed CP110 levels at the MC exhibited a negative relationship with MC RPGRIP1L by linear regression analysis (Experiment 1: *R*^2^ = 0.14, slope = −3.99, *p* = 0.2465, slope error = 3.226, cells = 11; Experiment 2: *R*^2^ = 0.19, slope = −6.281, *p* = 0.0522, slope error = 3.024, cells = 20; Experiment 3: *R*^2^ = 0.87, slope = −2.83, *p* = 0.0858, slope error = 1.027, cells = 4). Pairwise comparisons of regression slopes revealed no significant differences among experiments (all *p* ≥ 0.29). Scale bar = 1 μm.

We next investigated the association between ciliary membrane assembly intermediates and other ciliogenesis processes occurring at the MC, specifically MC uncapping and TZ protein recruitment. We show quantitatively that SMO-GFP accumulation on the MC is correlated with the progressive removal of CP110 from the MC (Supplementary Fig. 8a). Moreover, SIM SRM imaging in RPE1 and human fibroblast cells expressing SMO-GFP suggests that CP110 removal advances from DAV to CCV or TCV ciliary membrane structure establishment on the MC (Supplementary Fig. 6a, b). To further explore the relationship between ciliary membrane structure and MC uncapping, we conducted CLEM FIB-SEM imaging on serum-starved RPE1 cells expressing GFP-CP110, SMO-tRFP and SNAP-CETN1. To enrich for early ciliogenesis membrane structures, cells were selected for FIB-SEM imaging that lacked elongated SMO-tRFP positive cilia and showed partial or complete GFP-CP110 loss from one of the SNAP-CETN1 positive centrioles by SIM (Fig. 5a). These CLEM studies further support a mechanism whereby the removal of GFP-CP110 from the MC is associated with progression from DAVs to the TCV stage, at which point CP110 is no longer present at the MC (Fig. 5a and Supplementary Fig. 7). Additionally, the TCV structure observed in Fig. 5a had only a small donut hole, suggestive of it being a stage just upstream of CV establishment (Supplementary Fig. 7). Consistent with this proposed MC uncapping model, CLEM FIB-SEM imaging of EHD1-knockdown RPE1 GFP-CP110 SMO-tRFP SNAP-CETN1 cells showed that cells retaining GFP-CP110 at the MC had DAVs similar in size to those in EHD1-knockdown RPE1 GFP-CETN1 cells (Fig. 5b, Supplementary Table 1). To investigate the MC uncapping mechanism further we performed higher-resolution SRM stimulated emission depletion (STED) imaging studies. STED imaging revealed that GFP-CP110 removal from the MC can occur in an asymmetric manner, with the remaining MC associated protein showing a “C” shape localization (Fig. 5c). A similar asymmetric uncapping process for CP110 and/or CEP97 was confirmed using SIM (Supplementary Fig. 8b, c) and expansion microscopy (ExM) (Fig. 5d, e, Supplementary Fig. 8d). Together these findings suggest MC cap removal correlates with assembly of tubular membranes during ciliogenesis.

Because MC uncapping is linked to TZ formation^46^ and TZ protein recruitment is associated with the CV stage^7,19^, we compared localization of TZ and MC cap proteins during ciliogenesis. Similar to results with SMO-GFP (Supplementary Fig. 8a), a correlation was observed with the accumulation of the TZ protein GFP-B9D2 at the MC and progressive CP110 removal by confocal imaging (Supplementary Fig. 8e). SRM SIM imaging studies suggested different MC localization of CP110 or CEP97 and the soluble TZ protein GFP-B9D2 in RPE1 cells (Supplementary Fig. 8b, c). Using higher-resolution ExM SRM imaging approaches combined with fluorescence profile plotting, MC accumulation of the TZ proteins TMEM67 and RPGRIP1L could be observed at sites lacking CP110 (Figs. 5d, e and Supplementary Fig. 8d, f). In addition, MCs with SMO-GFP donut-like structures observed by SIM imaging, suggestive of pre-axonemal structures, displayed TZ proteins CEP290 and TMEM67 accumulation either within or at the edge of the GFP-positive ‘donut-hole’, respectively (Supplementary Fig. 8g), consistent with the expected position of the TZ on the BB in ciliated cells. Together these findings indicate that MC cap removal correlates with assembly of tubular CCV and TCV structures, and this process is coordinated with recruitment of TZ proteins associated with these membranes during ciliogenesis.

### EHD1 tubulogenic membrane properties direct MC uncapping

**Fig. 6:**
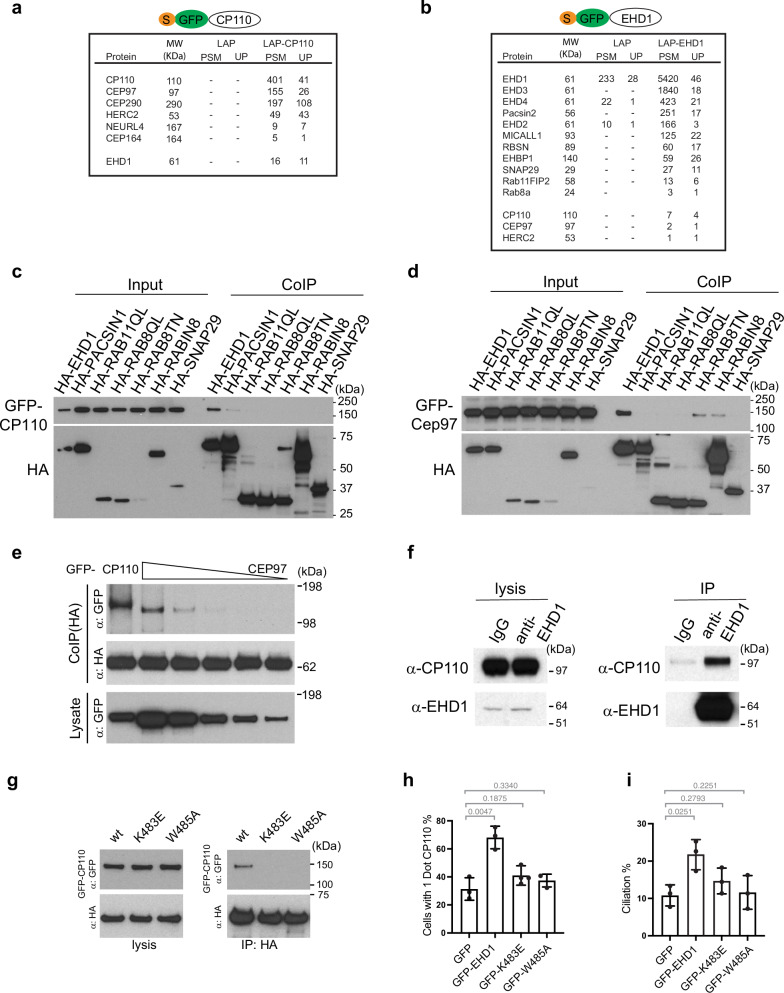
EHD1 promotes MC uncapping through its membrane tubulation function. **a** 293T cells stably expressing LAP (S-tag-TEV-GFP) and LAP-CP110 were immunoprecipitated and interacting proteins were identified by MS. **b** 293T cells stably expressing LAP, LAP-EHD1 were immunoprecipitated and interacting proteins were identified by MS. **c,** **d** EHD1 interacts specifically with CP110 and CEP97. Co-immunoprecipitation of HA-tagged ciliogenesis membrane trafficking regulators with GFP-CP110 (**c**) and GFP-CEP97 (**d**) transiently transfected in 293T cells. GTP-locked RAB11A-Q70L and RAB8A-Q67L and GDP-locked RAB8A-T22N. **e** EHD1 binds more strongly with CP110 than CEP97. 293T cells were transfected with plasmids for HA-EHD1 with GFP-CP110 or HA-EHD1 and different amounts of GFP-CEP97. Lysates were immunoprecipitated with anti-HA antibodies. The first and second lanes were transfected with the same amounts of GFP-CP110 or GFP-CEP97 plasmids. **f** Endogenous interaction between EHD1 and CP110. Immunoprecipitation was performed with EHD1 antibodies or rabbit IgG as a control in 293T cells. Blots were probed with EHD1 and CP110 antibodies. **g** Immunoprecipitation studies demonstrate that wild-type HA-EHD1 but not membrane tubule association deficient HA-EHD1-K483E or membrane association deficient HA-EHD1-W485A mutants interact with GFP-CP110. Proteins were transiently expressed in 293T cells and immunoprecipitation was performed with HA-beads. Proteins were detected using GFP and HA antibodies. **h,** **i** Wild-type EHD1, but not EHD1-K483E or -W485A, promotes CP110 removal from the MC (**h**), and ciliation (**i**). RPE1 cells were transiently transfected with GFP, GFP-EHD1, GFP-EHD1 K483E or GFP-EHD1 W485A plasmids for 48 h and stained with the cilia marker ^Ac^tub and CP110 antibodies. Cells where CP110 has been removed from the MC (cells with 1 CP110 dot) are plotted in (**h**). Mean ± SD (three independent experiments, n: GFP = 274 cells, GFP-EHD1 = 302 cells, GFP-K483E = 282 cells, GFP-W485A = 297 cells), GFP-EHD1 wild-type and mutants were compared to GFP control, two-tailed *t*-test. Representative image from three independent experiments (**c–g**).

Based on the observation that MC uncapping correlates with ciliogenesis membrane structural organization, we hypothesized that proteins associated with these membranes may function in the removal of CP110 and CEP97 from the MC. To investigate this theory, we performed immunoprecipitation (IP) mass spectrometry (MS) studies using 293T cells expressing control LAP and LAP-CP110. Peptides for known CP110 interactors CEP97, the CP110 MC uncapping machinery HERC2 and NEURL4^33,47–49^, and CEP290^50^ were detected in the LAP-CP110 IP-MS analysis, but not in the LAP sample (Fig. 6a). Notably, peptides for EHD1 were only present in the LAP-CP110 samples (Fig. 6a). We further confirmed EHD1 interaction with CP110 in reciprocal IP-MS studies comparing cells expressing LAP-EHD1 and control LAP (Fig. 6b). Additionally, peptides for CEP97 and HERC2, an E3 ligase involved in ubiquitin-dependent degradation of CP110 during ciliogenesis^33^, were identified in LAP-EHD1 IP-MS samples. Immunoblot analysis of co-immunoprecipitation studies confirmed GFP-CP110 and GFP-CEP97 can interact with HA-tagged EHD1, whereas other ciliogenesis membrane trafficking regulators tested showed weak or negligible interaction with the MC cap proteins (Fig. 6c, d). Comparison of EHD1 affinity for MC cap proteins showed a stronger interaction with CP110 than CEP97 (Fig. 6e). Further biochemical characterization showed an endogenous interaction between EHD1 and CP110 in 293T cells (Fig. 6f). To test if the EHD1-CP110 interaction is influenced by EHD1 association with tubular membrane structures, we conducted co-immunoprecipitation studies with mutant EHD1-K483E, which does not associate with tubular membranes^51^. Importantly, this mutant localizes to the MC but fails to rescue CP110 removal in EHD1-knockdown cells^7^. HA-tagged EHD1-K483E showed undetectable binding to GFP-CP110 compared to the wild-type protein (Fig. 6g), indicating that EHD1 membrane tubulation function or lysine 483 is essential for interaction with CP110. Similarly, the EHD1 EH-domain W485A mutant, which cannot associate with membranes or function in CP110 removal in EHD1-knockdown cells^7,52^, also failed to bind CP110, further suggesting its membrane tubulation function is important for MC uncapping (Fig. 6g). Based on these results, we hypothesized that EHD1 at MC-associated tubular membranes controls CP110/CEP97 removal to promote ciliogenesis initiation. Moreover, this uncapping process may be directly regulated by increased trafficking of EHD1 to the MC observed in cells undergoing ciliogenesis^7^. To test this theory, we investigated the effects of GFP-EHD1 overexpression on ciliogenesis initiation in serum-fed cells. We found that ~70% of GFP-EHD1 transiently expressing RPE1 cells lost CP110 from the MC, compared to ~30% in GFP alone expressing cells (Fig. 6h). Moreover, GFP-EHD1 expression promoted a significant increase in ciliation in RPE1 cells compared to GFP alone (Fig. 6i). In contrast, the GFP-EHD1 K483E and W485A mutants did not significantly affect CP110 uncapping or ciliation compared to GFP expression (Fig. 6h, i). However, not all RPE1 cells observed to have lost CP110 from the MC presented with cilia. This could be attributed to the asynchronous nature of ciliogenesis or to cilia resorption occurring in cells cultured with serum. Alternatively, the levels of transiently expressed EHD1 may be insufficient to drive ciliogenesis progression in all transfected cells. Indeed, ciliogenesis progression prior to the ciliary axoneme growth stages is suggested by the observed partial colocalization of SMO-GFP with transiently expressed HA-EHD1 on CCV-like structures in RPE1 cells that lacked a ciliary SMO-GFP signal in the central region of the MC (Supplementary Fig. 9). Collectively, these results support functioning of EHD1 on tubular CCV membranes in directing MC uncapping during ciliogenesis via biochemical association with CP110 and CEP97.

### RAB8 is important for ciliogenesis at the CCV stage

**Fig. 7:**
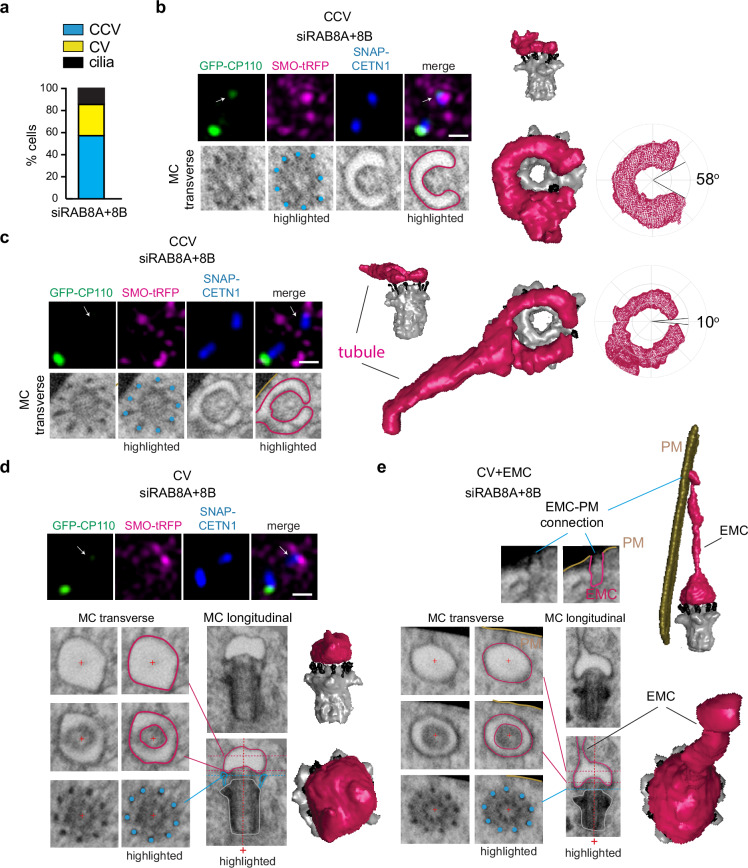
RAB8 is important for ciliogenesis at the CCV stage. **a** RAB8 requirements at the CCV to TCV stage in ciliogenesis. Plot shows ciliary structures identified in RPE1 cells (7 cells) expressing GFP-CP110, SMO-tRFP and SNAP-CETN1 (shown in Supplementary Table 1) treated with siRNAs for 72 h to knockdown RAB8A and RAB8B, with serum starvation for the last 24 h. **b,** **c** SIM images of cells described in (**a**) showing GFP-CP110 removal from the MC (top panel immunofluorescence images). FIB-SEM images show transverse sections of MC with CCV structures (highlighted in magenta) on top of the MC DAs (highlighted with cyan dots). 3D segmented structures are shown along with the corresponding CCV gap determination map (right images). **d** CV structure identified in RAB8-knockdown cells described in (**a**) with complete CP110 removal from the MC. Longitudinal and transverse FIB-SEM images are shown with and without highlighted structures (DA cyan, CV magenta, MC grey) and the 3D CV structure reconstruction. Plus (+) is a positional marker for the transverse section of the MC shown. **e** Identification of a CV with an EMC structure identified in RAB8-knockdown cells described in (**a**). vEM images and highlighted structures as described in (**d**) showing the EMC connection to the PM (EMC-PM), (+) as described in (**d**). Scale bars for confocal images: 1 μm. Representative image from three independent experiments (**b–e**).

Genetic ablation studies demonstrated that RAB8 isoforms (RAB8A and RAB8B) are not important for MC uncapping in RPE1 cells, which is consistent with TEM results supporting a block in ciliogenesis at the CV stage^7^. However, DAV structures were also described^7^, raising the possibility that DAVs and/or CVs identified by TEM imaging could be misidentified tubular membrane intermediates. To clarify RAB8 ciliogenesis function, we performed FIB-SEM on serum-starved RPE1 cells described in Fig. 5a after treatment with RAB8 siRNAs that inhibit ciliogenesis^7^. Cells lacking cilia were selected for FIB-SEM by CLEM using SMO-tRFP and SNAP-CETN1 markers, without screening for GFP-CP110 levels on the MC. FIB-SEM imaging data revealed that the majority of cells had CCVs (Fig. 7a–e, Supplementary Fig. 10a, b and Supplementary Table 1), which could potentially be misidentified as CVs by TEM depending on the sectioning plane. This confirms our concern that 2D TEM imaging can misidentify ciliogenesis intermediates. The observation of cells with CV or a cilium likely reflects incomplete siRNA-mediated depletion of RAB8 isoforms. The absence of accumulated DAVs in these cells indicates that RAB8 is dispensable for the initial DAV-to-CCV transition. Remarkably, a CCV structure was discovered with a very small “C” gap (10°) that resembled an nearly completed toroidal membrane (Fig. 7c), which supports the CCV to TCV assembly mechanism in ciliogenesis. These FIB-SEM analyses indicate that RAB8’s initial function in ciliary assembly is associated with CCVs, while stalled ciliogenesis at the CV stage supports a downstream requirement for RAB8 in axoneme extension. RAB8 functioning on tubular structures upstream of CV establishment is supported by the observation that RAB8 localizes with EHD1 on elongated membranes at the MC during ciliogenesis^19^. However, RAB8’s function at the CCV stage appears to be unrelated to organizing membranes into tubular structures since RAB8 depletion does not affect this structure’s assembly. This conclusion is further supported by the fact that some RAB8-knockdown cells showed an extended membrane tubule from the CCV and an EMC associated with a CV (Fig. 7c, e, Supplementary Fig. 10a, b and Supplementary Table 1).

As expected, MC uncapping occurs in RAB8-knockdown cells as GFP-CP110 was partially or completely removed from the MC in all RAB8 RNAi treated cells examined by CLEM SRM FIB-SEM (Fig. 7a–e). Furthermore, for the nearly completed toroidal membrane structure GFP-CP110 was undetectable (Fig. 7c), while in cells with larger “C” gaps some GFP-CP110 remained (Fig. 7b). These observations support requirements for tubular membrane assembly in instructing MC uncapping and suggest that RAB8-dependent CCV “C” gap closure upstream of TCV assembly is associated with CP110 removal.

### IFT88 functions at pre-CV tubular membrane assembly stages during ciliogenesis

**Fig. 8:**
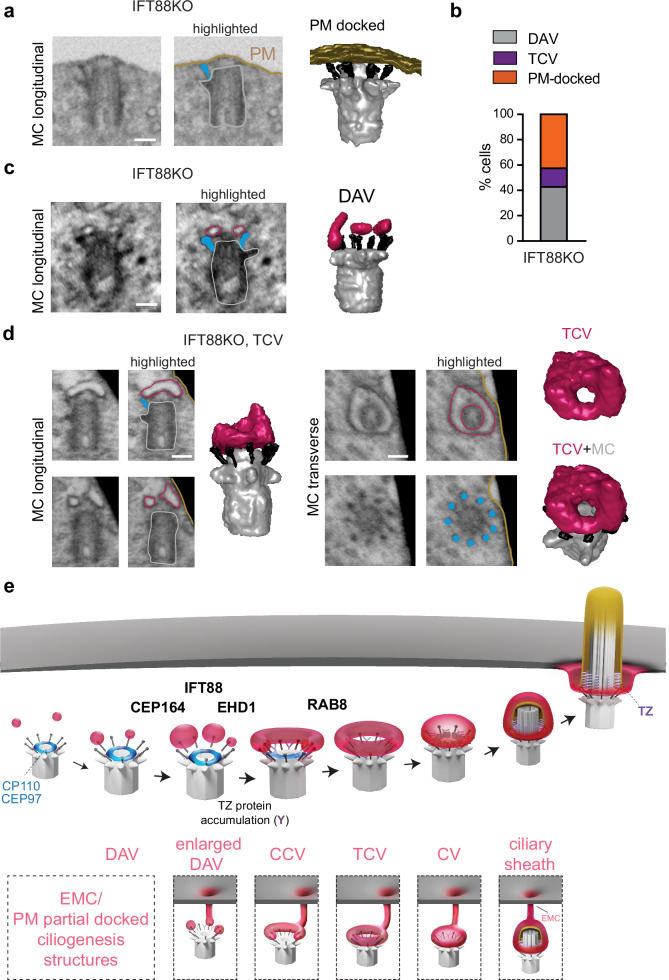
IFT88 is important at tubular membrane intermediate stages of ciliogenesis upstream of axoneme growth. **a–d** Characterization of ciliogenesis impairment in IFT88 KO cells expressing GFP-CETN1 by vEM. Ciliogenesis structures identified in RPE1 cells lacking IFT88 after 24 h starvation. FIB-SEM and segmentation images showing representative PM-docked (**a**), DAVs (**c**), and a TCV (**d**) from 7 cells (shown in Supplementary Table 1) with MC structures plotted in (**b**). Highlighted structures shown on FIB-SEM images as described in Fig. 1g. Scale bars: 200 nm. Images are from one experiment. **e** Schematic of membrane organization in intracellular ciliogenesis pathway.

Roles for the IFT-B complex in ciliogenesis upstream of the CV stage are suggested based on the temporal accumulation of these proteins at the MC during ciliogenesis^7,16,19^. Notably, RNAi knockdown of IFT88 in RPE1 cells did not prevent SMO-GFP from localizing to the MC during ciliogenesis even though most cells failed to develop cilia^53^. To investigate IFT-B complex pre-axonemal ciliogenesis function in RPE1 GFP-CETN1 cells, we performed FIB-SEM imaging on IFT88 KO cells which could not form cilia (Supplementary Fig. 11a–c), an effect rescued by the expression of mScarlet-IFT88. A critical IFT88 function in axoneme growth was confirmed by FIB-SEM analysis of 24 h serum-starved IFT88 KO cells which revealed that ciliogenesis did not progress beyond MC docking to the PM (Fig. 8a, b). Strikingly, more than half of the cells imaged displayed ciliogenesis defects associated with earlier steps, particularly at the DAV stage (Fig. 8b, c) and one cell showed a TCV structure docked to the MC (Fig. 8b, d, and Supplementary Fig. 11d). These results suggest the kinetics of ciliary membrane development are affected prior to MC docking to the PM, linking IFT88 function to tubular membrane organization on the MC at pre-CV assembly stages of ciliogenesis.

## Discussion

Defects in membrane trafficking associated with ciliogenesis are causative in human ciliopathies^54–57^. The first description of membrane assembly at the MC distal end during ciliogenesis was reported over 60 years ago using TEM^13^. However, the function and organization of these membranes during ciliary biogenesis has been enigmatic, limited by the investigative approaches available to researchers. The recent integration of vEM imaging approaches, specifically FIB-SEM, into the field of biology has unlocked the potential for directly visualizing cellular ultrastructure at low nanometer resolutions^37,38^. Only a few studies have employed quantitative analysis of cellular structures by vEM^58–61^, and here we show this approach can be used to understand organelle biogenesis dynamics in the low nanoscale range. Recognizing the limitations of static EM in establishing strict temporal order, we leveraged the asynchronous nature of ciliogenesis resulting from serum-starvation and performed FIB-SEM on two different cultured cell types. To further enrich for early stages of this process, we used knockout/knockdown of ciliogenesis regulators in RPE1 cells. Moreover, we incorporated CLEM with FIB-SEM and examined fixed and live SRM imaged cells to strengthen dynamics correlations for membrane-MC ciliogenic processes. Together these studies revealed new insights into the intricate steps of MC-associated vesicle docking, fusion and reshaping, along with establishing new mechanistic connections to MC uncapping and TZ protein recruitment function in intracellular ciliogenesis in RPE1 and human fibroblasts (Fig. 8e).

Using FIB-SEM, we show it is possible to spatially resolve ciliogenesis-associated structures isotropically at 10 nm resolution. This enabled the quantitative characterization of different ciliogenesis membrane intermediates through examining their frequencies, sizes, and distributions on the MC, not possible using TEM imaging of ~70–80 nm sections. Our findings support a mechanism in which increased membrane docking of DAVs to the MC occurs during ciliogenesis, followed by the expansion of DAVs, which we link to the DAP CEP164. The membrane shaping and fusion regulator EHD1 was found to be required for the efficient formation of tubular CCV membranes from enlarged DAVs. Interestingly, vEM studies on differentiating mouse granule cells showed tubular membranes associated with the MC attributed to resorption of cilia, suggesting a similar membrane organization process could function in both assembly and disassembly of the cilia^40^. Our vEM analysis also redefines RAB8 and IFT88 ciliogenesis functioning in association with assembly of tubular membrane intermediates at the MC. Specifically, our results suggest that RAB8 functions on CCVs, possibly through regulating membrane fusion. Our finding that IFT88 is also important for efficient progression of ciliogenesis upstream of ciliary axoneme growth merits additional investigation. This is consistent with the presence of the IFT-B complex at the centrosomes in non-ciliating cells^62,63^. IFT-B components have been linked to pre-axoneme ciliogenesis processes in multiciliated cells (MCCs)^64^, although it remains unclear if MCCs utilize a similar ciliary assembly mechanism as primary ciliated cells. Notably, FIB-SEM imaging in Rab34 knockout RPE1 cells^5^ suggests a role for this membrane trafficking regulator upstream of CV assembly, although comparison of docking and vesicle size parameters will be necessary to more precisely resolve this protein’s role in the ciliogenesis as specified in this study.

This study also provides new insights into the connection between ciliary membrane formation and MC to BB transition, specifically through the removal of the CP110/CEP97 MC cap in cells that utilize the intracellular ciliogenesis pathway. Our findings suggest that EHD1 can form a complex with CP110 and CEP97, facilitating the removal of these proteins from the MC through its membrane tubulation associated function. One potential mechanism for this coordination involves the recruitment of HERC2 to the MC by EHD1, which promotes the ubiquitination of CP110^33^. Interestingly, CP110 has been shown to inhibit the function of CEP290 in TZ formation^50^. This aligns with our observations that TZ proteins accumulate only where CP110 and CEP97 have been removed from the MC distal end.

The mechanisms governing the assembly and functionalization of the multiprotein transition zone (TZ) complex, comprising soluble and transmembrane proteins essential for regulating ciliary transport, remains poorly understood. This knowledge gap is highly significant as TZ proteins are frequently mutated in human ciliopathy^1,2^. Our finding showing the transmembrane TZ protein TMEM67 on CCV-like structures suggests this protein is recruited on preciliary membranes targeted to the MC. However, it is also possible EMCs mediate TMEM67 trafficking to MC associated membranes, as well as other TZ proteins and ciliogenesis factors. Importantly, our findings support a model where EMCs mediate MC connections to the PM^19^, forming from CCV and possibly earlier DAV membranes. Our observations that TZ proteins accumulate at sites of CP110 loss associated with CCV/TCV structures suggest that the TZ is organized at these stages, however, it is not clear if the functional Y-link architecture of the TZ is established^65^. Live SRM analysis of ciliogenesis in RPE1 cells showed that SMOM2-GFP exhibited a stronger consolidated localization at the center of the MC, consistent with the establishment of the ciliary membrane, axoneme and a functional TZ. Interestingly, the concave surface of the CCV and TCV membranes exhibit curvature similar to that of the ciliary membrane, and the TCV encircles the MC like the ciliary membrane. Thus it is conceivable that these tubular ciliogenesis membranes may be organized similarly to the mature ciliary structure, complete with a TZ capable of regulating the targeting of proteins critical for ciliary growth, including axonemal assembly factors such as molecular motors, microtubule-associated proteins (MAPs) and EB1/3^66,67^. It will be interesting to determine whether the donut-shaped membrane shares a similar phospholipid composition with the ciliary membrane, which in the cilia is regulated by INPP5E that converts PtdIns(4,5)P2 into PtdIns(4)P^68^. Notably, within the cilium, the membrane exhibits negative curvature along its length, transitioning to positive curvature at its base into the cytoplasmic face of the ciliary pocket (CP) membrane. Ciliogenic EHD and PACSIN family proteins, known to be associated with positive-curvature membranes^69,70^, localize to the CP, but not within the cilia^19^. This further suggests a partitioning mechanism by the TZ at the CCV/TCV stages that helps facilitate the establishment of the negatively curved ciliary membrane as ciliogenesis progresses.

To our knowledge, naturally occurring toroidal membrane vesicles have not been documented in cells. Modeling predictions suggest that shaping membrane tubules into C-shaped and toroidal structures is highly energetically unfavorable^71^, particularly for smaller membranes such as those observed at the MC during ciliogenesis. Specifically, the final fusion of the C-shaped ends to form the toroid is the highest barrier energy state in these models. In ciliogenesis, organization of these tubular structures is likely aided by anchoring membranes to the DAs through protein-protein interactions. These MC-membrane connections likely reduce the energy barriers affecting the stability and formation of tubular structures, thereby reducing the energy burden associated with membrane bending. Additionally, structural support for membrane curvature may be enhanced by the establishment of the TZ, with this Y-link structure extending from the membrane to the MC. Our findings suggest a compelling role for RAB8 in balancing bending energy and the release of osmotic potential energy during the closure step of CCV to form TCV. However, RAB8 function could simply be associated with regulating final DAV docking in the “C”-shape gap or in their fusion with the CCV to generate the TCV. RAB8 function on CCVs could also be associated with the TZ, as it is known to interact biochemically with the core TZ protein CEP290^50^. Overall, our work indicates roles for proteins on the membrane and at the distal end of the MC in establishing the membrane curvature of CCV and TCV structures in cells using the intracellular ciliogenesis pathway.

Lastly, our findings shed new light on the MC-membrane docking regulation and ciliogenesis initiation. Previous ciliogenesis models proposed that DAPs function in the initial membrane docking to the MC^12,16,17^, recruitment of the kinase TTBK2 for MC uncapping^72,73^ and in RAB8-positive membrane growth of ciliary membrane in concert with the axoneme formation^11,41,74,75^. Interestingly, our detailed analysis of vesicle docking and the size of these membranes reveals that small vesicles can dock to the DAs without facilitating ciliogenesis in RPE1 cells. These DAVs could be missed by single TEM image analysis of MCs (Fig. 1g, h), partially because not all DAs contain these docked vesicles. It remains unclear whether these vesicles are stably docked or dynamically associated with the MC DAs. Our findings suggest that ciliation is triggered by the enlargement of DAVs and their organization into tubular membranes that wrap the MC DAs, a process requiring CEP164 and EHD1. The observed partial MC docking to the PM in cells lacking CEP164 suggests that this protein plays a previously unrecognized role in ciliary membrane organization, potentially through the recruitment of membrane shaping and/or fusion factors. These findings could have implications for understanding human ciliopathies associated with CEP164^55,76^. While the majority of the axoneme-less RPE1 cells, regardless of serum treatment, and all the human fibroblasts displayed ciliogenic membrane structures consistent with the intracellular ciliogenesis pathway, the observed partial or fully docked MC to the PM could suggest that RPE1 cells also employ the extracellular ciliogenesis pathway^6^. An alternative explanation for these ciliogenesis structures is that DA docking with the PM requires at a minimum DAVs on the DAs to mediate fusion with the PM, rather than direct DA-PM docking. This could involve the tubulation of DAVs and the formation of an EMC similar to those observed on CCVs and TCVs. Moreover, we did not observe EMCs on DAVs in EHD1-knockdown cells, supporting a role for this protein in the developing primary cilium gaining access to the extracellular space^19^. The frequent observation of EMC structures and partially PM-docked centrioles also points to a mechanism in which tubular membrane organization formed on the MC in the cytoplasm is maintained once the CCV/TCV docks to the PM. This is consistent with the toroidal membrane structure at the base of the ciliary sheath and cilium. This proposed mechanism, along with the infrequent observation of CVs in wild-type cells, raises questions about requirements for CV in ciliogenesis. One possibility is that CVs are only observed in cells where the uncapped MC was unable to establish or fully complete docking to the PM, as may be the case in RAB8-knockdown cells. This DA-membrane docking mechanism to the PM could also explain the range of ciliogenesis structures from DAV to TCV to MC-PM docked stages observed in IFT88 KO cells, although it remains to be determined how IFT88 affects pre-axonemal membrane organization at the MC.

In this study, we have achieved a more comprehensive understanding of the spatial and temporal control of ciliary membrane assembly at the MC in cells that utilize the intracellular ciliogenesis pathway (Fig. 8e). The advanced imaging approaches employed permit more precise identification of membrane structure organization and functioning of key regulators in ciliogenesis, including MC DAPs, capping proteins, membrane trafficking regulators and the IFT-B complex. We expect this work will prompt new investigations aimed at further characterizing the function of these and other ciliogenesis factors, informed by the architectural model presented. This study highlights the immense potential of utilizing vEM techniques and quantitative 3D structural analysis to unravel intricate isotropic complexities associated with the biogenesis of the cilium and other organelles.

## Methods

### Antibodies and reagents

Commercial antibodies used were as follows: anti-Acetylated tubulin (Actub, clone 6-11B-1, 1/10000, T6793, Sigma), β-Actin−Peroxidase antibody (clone AC-15, 1/30000, A3854, Sigma), anti-EHD1 (EPR4954, 1/500, ab109311, Novus Biologicals), anti-RPGRIP1L (1/200, 55160-1-AP, Proteintech), anti-TMEM67 (1/200, 13975-1-AP, Proteintech), Rabbit anti-CEP164 (1/500, 22227-1-AP, Proteintech), chicken anti-CEP164^19^, Rabbit anti-CP110 (1/1000, 12780-1-AP, Proteintech), mouse anti-CP110 (1/500, MABT1354, Millipore Sigma), anti-CEP97 (1/1000, A301-945A, Bethyl), anti-GFP Alexa 488 (1/1000, A21311, Molecular Probes Life Technologies), DAPI (1/2000, 62248, Thermo Scientific), Hoechst (1/3000, H3570, Molecular Probes Life Technologies) Rabbit anti-Pericentrin (1/2000, NB100-61071, Novus) and Mouse anti-Arl13b (1/500, N295B/66, NeuroMab) and all Alexa Fluor Dyes conjugated secondary antibodies were from Life Technologies. Goat anti-chicken IgY CF640R (Biotium, 20084), goat anti-rabbit CF568 (Biotium, 20099) and goat anti-mouse CF488 (Biotium, 20010) were used for ExM staining. SNAP-Cell647-SiR reagent was purchased from New England Biolabs. Atto594 and Atto647N conjugated secondary antibodies for STED imaging were from Millipore-Sigma. Doxycycline hydrochloride was obtained from Sigma and used according to manufacturer’s instructions.

### Cell lines

Human hTERT-RPE1 (CRL-4000) and 293 T (CRL-3216) cell lines were obtained from ATCC. The human fibroblast cell line was obtained and cultured as previously described^77^. hTERT-RPE1 cells were cultured in DMEM/F-12 supplemented with GlutaMAX and 10% FBS; 293T cells were cultured in DMEM supplemented with GlutaMAX and 10% FBS; human fibroblasts were cultured in MEM α supplemented with L-Glutamine, 10% FBS and 100 µg/mL Primocin. During cell culture, subculturing was performed when the cell growth reaches 80%–90% confluence. LAP, LAP-EHD1, GFP-CETN1, SMO-GFP, SMOM2-GFP, GFP-B9D2, SMO-tRFP and LAP-EHD1 + SMO-tRFP RPE1 cell lines were generated using lentivirus or Flp-In system as previously described^7,19,78^. The SMOM2-GFP + SNAP-CEP83 cell line was generated by introducing SNAP-CEP83 lentivirus into the SMOM2-GFP cell line. For SIM FIB-SEM CLEM studies, RPE1 cells were infected with LAP-CP110, SMO-tRFP^7^, and SNAP-CETN1^19^ viruses and single-cell cloning performed to isolate a clonal cell line expressing all three reporter proteins. Human fibroblasts were infected with GFP-CETN1 lentivirus to generate a stable cell line. 293T cells expressing LAP and LAP-EHD1 or LAP-CP110 were stably generated using lentiviral transduction as described^78^. DNA transfections were carried out using X-tremeGENE 9 (Roche). LAP, LAP-EHD1 and LAP-CP110 expression was induced with 0.5 μg/mL doxycycline^7^. For knockdown experiments, cells were transfected with siRNA duplexes (Dharmacon) using RNAiMAX (Invitrogen) according to manufacturer’s instructions and were fixed for analysis after 72 h treatment.

### Immunofluorescence

To promote ciliogenesis, cell lines were serum-starved at ~70% confluence for 3, 6 or 24 h. After serum starvation, cells were fixed using 4% paraformaldehyde or cold methanol for 10 min. Blocking was performed for 10 min with 1% BSA in PBS 0.1% Triton X-100 or Saponin, followed by immunostaining in blocking solution with indicated antibodies in figure legends. Confocal images were taken using a Marianas spinning disc (SD) confocal microscope (Intelligent Imaging Innovations) equipped with a 63 × 1.4 NA objective and a CMOS camera (ORCA-Fusion, Hamamatsu). Cellular labeling was done using SNAP-Cell647-SiR (New England Biolabs) substrate according to the manufacturer’s recommendation and as described in the figure legends. Ciliogenesis and CP110 uncapping were quantified as described^7^.

### Structured illumination microscopy (SIM)

The coverglass with fixed cells was incubated with 100 nm TetraSpeck beads (Life Technologies) in the final wash and mounted onto glass slides using ProLong Glass Antifade Mountant (ThermoFisher). 3D-SIM imaging was conducted using a Nikon N-SIM microscope, a GE DeltaVision OMX SR imaging system, or a Zeiss Elyra 7 SIM microscope, as indicated in the figure legends. Specifically, Nikon SIM images were captured with a SR Apo TIRF × 100/1.49 NA oil immersion objective from Nikon and an EMCCD camera (Andor DU-897E), while Zeiss SIM images were obtained with a 63 × 1.4 NA oil objective from Zeiss and a PCO edge sCMOS camera, as previously described. The image stacks were acquired at a z-distance of 0.1 μm, and alignment parameters for all color channels were carefully determined during the calibration process using TetraSpeck beads. The Nikon Elements or Zeiss Zen software was utilized for image reconstruction and processing, and TIFFs were edited using FIJI. Intensity profiles were generated following established protocols.

### STED microscopy

STED imaging utilized the Leica TCS SP8 STED 3× system, which is equipped with a white light laser for excitation. The imaging employed a ×100 oil-immersion objective from Leica with a numerical aperture (N.A.) of 1.4. For dual color STED imaging, Atto594 and Atto647 conjugated secondary antibodies were used for imaging.

### Modified protein-retention expansion microscopy (pro-ExM)

The ExM protocol generally followed proExM methods^79^ with modifications to improve uniformity of centrosome expansion. The acrylamide concentration was increased as described in^80^, and EDTA concentration was increased as described in^81^. Briefly, cells grown on coverslips were fixed with 4% paraformaldehyde for 10 min and subjected to immunofluorescence staining with primary and secondary antibodies as described above with CP110, RPGRIP1L, TMEM67 and CEP164 antibodies. The coverslips were then incubated with Acryloyl-X (Life Technologies) solution for 18 h and subjected to gelation (8.6% Sodium acrylate (Pfaltz & Bauer), 20% acrylamide (Sigma), 0.15% N,N′-methylenebisacrylamide (Sigma), 2 M NaCl in 1× PBS supplemented with 0.2% APS (Sigma) and 0.2% v/v TEMED (Sigma)). The gel was digested with proteinase K in digestion buffer (0.5% Triton X-100, 25 mM EDTA, 800 mM NaCl, 50 mM Tris) for 18 h and stained with DAPI before being placed in ddH2O. An expansion factor of 4.2 was obtained. After expansion, gels were imaged with Yokogawa W1 SD on a Leica DMi8 microscope using a 63× NA1.2 water immersion lens. Images were acquired with the MetaMorph® Microscopy Automation and Image Analysis Software (Molecular Devices) and processed using Fiji.

### Ultrastructure expansion microscopy (U-ExM)

U-ExM samples were prepared as previously described^82^. Briefly, cells grown on 12 mm coverslips were fixed and incubated with 1.4% formaldehyde and 2% acrylamide in PBS for 5 h at 37 °C, followed by gelation in a solution containing 23% sodium acrylate, 10% acrylamide, and 0.1% *N*,*N*′-methylenebisacrylamide in 1× PBS, supplemented with 0.2% TEMED and 0.2% APS. Samples were then denatured in denaturation buffer (200 mM NaCl, 200 mM SDS, 50 mM Tris, pH 9.0) for 90 min at 95 °C, and subsequently stained with antibodies and DAPI as indicated in the figures. After expansion, images were captured using a Yokogawa X1 SD confocal on a 3i Marianas microscope equipped with a 100× NA 1.46 oil immersion lens. An expansion factor of 4.3 was obtained using this method. Images were processed using Fiji.

### Time lapse SIM^2^ microscopy

Super-resolution SIM^2^ live-cell imaging was performed on a Zeiss Elyra 7 SIM^2^ system, with the environmental chamber maintaining a temperature of 37 °C supplemented with 5% CO2. RPE1 cells expressing SMOM2-GFP and SNAPf-CEP83 were incubated with SNAP-Cell 647-SiR in culture medium for 30 min before changing to FluoroBrit DMEM without FBS and placed in the environmental chamber. To conduct Apotome SIM time-lapse experiments, multiple positions were defined within the position window of the Zen 3 Black software. Images were captured using a 40 × 1.4 NA oil objective with three phases obtained at each time point every 10 min for 18 h using Apotome setting. To capture the MC/BB movement during ciliogenesis, a z-stack of approximately 3 µm was captured using leap mode for each time point. Figures display projections of three to five slices from the stack around the MC/BB. Imaging was processed with SIM^2^ in Zen Black software.

### Fluorescent intensity measurements

To measure the signal intensity of CP110, SMO-GFP, and GFP-B9D2 on the MC from ExM and confocal imaging, a circular region covering the distal appendage marker CEP164 was defined for each cell in the fluorescent images. For SD confocal images the signal intensities of SMO-GFP, GFP-B9D2, and CP110 were quantified using SlideBook software, with camera background subtracted from the measured values. The mean intensity for each marker was plotted in the figures. Similar measurements were performed on ExM images by selecting a circular region inside the DA ring marked by CEP164 using Fiji. The correlation between CP110 and transition zone (TZ) proteins was analyzed using mean intensity values. Fluorescence intensity plot profiles of orthogonal slices of the cell were generated as previously described using Fiji^7^.

### CRISPR Cas9 knockout cell lines

peSpCas9(1.1)−2×sgRNA and peSpCas9(1.1)−2×sgRNA-(IFT88, donor) plasmids used for IFT88 and CEP164 knockout were gifts from Kazuhisa Nakayama (Addgene plasmid #80769 and #80768). CEP164 gRNA was designed as described^42^ and cloned into peSpCas9(1.1)−2×sgRNA. IFT88 and CEP164 knockout RPE1 cell lines were generated as described^83^. Rescue experiments were performed in stable cell lines expressing GFP-CEP164 or mScarlet-IFT88.

### CLEM sample preparation

Sample preparation was performed as described previously^19^. Briefly, cells were grown on alphanumerically coded gridded coverslips (MatTek) to ~30%–50% confluence with and without serum as indicated. CLEM studies with RPE1 GFP-CETN1 or GFP-CETN1 + SMO-tRFP cells were performed under serum-fed and/or serum-starvation (3 or 6 h) treatments. After fixing, various cells of interest were imaged by SD confocal microscopy with 40× objective to identify ROIs with centrioles and/or elongated cilia. Immediately after fluorescence imaging, bright field images of the gridded pattern containing the cells were acquired using both 40× and 10× objectives to generate an accurate “target map” of candidate cells for interrogation by FIB-SEM. The cell samples were then post-fixed, stained, dehydrated, and embedded in PolyBed (Polyscience) resin according to standard protocols. This allowed the etched alphanumeric pattern to be transferred to the resin. The blocks were then gently cleaned, affixed to an SEM stub with conductive silver paint, and sputter coated with a thin conductive layer of carbon before transferring to the FIB-SEM instrument. For SIM/FIB-SEM, RPE1 cells expressing GFP-CP110/SMO-tRFP/SNAPf-CETN1 were grown in 35 mm glass-bottom dishes with alphanumerically coded gridded coverslips. The cells were serum-starved for 3 h or transfected with EHD1 or RAB8a + RAB8b siRNA for 48 h and serum-starved for an additional 24 h before fixation. Then cells were fixed with 4% paraformaldehyde and 0.25% glutaraldehyde in 0.1 M sodium cacodylate buffer for 30 min at RT and washed with 0.1 M sodium cacodylate buffer three times before imaging. 3D SIM images were collected on a Nikon N-SIM with a 100× objective. Phase contrast images of the target cells and the alphanumerical pattern of the coverslip were taken with both the 100× objective and a 10× objective to generate the “target map”.

### FIB-SEM imaging

FIB-SEM imaging was performed in a Zeiss Crossbeam 550 (Carl Zeiss Inc.) in conjunction with ATLAS3D software (Fibics Inc.), as previously published^19^ with a few modifications. Briefly, a platinum and carbon patterned protective pad was deposited with the FIB operated at 700 pA, and data collection was executed with the FIB and SEM operated simultaneously. The FIB was operated at 30 kV, 700 pA, SEM operated at 1.5 kV, 1 nA, and back scatter signal was recorded at the in-column EsB detector operated with a 900 V grid voltage. The “ROI” images were acquired at 3 nm pixel sampling and 9 nm milling increments, with total dwell time of 3 µs per pixel. An imaging run covering portions of a cell typically lasted ~20 h and generated a stack of ~1000 high resolution images; however, the volume containing the centriolar area was much smaller. These images were registered using in-house IMOD based scripts, and subsequently cropped, binned and inverted to yield registered, isotropic (9 × 9 × 9 nm) volumes in mrc format. These data provided a high-resolution vEM readout corresponding to the targeted cellular features imaged previously by fluorescence, and centrioles could be easily identified without further correlative fiducial markers.

### FIB-SEM segmentation

FIB-SEM reconstructions were analyzed using Dragonfly or 3D Slicer software. 3D volume segmentation models were generated using Dragonfly with ciliary-associated structures (centrioles, ciliary membrane, PM, and membrane tubules) by using a combination of automatic thresholding and manual assignments. To ensure accuracy, segmentation assignments were verified across all three (*xyz*) FIB-SEM image planes. Additionally, the segmentation of structures was independently performed by three to five individuals for validation.

### vEM FIB-SEM image analysis

CCV gap determination was performed on Dragonfly segmentations that were exported as contour meshes and subsequently converted to NumPy arrays of cylindrical coordinates. This array was sorted by ascending θ with the largest difference in θ taken as the C-Shaped Gap. CCVs were characterized as having membrane gaps less than 180°. Membrane distances to DA distal end in cilia, CV, and CCV were measured in 3D for vEM stacks using the Dragonfly ruler tool. For CCV, DA to membrane distances at the CCV gap were not considered in docking distance determinations. Membrane structures were classified as docked DAVs if the structure was present in at least two consecutive 3D planes, larger than 30 nm in diameter, and 30 nm or less from the DA distal end. DA-membrane docking analysis was independently performed by three to five different individuals for validation. The surface area of membrane structures docked to the MC was calculated using a built-in function in Dragonfly.

### Immunoprecipitation

Immunoprecipitations were carried out as described^64^. 293 T cells transfected with specified plasmids were harvested and lysed using a lysis buffer (20 mM Tris-HCl, pH 7.5, 150 mM KCl, 1 mM EDTA, 0.5% NP-40, 10% glycerol, 10 mM sodium pyrophosphate, 3 mM dithiothreitol, and 0.5 mM phenylmethyl sulphonyl fluoride (PMSF, ThermoFisher, 36978) and protease inhibitor cocktail (Sigma, 539134). The resulting cell lysates were clarified through centrifugation at 14,000 g for 10 min at 4 °C. Immunoprecipitation of GFP fusion proteins was performed using GFP-Trap affinity resin (Chromotek, gta-20), while HA-tagged proteins were immunoprecipitated with Pierce Anti-HA magnetic beads (ThermoFisher, 88836). Following four washes with the lysis buffer, the beads were incubated with 65 μL of 2× SDS loading buffer for subsequent western blot analysis. For full scan blots, see Source Data file.

For endogenous immunoprecipitation, 293 T cells were grown to confluency in 10-cm plates. Following a 3 h serum starvation, the cells were lysed using a buffer composed of 50 mM Tris-HCl (pH 7.4), 150 mM NaCl, 1% Triton-X100, 10% glycerol, and a protease inhibitor cocktail. The cleared cell lysate was then mixed with 3 μg of EHD1 antibody or Rabbit-IgG, and the mixture was incubated for 1 h in a cold room. Subsequently, 20 μL of Affi-Prep Protein A Resin (BioRad) was added, and the samples were incubated for an additional 1 h. The beads were washed four times with lysis buffer and subjected to SDS-PAGE gel separation.

### Mass spectrometry (MS)

MS was performed as previously described^84^. Before immunoprecipitation, cells were serum-starved for 3 h and then lysed with a lysis buffer containing 50 mM Tris-HCl (pH 7.4), 150 mM NaCl, 1% Triton-X100, 10% glycerol, and a protease inhibitor cocktail. The cleared cell lysates were mixed with 25 μL of GFP-Trap affinity resin (Chromotek, gta-20) and incubated for 2 h in a cold room. Cells expressing the LAP tag were used as control. The resin was subsequently washed four times with the lysis buffer, mixed with 50 μL of 50 mM NH_4_HCO_3_, and frozen at −80 °C until MS analysis (*n* = 1 for LAP-EHD1 and LAP-CP110, *n *= 2 for LAP control).

For MS analysis, resin beads were resuspended in 50 μL of 25 mM HEPES (pH 8.0) and heated at 95 °C for 10 min. Two μg of trypsin was added to the samples, followed by incubation at 37 °C overnight with constant shaking. The supernatant containing the tryptic digests was collected after centrifugation. The residual beads were washed twice with 25 mM HEPES (pH 8.0), and the supernatant and washes were combined for maximum recovery. Peptides were desalted using C18 columns (Thermo Scientific, CA), lyophilized, and reconstituted in 0.1% trifluoroacetic acid.

LC-MS/MS analysis was performed on a Q Exactive HF mass spectrometer coupled to a Thermo Easy-nLC 1200 system (Thermo Scientific, CA). Liquid chromatography was carried out on an Acclaim PepMap 100 C18 column (Thermo Scientific, CA). Peptides were eluted at a flow rate of 300 nL/min using a 5%–27% gradient of acetonitrile with 0.1% formic acid over 81 min, followed by a 27%–40% gradient over 25 min. MS1 acquisition was performed at a resolution of 60,000 over a mass range of 380–1580 m/z, with a maximum injection time of 120 ms and an AGC target of 3e6. MS2 scans were performed at a resolution of 15,000, with a normalized collision energy of 27, a maximum injection time of 50 ms, and an AGC target of 2e5.

MS files were searched with Proteome Discoverer 2.4 (Thermo Scientific, CA) using the Sequest node. Data were searched against the UniProt human database (2020) using a full tryptic digest, a maximum of two missed cleavages, peptide lengths between 6 and 40 amino acids, an MS1 mass tolerance of 10 ppm, and an MS2 mass tolerance of 0.02 Da. Percolator was used for FDR analysis which was set at 1%.

### Statistics and reproducibility

Statistical analyses were performed using GraphPad Prism for Macintosh OS. Data presented are as specified in the Figure legends or text but generally ±SEM or SD. Two or more group comparisons were carried out using an unpaired, two-tailed Student *t*-test or with one-way ANOVA as indicated. A Fisher’s exact test was used to compare DAV and CCV levels. Figure legends with significant values as follows: *p  <  0.05, **p  <  0.01, ***p < 0.001, ****p < 0.0001, n.s. not significant, *N* number of independent experiments.

### Reporting summary

Further information on research design is available in the Nature Portfolio Reporting Summary linked to this article.

## Supplementary information

Supplementary information

Description of Additional Supplementary Files

Supplementary Movie 1

Supplementary Movie 2

Supplementary Movie 3

Supplementary Movie 4

Supplementary Movie 5

Supplementary Movie 6

Supplementary Movie 7

Supplementary Movie 8

Supplementary Movie 9

Reporting Summary

Transparent Peer Review file

## Source data

Source Data

## Data Availability

The FIB-SEM datasets generated in this study have been deposited in the EMPIAR database under accession code EMPIAR-13463 [10.6019/EMPIAR-13463]. The raw mass spectrometry data have been deposited in the MassIVE database under accession code MSV000101315 [10.25345/C5MK65P08]. Source data are provided with this paper.
