# Supplementary material for: Characterization of membrane structures regulating primary ciliogenesis by quantitative isotropic ultrastructure imaging
Source: Nat Commun. 2026 Jun 13;17:7514. doi: 10.1038/s41467-026-73638-4 (PMC13408954; doi:10.1038/s41467-026-73638-4)
Supplement: Supplementary file 1 — Supplementary information [file 41467_2026_73638_MOESM1_ESM.pdf]

# Supplementary information

## Characterization of membrane structures regulating primary ciliogenesis by quantitative isotropic ultrastructure imaging

Quanlong Lu[1], Huijie Zhao[1], Ziam Khan[1], Adam Harned[2,3], Erina Kamiya[4], Valentin Magidson[4], Abhi Senthilkumar[1], Avaneesh Kilnagar[1], Phuong Thi Bich Doan[1], Sumeth Perera[1], Kedar Narayan[2,3], Christopher J. Westlake[1*]

[1]Laboratory of Cellular and Developmental Signaling, Center for Cancer Research, National Cancer Institute, National Institutes of Health, Frederick, MD 21702, USA

[2]Center for Molecular Microscopy, Center for Cancer Research, National Cancer Institute, National Institutes of Health, Frederick, MD, 21701, USA

[3]Cancer Research Technology Program, Frederick National Laboratory for Cancer Research, Frederick, MD, 21702, USA.

[4]Optical Microscopy and Analysis Laboratory, Cancer Research Technology Program, Frederick National Laboratory for Cancer Research, Frederick, MD, 21702, USA.

* Correspondence: westlakecj@mail.nih.gov

# Supplementary Figure 1

**a**

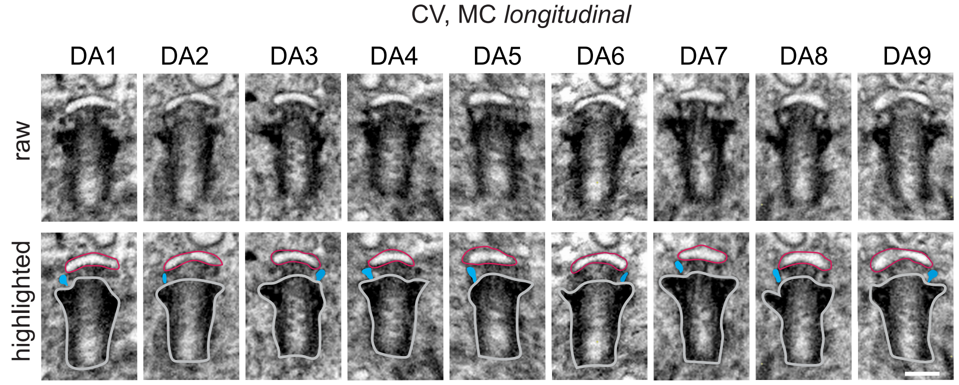


**b**

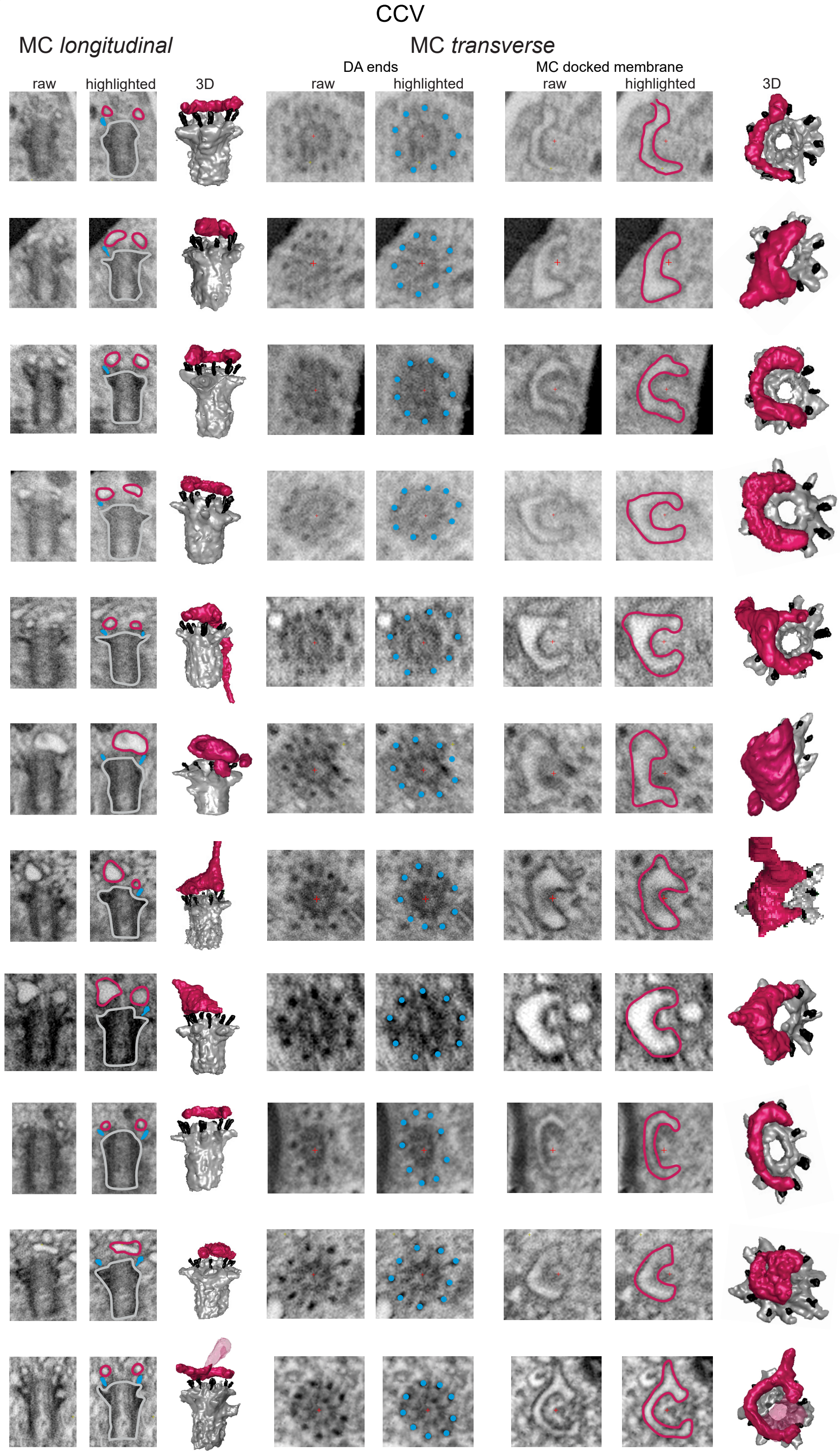


| cell line | serum | tubulogenic | EMC | DAVs |
|---|---|---|---|---|
| RPE1 | + | yes | yes | yes |
| RPE1 GFP-CETN1 | - (3h) | no | no | no |
| RPE1 GFP-CETN1 | - (3h) | no | no | no |
| RPE1 GFP-CETN1 | - (3h) | no | no | no |
| RPE1 GFP-CETN1 | - (3h) | yes | no | yes |
| RPE1 GFP-CETN1 | - (3h) | no | no | yes |
| RPE1 GFP-CETN1 SMO-tRFP | - (6h) | yes | yes | yes |
| RPE1 GFP-CETN1 | - (3h) | yes | yes | yes |
| RPE1 GFP-CETN1 SMO-tRFP | - (6h) | no | no | no |
| fibroblast GFP-CETN1 | - (6h) | no | no | no |
| fibroblast GFP-CETN1 | - (6h) | yes | no | no |

**Supplementary Fig. 1: Cilia and CCV structure reconstruction from FIB-SEM imaging.**

**a** Longitudinal MC FIB-SEM images showing all 9 DAs and CV membrane shown in Fig. 1**d. b** CCV membrane structures identified at the MC in RPE1 and human fibroblast reporter cells grown in serum or starved for 3 or 6h. Transverse vEM images show the CCV structure directly above the DA ends and the longitudinal images show sections through the CCV appearing as DAV or CV structures. The presence of membrane tubule extensions and EMC connections to the PM are indicated for each cell.

Supplementary Figure 2

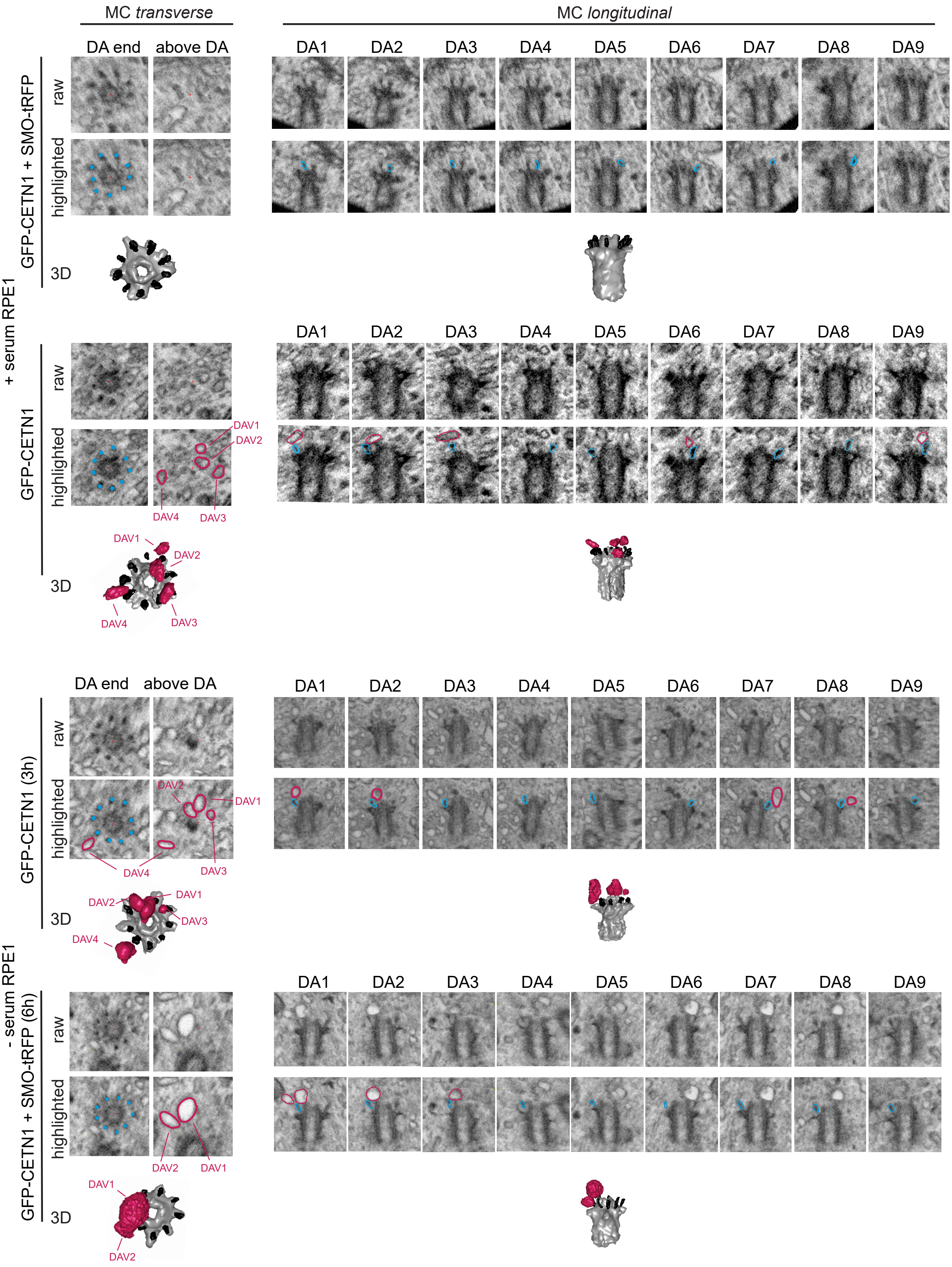

**Supplementary Fig. 2: Determination of DAV docking to the MC.**

FIB-SEM and segmented images showing DAs and docked DAVs in serum-fed and serum-starvation conditions for RPE1 reporter cells described in Fig. 2**c**. Longitudinal FIB-SEM images show each of the 9 DAs and association DAVs and transverse sections show the MC DA ends and associated DA-membrane docking. Highlighted structures DAV (magenta), DAs (cyan) and MC (grey) shown in the unmarked raw image.

# Supplementary Figure 3

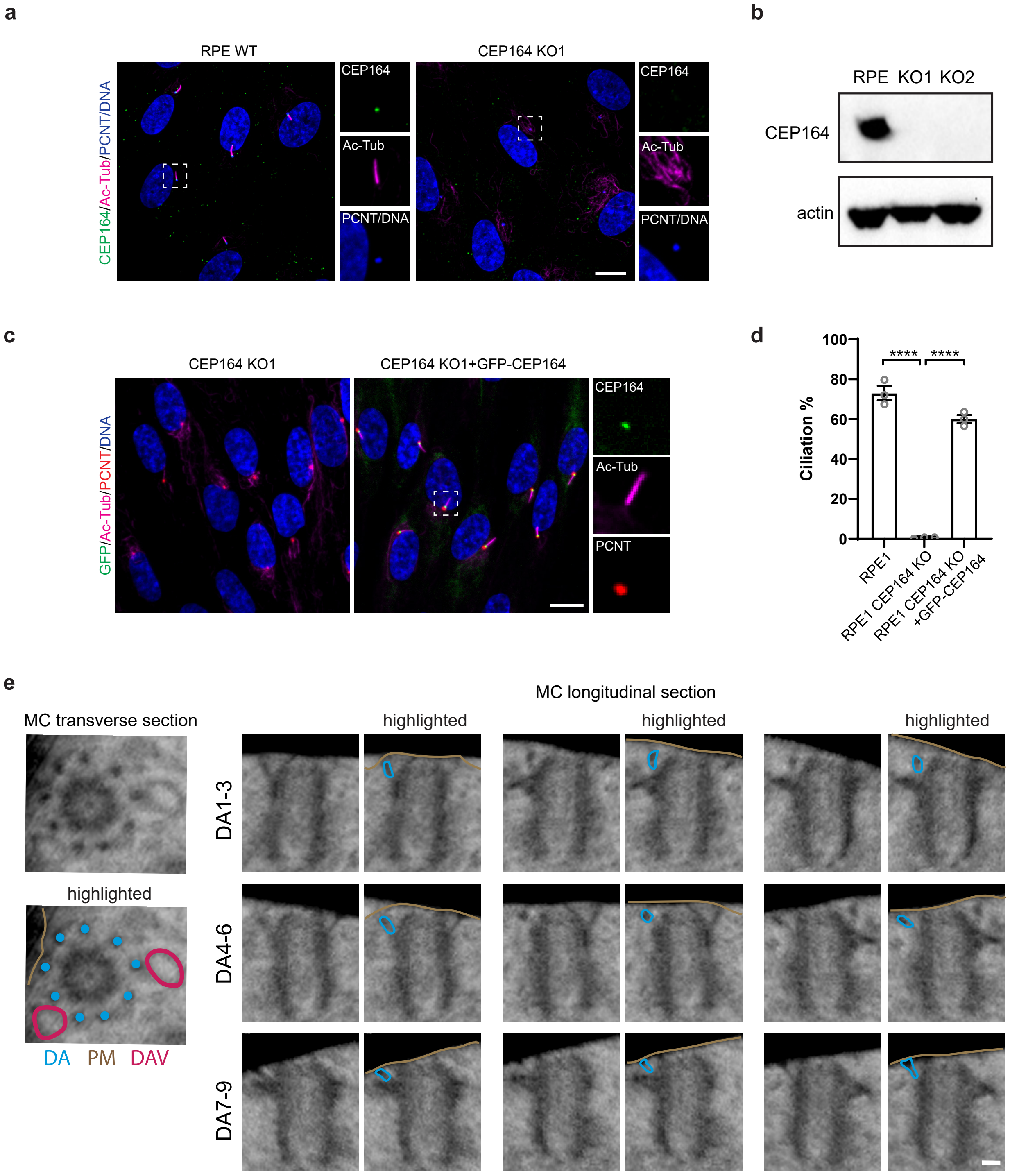

**Supplementary Fig. 3: CEP164 KO ciliogenesis analysis in RPE1 cells.**

**a** RPE1 wild-type and CEP164 KO cells were serum starved 24h and immunostained with CEP164, $^{Ac}$tub and pericentrin antibodies and the nuclear marker Hoechst. Scale bars 10 μm.**b** Immunoblot of cells described in **a** probed with CEP164 and actin antibodies.**c**, **d** GFP-CEP164 expression rescues ciliogenesis in CEP164 KO RPE1 cells. Cells were treated as described in **a** and cilia levels quantified. Means ± SEM (3 independent experiments, RPE1wild-type=462 cells, KO=380 cells, KO+GFP-CEP164=583 cells), Mean ± SEM, two tailed t-test, ***P<0.0001, Scale bars 10 μm.**e** Partial docking of MC to the PM in a CEP164 KO cell. FIB-SEM images of CEP164 depleted RPE1 GFP-CETN1 cell serum starved for 24h showing partial PM docking shown in Fig. 3a. DA 3 and 4 are not docked to the PM and DAVs are detected. Traced DAs (cyan), PM (brown) and DAVs (magenta) for corresponding FIB-SEM sections are shown. Additional FIB-SEM segmentations for CEO164 KO cells are shown in Supplementary Table 1. Scale bar 100 nm.

Supplementary Figure 4

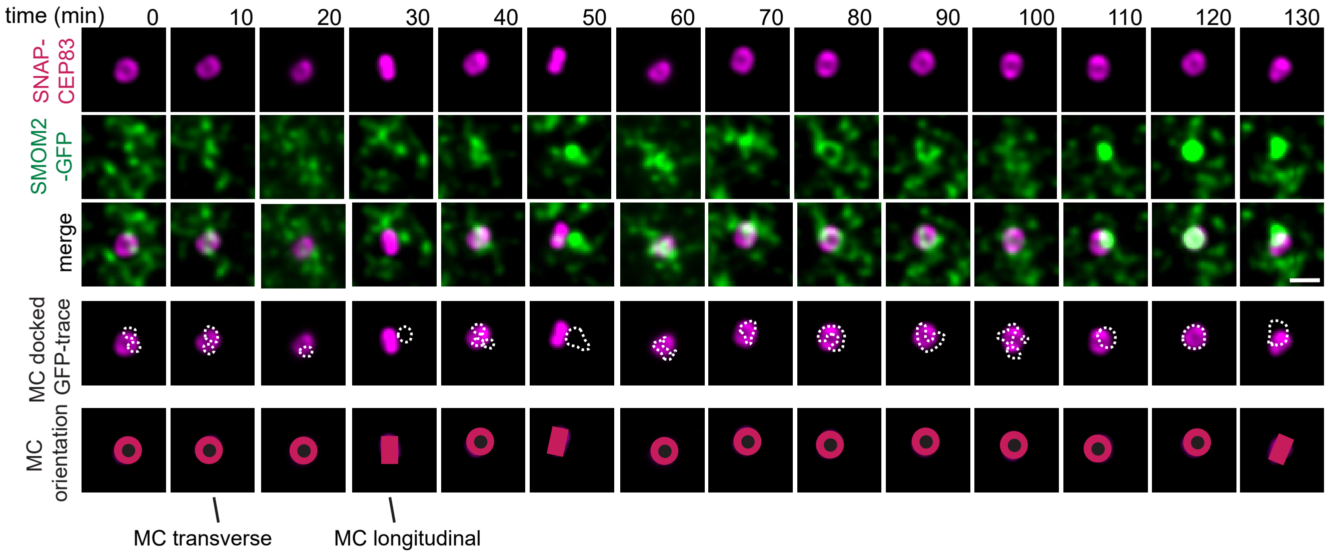


**Supplementary Fig. 4: SRM live cell imaging of ciliogenesis membrane assembly.**

$SIM^2$ super-resolution live-cell imaging of an RPE1 SMOM-GFP+SNAP-CEP83 cell stained and imaged as described in Fig. 4d following serum starvation. Bottom panels show the positional orientation of the MC DAs (magenta illustration) in the imaging plane (donut = MC transverse, line = MC longitudinal). White doted lines indicate the outline of SMO-GFP. Ring = MC facing up, solid line = MC sideways. Scale bar 1 µm.

Supplementary Figure 5

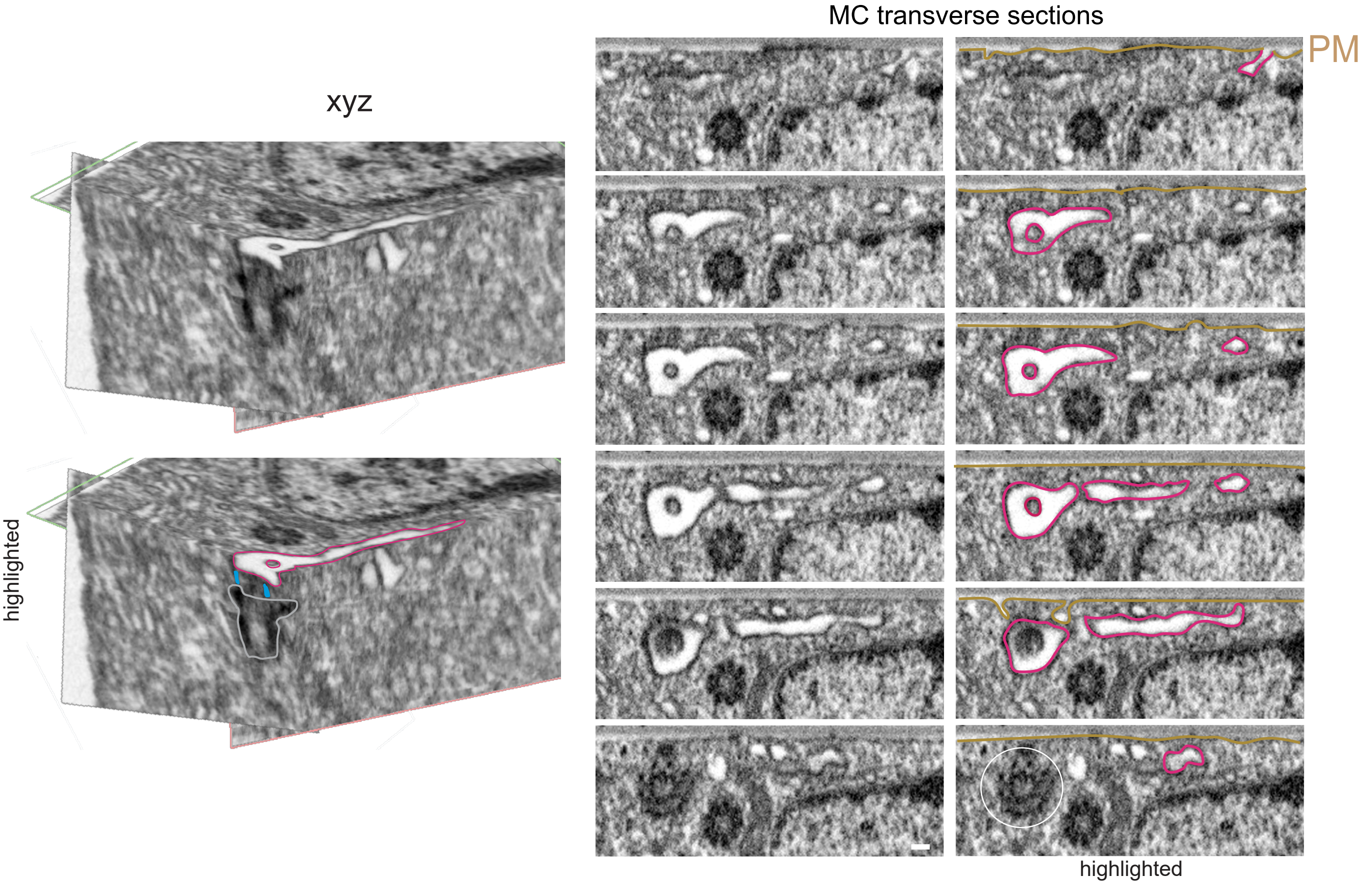


**Supplementary Fig. 5: FIB-SEM image slices of TCV.**

FIB-SEM images for the toroidal membrane shown and described in Fig. 4e. Left images show *xyz* planes through MC, toroid and EMC. Right images show additional transverse sections through MC and enlarged area showing the EMC connection to the PM. White circle shows MC position. Scale bar = 100 nm.

Supplementary Figure 6

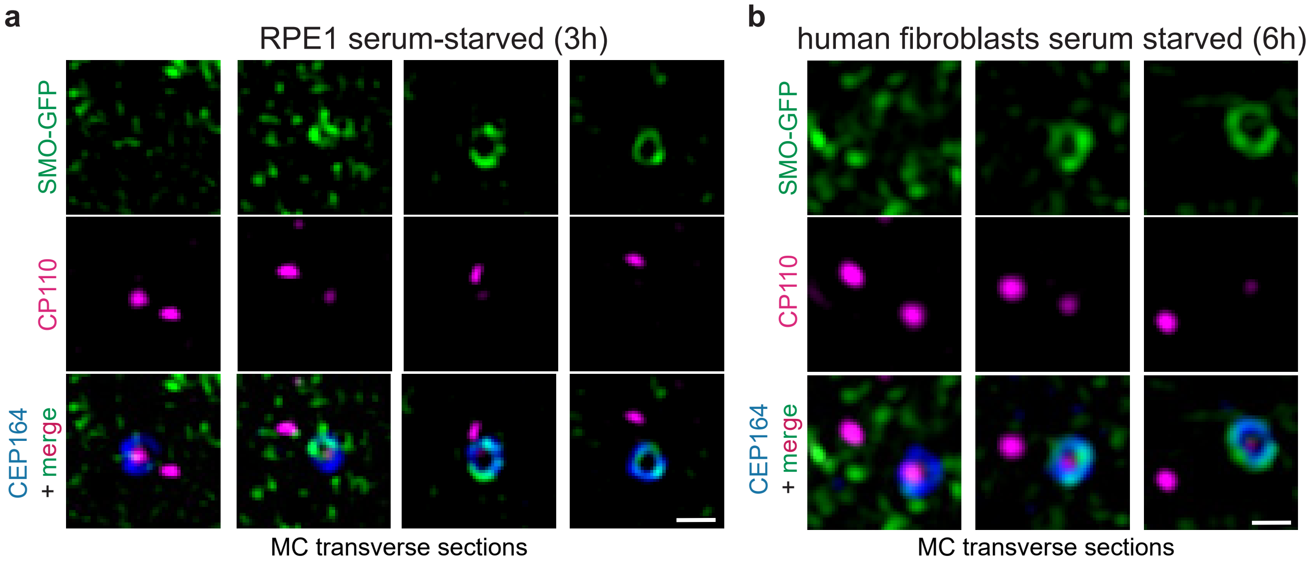


**Supplementary Fig. 6: MC uncapping association with ciliogenesis membrane accumulation.**

**a**, **b** Localization of SMO-GFP and CP110 on the MC in 3h serum-starved RPE1 (**a**) and 6h serum-starved human fibroblast (**b**) cells. Cells stably expressing SMO-GFP cells were fixed and stained with CP110 and CEP164 antibodies and imaged by Nikon SIM. Images show cells at different stages of ciliogenesis initiation marked by CP110 removal with associated SMO-GFP positive membrane docking. Cells were imaged with OMX SR imaging system. Scale bars 500 nm.

Supplementary Figure 7

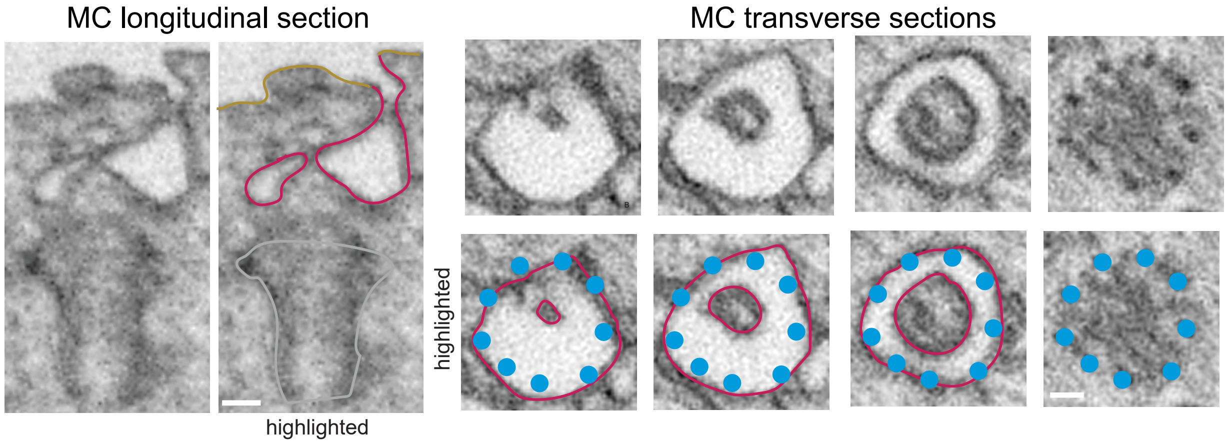


**Supplementary Fig. 7: FIB-SEM image slices of TCV.**

MC transverse (right images) and longitudinal (left) FIB-SEM images for the cell shown in the bottom panel of Fig. 5a with CP110 removed from the MC showing a TCV docked to the MC Transverse sections shows the presence of an EMC. PM highlighted in gold, TCV and EMC are highlighted in magenta and DA ends are marked with a cyan dot. Scale bars = 100 nm.

Supplementary Figure 8

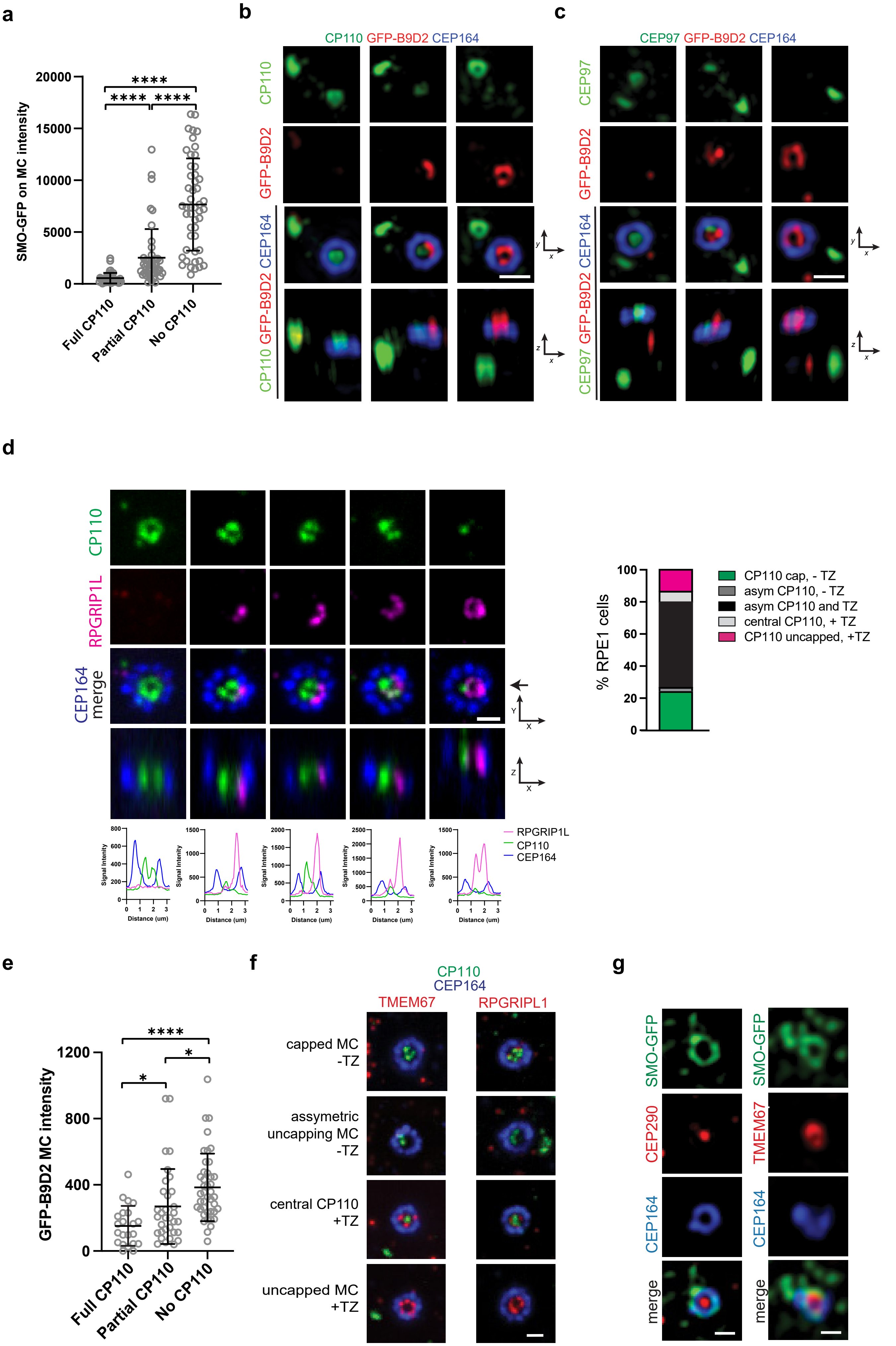

**Supplementary Fig. 8: MC uncapping association with TZ protein recruitment.**

**a** Quantification of SMO-GFP signal mean intensity associated with the MC in RPE1 cells serum-starved for 6h. Cells are subgrouped according to the signal intensity of CP110 at the MC. Data showing pooled images from 2 independent experiments. 43 cells with full CP110, 47 cells with partial CP110, and 49 cells with no CP110. Mean ± SD, two-tailed t-test, ****$p<0.0001$. **b**, **c** 3D-SIM images showing transition zone protein B9D2 recruitment to the MC in relation to MC uncapping during ciliogenesis. RPE1 cells stably expressing GFP-B9D2 were serum starved for 6h and stained with CEP164 and MC cap proteins CP110 (**a**) and CEP97 (**b**). Cartoons show fluorescence marker position on MC and DC associated with CP110 removal and TZ protein recruitment. Cells were imaged by OMX-SIM. Scale bars = 500 nm. **d** U-ExM images of CP110 and RPGRIP1L staining showing distribution of these proteins on the distal end of the MC. Representative RPE1 cells serum starved for 6h from two independent experiments showing progression of MC uncapping. Directional arrows indicate imaging plane. Top images show the projection of images across the MC distal end region. Bottom images show the orthogonal view along the Z-axis corresponding to the direction of the black arrow. Fluorescence intensity plot profiles correspond to the position of the black arrow. Right plot show the CP110 and RPGRIP1L localizations observed in the 43 cells imaged (Green: MC fully capped with CP110, no TZ; Gray: asymmetric localized CP110, no TZ; Black: asymmetric localized CP110 and TZ; White: asymmetric TZ and CP110 while CP110 localizes at the center of DA; Red: TZ while CP110 fully removed). Scale bar 1 µm. **e** Quantification of GFP-B9D2 signal intensity at the MC in RPE1 cells serum-starved for 3h as described and subgrouped as in **a**. Data showing pooled images from 2 independent experiments. 22 cells with full MC CP110, 33 cells with partial MC CP110, and 42 cells with no MC CP110. Mean ± SD, two-tailed t-test, ****$p<0.0001$, $p=0.03$ (Full CP110 vs Partial CP110), $p=0.023$ (Partial CP110 vs No CP110). **f** Representative images showing observed CP110 and TZ protein localization at the MC described in plots for Fig. 5d, e. Scale bar = 1 µm. **g** SIM images showing TZ protein MC localization in RPE1 cells with toroidal-like membranes. Cells expressing SMO-GFP were serum starved 6h and stained with TMEM67 or CEP290. SMO-GFP ring structures at the distal end of the MC. Scale bars = 500 nm.

Supplementary Figure 9

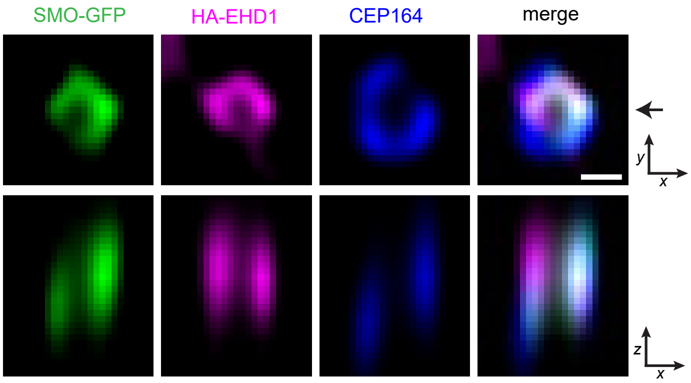


**Supplementary Fig. 9: HA-EHD1 localized to CCV-like structures with SMO-GFP in serum-fed cell.**

Serum-fed RPE1 GFP-SMO cell transiently expressing HA-EHD1 for 48h and stained with HA and CEP164 antibodies showing a C-shape like membrane structure. Directional arrows indicate imaging plane. Bottom images show the orthogonal view along the Z-axis corresponding to the direction of the black arrow. Sample was imaged with Zeiss Elyra SIM. Scale bar = 200 nm.

Supplementary Figure 10

**a**

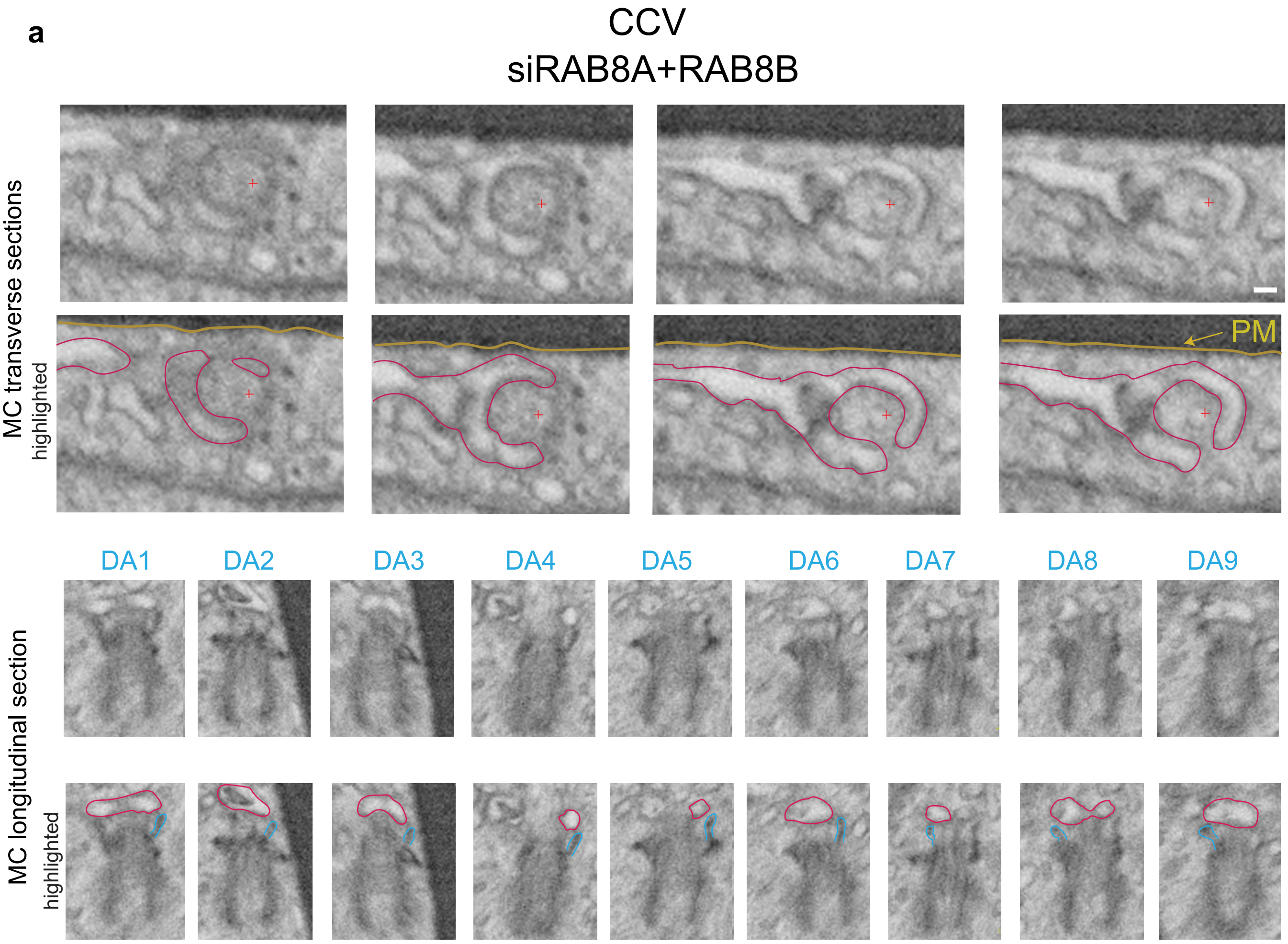


**b**

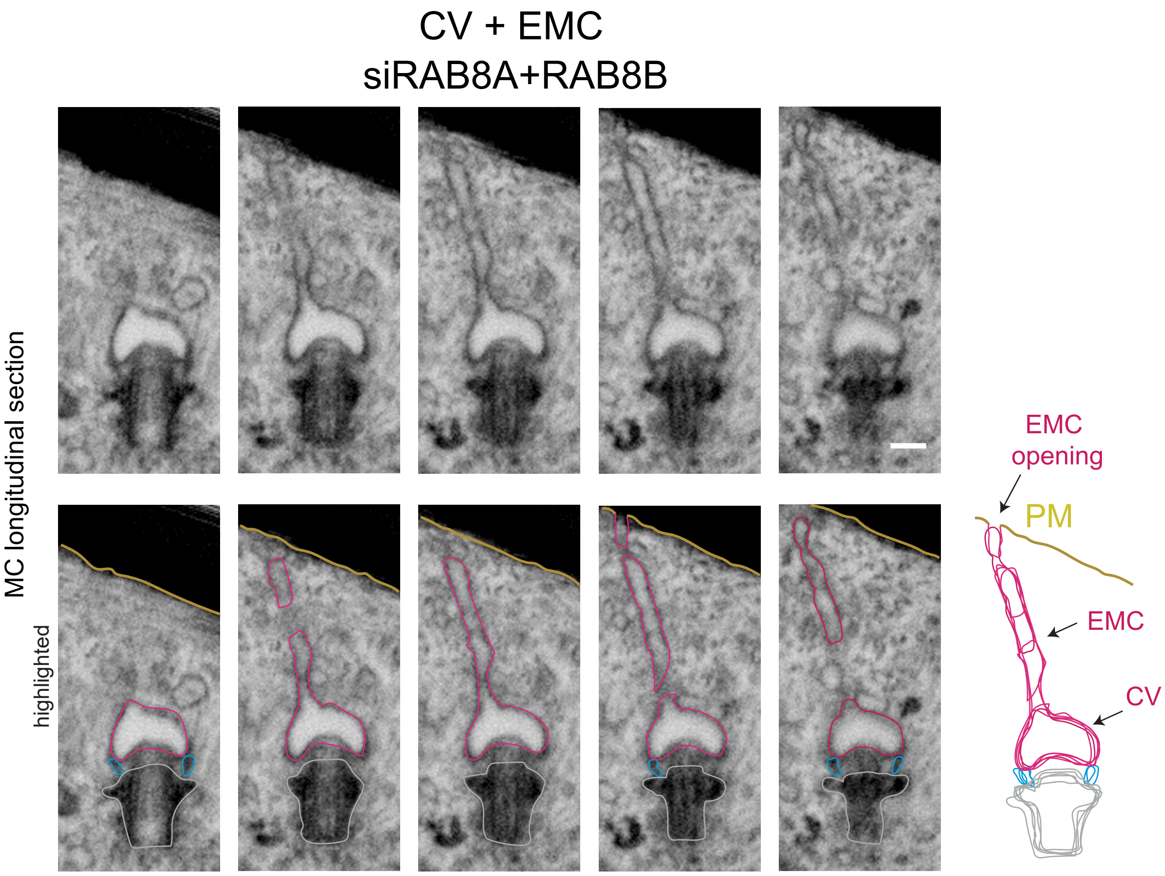

**Supplementary Fig. 10: FIB-SEM images of RAB8 depleted cell showing a CCV.**

**a** Transverse (top panels) and longitudinal (bottom panels) MC FIB-SEM images shown for cell described in Fig. 7c with CCV and extended membrane tubule. All 9 DA are shown in longitudinal sections (cyan trace) and CCV-membrane tubule (magenta). Plus (+) is a positional marker for section. Scale bar = 100 nm. **b** MC longitudinal FIB-SEM images shown for cell shown in Fig. 7e showing CV-EMC (magenta) connection to PM (gold trace). Scale bar = 200 nm.

Supplementary Figure 11

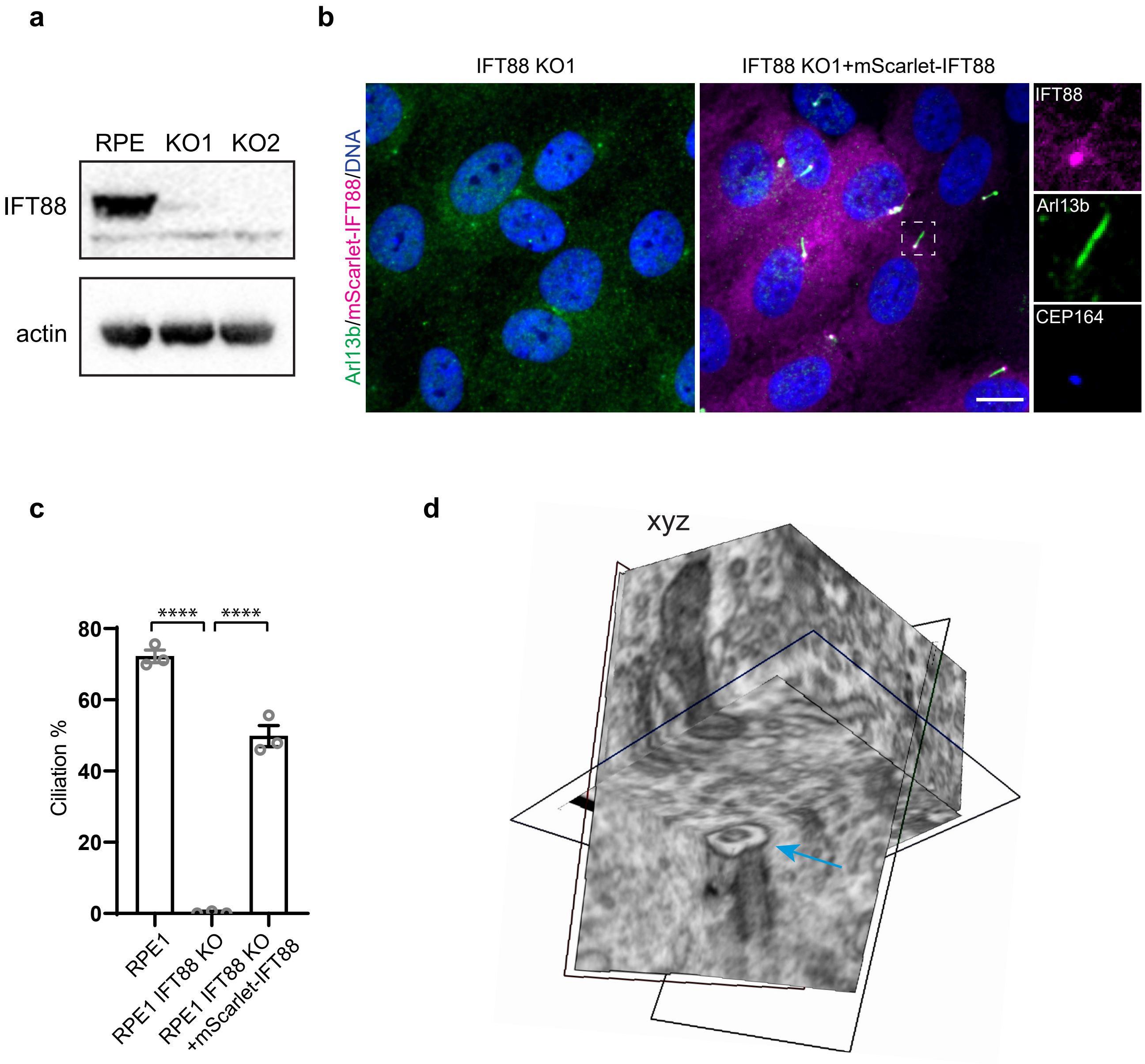


**Supplementary Fig. 11: RPE1 cell IFT88 KO ciliation analysis.**

**a** Immunoblot of IFT88 protein expression in wild-type RPE1 and IFT88 KO clonal cell lines (#1 and #2) using IFT88 and actin antibodies. **b**, **c** mScarlet-IFT88 expression rescues ciliogenesis in IFT88 KO RPE1 cells. Cells were serum starved 24h and immunostained with Arl13b (cilia) and CEP164 antibodies and ciliation quantified. Means ± SEM (3 independent experiments, RPE1=521 cells, KO=515 cells, KO+mScarlet-IFT88=490 cells). Mean ± SEM, two-tailed t-test, ****P<0.0001. Scale bar 10 µm. **d** FIB-SEM x,y,z planes of membrane toroid shown in Fig. 8d. Blue arrow shows MC structure.

SupplementaryTabel 1

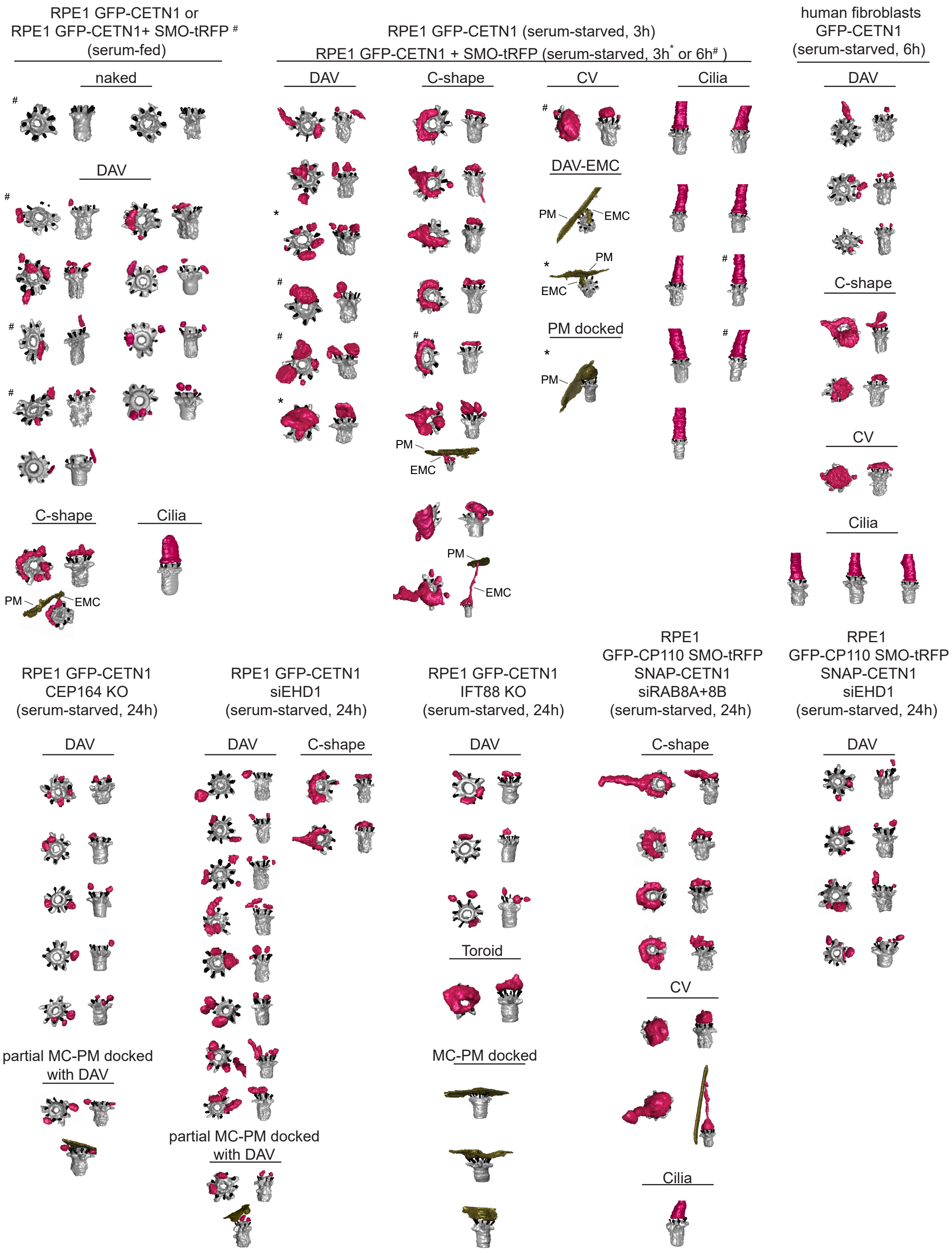


| cell line | serum treatement | n expt | total cells imaged | naked | DAV | CCV | TCV | CV | PM docked | partial PM docked | ciliary sheath/ cilia | DAV or CCV with EMC |
|---|---|---|---|---|---|---|---|---|---|---|---|---|
| RPE1 GFP-CETN1 | serum-fed | 2 | 9 | 1 | 6 | 1 | | | | | 1 | 1 |
| RPE1 GFP-CETN1+ SMO-tRFP | serum-fed | 1 | 4 | 1 | 3 | | | | | | | |
| RPE1 RPE1 GFP-CETN1 | starved 3h | 3 | 17 | | 2 | 7 | | | | 1 | 7 | 3 |
| RPE1 GFP-CETN1 + SMO-tRFP | starved 3h | 2 | 4 | | 2 | 0 | | | 1 | 1 | | |
| RPE1 GFP-CETN1 + SMO-tRFP | starved 6h | 2 | 6 | | 2 | 1 | | 1 | | | 2 | 1 |
| human fibroblasts GFP-CETN1 | starved 6h | 2 | 9 | | 3 | 2 | | 1 | | | 3 | |
| RPE1 GFP-CETN1 CEP164 KO | starved 24h | 2 | 6 | | 5 | | | | | 1 | | |
| RPE1 GFP-CETN1 siEHD1 (DAV and CCV) | starved 24h | 2 | 11 | | 9 | 2 | | | | | | |
| RPE1 GFP-CETN1 IFT88 KO | starved 24h | 1 | 7 | | 3 | | 1 | | 3 | | | |
| RPE1 GFP-CP110 Smo-tRFP SNAP-CETN1 siRAB8AB | starved 24h | 3 | 7 | | | 4 | | 2 | | | 1 | |

**Supplementary Table 1: FIB-SEM segmented MC structures.**

Segmented images of the MC from cells described in Fig. 1a, 2b, 2d-h, 2a, 2c-e, 3a-d, 3f, 5b, 7a, 8b and corresponding quantification of observed ciliary structures for conditions tested. Treatment conditions and experimental replicates (n) as indicated.

SupplementaryTabel 2

| ABBREVIATIONS | |
|---|---|
| **BB** | basal body |
| **CCV** | “C”-ciliary vesicle |
| **CLEM** | correlative light and electron microscopy |
| **CP** | ciliary pocket |
| **CV** | ciliary vesicle |
| **DA** | distal appendage |
| **DAP** | distal appendage protein |
| **DAV** | distal appendage vesicle |
| **EMC** | extracellular membrane channel |
| **FIB-SEM** | focused ion beam scanning electron microscopy |
| **GEF** | guanine nucleotide exchange factor |
| **MC** | mother centriole |
| **PCV** | preciliary vesicle |
| **PM** | plasma membrane |
| **proExM** | protein-retention expansion microscopy |
| **SD** | spinning disc |
| **SIM** | structured illumination microscopy |
| **SRM** | super-resolution light microscopy |
| **STED** | stimulated emission depletion |
| **TCV** | toroidal ciliary vesicle |
| **TZ** | transition zone |
| **U-ExM** | ultrastructure expansion microscopy |
| **vEM** | volume electron microscopy |

**Supplementary Table 2: Abbreviation table**
