# Supplementary material for: Characterization of membrane structures regulating primary ciliogenesis by quantitative isotropic ultrastructure imaging
Source: Nat Commun. 2026 Jun 13;17:7514. doi: 10.1038/s41467-026-73638-4 (PMC13408954; doi:10.1038/s41467-026-73638-4)
Supplement: Supplementary file 2 — Description of Additional Supplementary Files [file 41467_2026_73638_MOESM2_ESM.pdf]

Description of Additional Supplementary Files

Supplementary Movie 1. FIB-SEM image series with segmentation of a primary cilium from an RPE1 GFP-CETN1 cell.

3D longitudinal FIB-SEM and segmentation images of a RPE1 cilium shown in Fig. 1a. DA are highlighted in cyan, ciliary membrane in magenta and basal body in light gray. Cilium is rotated to show the association of each DA with ciliary membrane.

Supplementary Movie 2. FIB-SEM image series and segmentation of a CCV from a RPE1 GFP-CETN1 cell.

3D FIB-SEM and segmentation images of CCV from a RPE1 cell**.** DA are highlighted in black, CCV membrane in magenta and basal body in light gray. Video showing the raw image volume merged with segmented structures.

Supplementary Movie 3. FIB-SEM image series with segmentation of a CV structure from a human fibroblast GFP-CETN1 cell.

3D FIB-SEM and segmentation images of CV for the human fibroblast cell expressing GFP-CETN1 shown in Fig. 1d. DA are highlighted in black, CV membrane in magenta and basal body in light gray. Video showing the raw image volume merged with segmented structures.

Supplementary Movie 4. Segmentations of a cilia and a CCV showing membrane docking parameters at the DAs.

Segmented cilia from Supplementary Movie 1 (left) and CCV Supplementary Movie 3 (right) showing 3D analysis of membrane docking at the DA. 3D blue mesh spheres show 30 nm radius from the DA ends. DA are highlighted in black, CV membrane in magenta and basal body in light gray. Blue mesh spheres showing the space in the 30-nm range of each DA. Video showing segmented structures only.

Supplementary Movie 5. FIB-SEM image series and segmentation of DAV docking to the DAs.

3D FIB-SEM and segmentation showing analysis of DAV (magenta) and non-docked membranes (yellow) from RPE1 GFP-CENT1 cell. Analyzed DA are highlighted from black to cyan coloration and DAVs are identified within 30 nm of the DA ends (blue mesh sphere).

Supplementary Movie 6. FIB-SEM image series and segmentation showing DAV-EMC structures in a RPE1 GFP-CETN1 cell.

3D FIB-SEM and segmentation images of DAV-EMC containing MC shown in Fig. 2e. DAV-EMC structure is highlighted from gold to magenta coloration. PM associated membranes are colored in gold. DA are highlighted in black and basal body in light gray. Video showing raw image volume merged with segmented structures.

Supplementary Movie 7. FIB-SEM image series and segmentation view of CCV and DAV structures on the MC of a RPE1 GFP-CETN1 cell.

3D FIB-SEM and segmentation images of a MC containing a CCV and DAVs from a serum-fed RPE1 GFP-CENT1 cell**.** Blue mesh spheres show the 30 nm radius from the end of the DA. DA are highlighted in black and basal body in light gray. Video showing raw image volume merged with segmented structures.

Supplementary Movie 8. SRM SIM^2 live-cell imaging showing ciliogenesis progression in a RPE1 SMOM2-GFP and SNAPf-CEP83 cell.

SRM SIM^2 live cell time-lapse imaging showing ciliogenesis progression as in Fig. 4d. RPE1 cells expressing SMOM2-GFP and SNAPf-CEP83 were stained with SNAP-Cell 647-SiR dye and imaged every 10 min after serum-starvation. Images showing max-intensity projection of z-stacks around the basal body. Scale bar 500 nm.

Supplementary Movie 9. FIB-SEM image series and segmentation of TCV structure on the MC of a RPE1 GFP-EHD1 + SMO-tRFP cell.

3D longitudinal FIB-SEM and segmentation images of the TCV shown in Fig. 4e. DA are highlighted in cyan, basal body in light gray and TCV in magenta. Image volume is rotating to show each of the DA's association with the TCV membrane. Video showing raw image volume merged with segmented structures.
