# Supplementary material for: Characterization of membrane structures regulating primary ciliogenesis by quantitative isotropic ultrastructure imaging
Source: Nat Commun. 2026 Jun 13;17:7514. doi: 10.1038/s41467-026-73638-4 (PMC13408954; doi:10.1038/s41467-026-73638-4)
Supplement: Supplementary file 12 — Reporting Summary [file 41467_2026_73638_MOESM12_ESM.pdf]

nature portfolio

Corresponding author(s): Christopher Westlake

Last updated by author(s): Apr 7, 2026

Reporting Summary

Nature Portfolio wishes to improve the reproducibility of the work that we publish. This form provides structure for consistency and transparency in reporting. For further information on Nature Portfolio policies, see our Editorial Policies and the Editorial Policy Checklist.

Statistics

For all statistical analyses, confirm that the following items are present in the figure legend, table legend, main text, or Methods section.

n/a	Confirmed	
☐	☒	The exact sample size (*n*) for each experimental group/condition, given as a discrete number and unit of measurement
☐	☒	A statement on whether measurements were taken from distinct samples or whether the same sample was measured repeatedly
☐	☒	The statistical test(s) used AND whether they are one- or two-sided<br>*Only common tests should be described solely by name; describe more complex techniques in the Methods section.*
☒	☐	A description of all covariates tested
☒	☐	A description of any assumptions or corrections, such as tests of normality and adjustment for multiple comparisons
☐	☒	A full description of the statistical parameters including central tendency (e.g. means) or other basic estimates (e.g. regression coefficient) AND variation (e.g. standard deviation) or associated estimates of uncertainty (e.g. confidence intervals)
☐	☒	For null hypothesis testing, the test statistic (e.g. *F*, *t*, *r*) with confidence intervals, effect sizes, degrees of freedom and *P* value noted<br>*Give P values as exact values whenever suitable.*
☒	☐	For Bayesian analysis, information on the choice of priors and Markov chain Monte Carlo settings
☒	☐	For hierarchical and complex designs, identification of the appropriate level for tests and full reporting of outcomes
☒	☐	Estimates of effect sizes (e.g. Cohen's *d*, Pearson's *r*), indicating how they were calculated

Our web collection on statistics for biologists contains articles on many of the points above.

Software and code

Policy information about availability of computer code

Data collection	Zen balck 3.0, AcquireSR, softWoRx and NIS-Elements, MetaMorph Microscopy Automation and Image Analysis Software and 3i Slidebook are used to collect light microscopy data. ATLAS3D software is used for FIB-SEM data collection.
Data analysis	Zen balck 3.0, NIS-Elements, 3i Slidebook and Fiji are used for light microscopy data analysis. IMOD and Dragonfly softwares are used for FIB-SEM data analysis.

For manuscripts utilizing custom algorithms or software that are central to the research but not yet described in published literature, software must be made available to editors and reviewers. We strongly encourage code deposition in a community repository (e.g. GitHub). See the Nature Portfolio guidelines for submitting code & software for further information.

Data

Policy information about availability of data

All manuscripts must include a data availability statement. This statement should provide the following information, where applicable:

- Accession codes, unique identifiers, or web links for publicly available datasets
- A description of any restrictions on data availability
- For clinical datasets or third party data, please ensure that the statement adheres to our policy

The FIB-SEM datasets generated in this study have been deposited in the EMPIAR database under accession code EMPIAR-13463 (https://www.ebi.ac.uk/empiar/EMPIAR-13463). The raw mass spectrometry data have been deposited in the MassIVE database under accession code MSV000101315 [doi:10.25345/C5MK65P08]. Source data are provided with this paper.

Research involving human participants, their data, or biological material

Policy information about studies with human participants or human data. See also policy information about sex, gender (identity/presentation), and sexual orientation and race, ethnicity and racism.

Reporting on sex and gender	none
Reporting on race, ethnicity, or other socially relevant groupings	none
Population characteristics	none
Recruitment	none
Ethics oversight	none

Note that full information on the approval of the study protocol must also be provided in the manuscript.

Field-specific reporting

Please select the one below that is the best fit for your research. If you are not sure, read the appropriate sections before making your selection.

[x] Life sciences   [ ] Behavioural & social sciences   [ ] Ecological, evolutionary & environmental sciences

For a reference copy of the document with all sections, see nature.com/documents/nr-reporting-summary-flat.pdf

Life sciences study design

All studies must disclose on these points even when the disclosure is negative.

Sample size	For biochemistry and cellular experiments, all the experiments were repeated at least twice to ensure reproducibility. The number of cells quantified using light microscopy or FIB-SEM is indicated by n in the figure legends.
Data exclusions	no data was excluded
Replication	2 or more independent experiments were performed except for FIB-SEM analysis of 24 serum-starved RPE1 GFP-CETN1 IFT88KO and serum-fed RPE1 GFP-CETN1-CETN1+SMO-tRFP samples (n=1).
Randomization	For FIB-SEM data collection, random cell was selected to imaging in each view-field.
Blinding	Each FIB-SEM dataset was independently segmented by 3-5 people without prior knowledge of the structures.

Reporting for specific materials, systems and methods

We require information from authors about some types of materials, experimental systems and methods used in many studies. Here, indicate whether each material, system or method listed is relevant to your study. If you are not sure if a list item applies to your research, read the appropriate section before selecting a response.

Materials & experimental systems

n/a	Involved in the study	
[ ]	[x]	Antibodies
[ ]	[x]	Eukaryotic cell lines
[x]	[ ]	Palaeontology and archaeology
[x]	[ ]	Animals and other organisms
[x]	[ ]	Clinical data
[x]	[ ]	Dual use research of concern
[x]	[ ]	Plants

Methods

n/a	Involved in the study	
[x]	[ ]	ChIP-seq
[x]	[ ]	Flow cytometry
[x]	[ ]	MRI-based neuroimaging

Antibodies

Antibodies used	anti-Acetylated tubulin (Actub, clone 6-11B-1, 1/10000, T6793, Sigma), β-Actin−Peroxidase antibody (clone AC-15, 1/30000, A3854, Sigma), anti-EHD1 (EPR4954, 1/500, ab109311, Novus Biologicals), anti-RPGRIP1L (1/200, 55160-1-AP, Proteintech), anti-TMEM67 (1/200, 13975-1-AP, Proteintech), Rabbit anti-CEP164 (1/500, 22227-1-AP, Proteintech), chicken anti-CEP164 (Insinna, Lu et al. 2019), Rabbit anti-CP110 (1/1000, 12780-1-AP, Proteintech), mouse anti-CP110 (1/500, MABT1354, Millipore Sigma), anti-CEP97 (1/1000, A301-945A, Bethyl), anti-GFP Alexa 488 (1/1000, A21311, Molecular Probes Life Technologies), DAPI (1/2000, 62248, Thermo Scientific), Hoechst (1/3000, H3570, Molecular Probes Life Technologies) Rabbit anti-Pericentrin (1/2000, NB100-61071, Novus) and

	Mouse anti-Arl13b (1/500, N295B/66, NeuroMab) and all Alexa Fluor Dyes conjugated secondary antibodies were from Life Technologies. Goat anti-chicken IgY CF640R (Biotium, 20084), goat anti-rabbit CF568 (Biotium, 20099) and goat anti-mouse CF488 (Biotium, 20010)
Validation	All antibodies used here have been validated by commercial manufacture or by cited references. We have also confirmed the specificity of these antibodies by checking the molecular weight of proteins by WB, knock down of endogenous proteins or by immunostaning.

Eukaryotic cell lines

Policy information about cell lines and Sex and Gender in Research

Cell line source(s)	Human hTERT-RPE1 (CRL-4000) and 293T (CRL-3216) cell lines were obtained from ATCC. Human fibroblast cell line was obtained and cultured as previously described (Shimada, H et al 2017). Stable cell lines were generated using lentvirus infection or previously described. CEP164 and IFT88 KO cell lines were generated using CRISPR-Cas9 as described in methods.
Authentication	Cell lines used in this manuscript are authenticated by the manufacture.
Mycoplasma contamination	All cell lines were tested negative for mycoplasma contamination.
Commonly misidentified lines (See ICLAC register)	No commonly misidentified cells lines used in this study.

Plants

Seed stocks	N/A
Novel plant genotypes	N/A
Authentication	N/A
