# Supplementary material for: Characterization of membrane structures regulating primary ciliogenesis by quantitative isotropic ultrastructure imaging
Source: Nat Commun. 2026 Jun 13;17:7514. doi: 10.1038/s41467-026-73638-4 (PMC13408954; doi:10.1038/s41467-026-73638-4)
Supplement: Supplementary file 13 — Transparent Peer Review file [file 41467_2026_73638_MOESM13_ESM.pdf]

nature portfolio

Peer Review File

Characterization of membrane structures regulating primary ciliogenesis by quantitative isotropic ultrastructure imaging

Corresponding Author: Dr Christopher Westlake

This file contains all reviewer reports in order by version, followed by all author rebuttals in order by version.

Version 0:

Reviewer comments:

Reviewer #1

(Remarks to the Author)
In this manuscript, Lu et al. use advanced microscopy methods to present a detailed analysis of early stages in primary cilium assembly. Using cutting edge volumetric electron microscopy and super-resolution fluorescence microscopy methods, the authors dissect key intermediate steps in ciliary membrane formation and assign some known regulators of ciliogenesis to specific aspects of ciliary membrane biogenesis. These finding include several previously unreported structural intermediates (e.g. C-shaped and toroidal membrane assemblies on the mother centriole) and new functional roles (e.g. for CEP164 in growth of distal appendage vesicles and for asymmetric, progressive CP110 removal in allowing progressive growth of membrane intermediates and recruitment of transition zone proteins). In many cases, characterization of such details of ciliogenesis would not have been possible without the FIB-SEM, CLEM, and SIM methods employed, illustrating the value of applying these advanced imaging modalities to study cilium assembly and organelle biogenesis.

Among the limitations of the study, the findings reported are nearly all from RPE1 cells (which illustrate one pathway of cilium assembly), and how broadly these findings can be generalized to other cell types/tissues remains unclear. This issue warrants further discussion in the manuscript. Additionally, since the most informative ultra-structural studies come from static FIB-SEM snapshots after initiation of ciliogenesis, some of the claims regarding the sequence of events in ciliogenesis should be tempered to allow for the possibility that there are multiple parallel pathways and for the general difficulty in inferring temporal order from “static” data (additionally most data are from one timepoint after initiation of ciliogenesis). These caveats notwithstanding, the study is a technical tour de force in imaging and provides new levels of detail in understanding cilium formation. As such, the work will likely be of interest to the cilia and microscopy communities and is well suited to Nature Communications’s readership, provided the issues below can be addressed.

Main points

1. The presentation of the quantitative analysis of cells with different states of mother centriole-associated membranes is hard to follow. For Fig. 1b and Fig. 2d, these pie charts seem to describe the same set of data, but it is hard to follow some of the differences (e.g. ‘Other’ category in Fig. 1b was sub-divided into multiple sub-categories [‘Naked’ and ‘DAV’] in Fig. 2d; but at the same time the ‘cilia’ category from 1b was removed in 2d?). Supplementary Table 1 provides a helpful visualization of the different structures observed by FIB-SEM, but since it is not a traditional table is also difficult to parse. It would be helpful if numbers and fractions of cells in each group were also reported.

2. As noted above, the findings presented here are presented, to some extent, as representative of all modes of cilium assembly. While some of the vesicle docking and fusion events may commonly occur, it is not yet clear that these processes are always required for ciliogenesis across cell types and species.

3. Some aspects of the sequence of events are not definitively established given the limitations of EM analysis in fixed cells (and at a single timepoint). For example, the proposed progression from DAV to CCV to TCV to CV to cilium relies on few instances of CVs being observed. Can it be concluded with certainty that the CV is an obligatory intermediate step? Alternatively, if the authors propose that it is an obligatory step, is its rarity explained by the timepoint chosen for analysis (seemingly a suitable one since ~30% of cells have formed cilia while others are at early stages) or the possibility that the CV stage is a particularly short-lived intermediate? Likewise, in how many instances were TCV states observed (it would be

helpful if these were included in the summary presentation in Supplementary Table 1)? Given the rarity of TCV and CV observations, additional supporting evidence/explanations or tempered conclusions might be warranted.

4. It is surprising that so many RPE1 cells in serum-fed conditions have DAVs. Can these DAV stages be observed by any fluorescence microscopy markers? Did the authors make similar observations in other cell types and/or at low cell densities? It is likewise surprising that CEP164 is not needed for DAV docking. Is this true for other DAPs (e.g. DAPs such as CEP83 that lie at a more fundamental position in hierarchy of distal appendage assembly)? Loss of these DAV structures in a full distal appendage knockout background would help confirm that the structures identified as DAVs in FIB-SEM are in fact bona fide DAVs. Lastly, given the prevalence of DAVs under non-ciliating conditions, is the statement "Together these results confirm that DAV docking at the MC is associated with ciliogenesis progression" (lines 169-170) accurate?

Other points

1. Can TZ recruitment relative to other steps be clarified/illustrated in the model shown in Fig. 8e?

2. Fig 4d: Why does the apparent SmoM2 diameter decrease significantly after the CCV stage? Is this expected for e.g. a CV stage?

3. The analysis of EHD1 over-expression and its impact on CP110 removal in serum-fed cells is interesting. How does GFP-EHD1 over-expression impact initial stages of ciliogenesis in serum-fed cells (apart from CP110 removal) without leading to a significant increase in cilia formation? Can SRM imaging reveal if these cells form CCV or toroidal or CV structures (or are arrested at a specific stage in this progression)?

4. The images in Supplementary Table 1 for EHD1 RNAi in a wildtype RPE1 background look different than for EHD1 RNAi in the background with fluorescent markers expressed. Specifically, the former show relatively numerous/large DAVs and some CCVs, whereas the latter showing smaller and fewer DAVs in a manner more reminiscent of the CEP164 KO cells. Can the authors clarify these apparent differences and which EHD1 RNAi FIB-SEM data are described in the Results?

5. Lines 363-365. "These observations support requirements for tubular membrane assembly in instructing MC uncapping and indicate that RAB8-dependent CCV "C" gap closure upstream of TCV assembly is important for completing CP110 removal" Is it not equally or more likely that CP110 removal enables RAB8-dependent "C" gap closure and TCV formation rather than vice versa?

6. It seems possible that TZ protein recruitment versus TZ assembly (e.g. into Y-linked architecture) are distinct steps, with the former occurring at the CCV stage, while the second may occur later and be subject to additional regulation and prerequisite steps in cilium formation. It might be helpful to discuss this point in the context of the authors' observations.

7. It is surprising that IFT88 KO leads to accumulation of cells with the MC docked to the PM, a stage that is not typically seen in the sequence of events associated with ciliogenesis in wildtype cells. Can the authors clarify how they feel this observation fits with their model and the step(s) at which IFT88 may act?

8. What exactly is shown in Supp. Fig 6? A series of different cells imaged by SRM (or different z planes or time points from one cell)?

9. Lines 389-390: "dynamics in the low range nanoscale range". Typo

10. Lines 435-437: "What is clear is that TZ protein accumulation at the MC coincides with CCV and TCV assemble suggestive that the TZ is being organized." This wording is confusing.

Reviewer #2

(Remarks to the Author)
In the article "Characterization of membrane structures regulating primary ciliogenesis by quantitative isotropic ultrastructure imaging", Lu and colleagues investigate the early steps of ciliary vesicle assembly during ciliogenesis. They show, for the first time, that the enlargement and fusion of small vesicles at the mother centriole (MC) into novel tubular C-shaped and toroidal membrane structures is a critical step in primary cilium formation. The authors demonstrate the importance of these membrane intermediates in recruiting and stabilizing transition zone complexes, as well as in the removal of the capping complex CP110/CEP97—both of which are essential for ciliogenesis.
Furthermore, they describe the involvement of molecular regulators such as EHD1 and RAB8 in orchestrating the membrane trafficking and fusion events that lead to the formation of these intermediates. This study highlights the power of 3D isotropic ultrastructure imaging in uncovering dynamic membrane remodeling processes during organelle biogenesis. The combination of vEM-FIB-SEM with correlative light and electron microscopy (CLEM), and photonic super-resolution microscopy—including live and fixed SIM, STED, and expansion microscopy—constitutes an impressive tour de force.

While I acknowledge the tremendous amount of work, the high quality of the data, and the novel biological insights presented, I would like to raise several crucial points that need to be addressed before this article can be considered for publication.
Major points:
1) Throughout the paper, the authors imply a notion of temporality in the maturation of ciliary vesicles. This is an important

point of the paper that is not well supported by the data. Indeed, only one time point of ciliogenesis is used. Although the authors performed experiments at 3h and 6h post-serum starvation, they pooled the results from both time points, making the temporality impossible to establish. They based their conclusion on rare events (sometimes only one cell) observed in non-starved conditions to conclude on the temporality (Figure 1 and 2). The authors should consider: i) increasing the number of events in the "non-starved condition"; ii) adding intermediate time points during ciliogenesis (e.g., 3h versus 6h); or iii) removing / down tuning their interpretation regarding temporality

2) Quantifications are missing in several key experiments to support the conclusions:
• Additional graphs showing the quantification regarding the intermediate stage of vesicle formation would be welcome, notably for the tubulogenic extension from the CCV phase and the EMC events, which seem to be quantifiable according to the main text, but no apparent quantification appears in Figure 1.
• Quantification of the behavior of GFP-SMO over time (from dots to C-shape to donut) in relation to the appearance of the cilium would be appreciated to give a temporal dimension to ciliary vesicle maturation.
• Quantification of the correlation between CP110 removal and SMO-vesicle accumulation, as well as TZ accumulation, is necessary to support the conclusion regarding CP110 regulation.
• All quantifications represented as 'pie charts' need to be revised and presented as graphs that allow for visualization of i) experimental reproducibility; ii) percentage; and iii) number of total events quantified.
3) The use of 'pro-ExM' to study centrioles is questionable here. As demonstrated in Gambarotto et al., 2019 (https://doi.org/10.1038/s41592-018-0238-1), the 'pro-ExM' protocol is not suitable for isotropic expansion of centrioles. Pre-expansion staining of such a compact structure leads to limited epitope accessibility, and sample fixation triggers non-isotropic expansion of this large macromolecular complex. For these reasons, interpretation of asymmetric CP110 staining is questionable, as it could be due to poor expansion. I therefore strongly recommend using Ultrastructure Expansion Microscopy as described in this paper (https://doi.org/10.15252/embj.2022112107), which uses U-ExM to study centriolar architecture in RPE1 cells, including similar stainings (CEP164 and CP110). Moreover, expansion validation (expansion factor) is missing.
4) A thorough description of the culture conditions—particularly medium composition and seeding density for the ciliogenesis experiments—is missing. Given the variety of protocols used to study ciliogenesis and the variability in results in the literature, providing this information is essential.
Minor points:
A. General comments:
1. I recommend a careful read of the manuscript to check for spelling and typos, particularly in the figure legends.
2. The definitions of PCV, CV, CCV, EMC, etc., should be listed in a table or in an inset present in all figures where these abbreviations are used. In Figure 1, for instance, the definition of CCV appears too late in the description (only when mentioning Figure 1g in the main text), while the abbreviation is already used in the quantification of Figure 1d, making reading difficult.
3. The use of the dash symbol (–) is not consistent across the manuscript. Sometimes it indicates the presence of serum (e.g., Figure 1b legend: "Quantification of ciliary structures identified by FIB-SEM in serum-fed RPE1 cells") and elsewhere the absence (e.g., Figure 1h and 1i: "CCV, human fibroblast – serum"). For consistency, the symbol should not be used to indicate serum absence, which should be referred to as "starved condition."
4. The current names of the supplementary videos are unclear and do not allow easy identification of their correspondence to the text. Additionally, a legend for each video would be welcome (especially for Movie S8).
B. Results interpretation:
1. In Figure 3a, CEP164 KO cells show DAV docking, likely due to the presence of intact DAs. The proportion of cells still containing DAs should be quantified to support the presence of DAV.
2. Figure 4a – The conditions are unclear. Are these images from cells in the presence or absence of serum? A sign (+/–) is missing in the right panel. It is also surprising to use a non-starved (+ serum) image to highlight CCV presence, which (according to Figure 1) is observed in only one cell in serum. Could the authors clarify whether the 'CCV + DAV' situation represents an intermediate stage in serum-fed cells? If not, they should use starved cells for this observation for consistency.
3. The use of SMO-GFP/SMO-tRFP (Figures 3g, 4e, Supp6) versus GFP-SMOM2 (Figure 4d) is puzzling. How could the authors visualize the pre-ciliary membrane with regular SMO in Figures 3a/4e but require a specific SMO variant in Figure 4d? This difference should be clarified.
4. Figure 4d – It's unclear whether the same centriole/cilium is followed over time. The change in orientation from transverse to longitudinal view at t:70, 80, and 90 is confusing and may suggest two different samples. If the same sample is followed from t:0 to t:90, the change in orientation should be clearly noted.
5. Figures 5d, e – The interpretation of CP110 staining evolution exceeds the presented results. Adding a temporal element or correlating with ciliary growth would help evaluate CP110 removal more accurately.
6. Figures 6c, d – The text states: "Immunoblot analysis of coexpression (CO)-IP studies confirmed GFP-CP110 and GFP-CEP97 can interact with HA-tagged EHD1, but not with the other ciliogenesis membrane trafficking regulators tested (Fig. 6c, d)," yet the figures show GFP-CP110 also interacts weakly with PASCIN1, and GFP-CEP97 shows interaction with RAB8 and RABIN8. This discrepancy should be addressed.
7. Figure 5e – The experimental design is unclear. Are cells co-transfected with GFP-CP110 and GFP-CEP97 at various plasmid amounts? The experimental description and figure legend need clarification.
C. Figure typos and others:
1. Figure 2a – The green numbers (distances) are hard to read and should be either recolored or numbered and referenced in the legend.
2. Figure 2a i) and ii) – Color codes should match across both panels (currently DA is blue in i) and orange in ii)).
3. Supplementary Figure S3a – The "AcTub" channel is magenta, but the legend indicates red. Also, a scale bar is missing.
4. Figure 4c – The largest angle mentioned in the text (149°) does not match the one shown in the figure (153°).
5. Figure 4d (and Supp. 4) – Annotations would help visualize vesicular features. The bottom panel of 4d is not explained

well. The legend (“MC transverse and longitudinal images...”) doesn’t clarify its purpose.
6. Figure 5a – The left-hand scheme indicating SMO-tRFP position does not match the SIM image. The bottom panel shows a ring-shaped vesicle, while the image depicts a C-shaped one. This should be revised.
7. Figure S8a – The centriole schematic appears misoriented compared to the microscopy image.
8. Figures 6c, d – The phrase “coexpression (CO)-IP” should be replaced with “co-immunoprecipitation.”
9. In the sentence: “To examine the ciliogenic potential of DAVs, we examined the distribution and organization of these membranes in RPE1 cells grown under non-ciliating and ciliating conditions and in starved cells lacking CEP164,” the conditions are unclear. Is this a repetition, or are “ciliating condition” and “starved cells” different?

Reviewer #3

(Remarks to the Author)
In this manuscript Lu and colleagues investigate the early stages of primary cilium formation, focusing on the 3D membrane ultrastructure. Utilizing FIB-SEM and super-resolution microscopy, the authors identify two unknown membrane intermediates that are formed prior to the well-established ciliary vesicle (CV): the tubular C-ciliary vesicle (CCV) and the toroidal ciliary vesicle (TCV). Furthermore, the authors analyze the functions of CEP164, EHD1, RAB8 and IFT88 in regulating the formation of these membrane intermediates. Key findings include the requirement for EHD1 and RAB8 in CCV/TCV formation, a novel requirement for CEP164 in vesicle expansion, and confirmation of IFT88's role in the initiation of ciliogenesis. This study proposes a revised architectural framework for early ciliogenesis, emphasizing the tubular membrane intermediates, and highlights the power of isotropic ultrastructure imaging and quantitative 3D analysis. I would support the publication of the manuscript in Nature Communications once the following concerns are addressed.

Specific comments for the authors:

RPE1 cells are typically understood to employ the intracellular pathway for ciliogenesis. However, the data presented includes observations of mother centriole docking directly to the plasma membrane, a characteristic of the extracellular pathway. Could the authors discuss these observations in the context of the known RPE1 ciliogenesis mechanism? Does this suggest that RPE1 cells might utilize both pathways, or are these observations indicative of a specific cellular state or context that influences the chosen pathway?

Figure 2 shows instances where Distal Appendage Vesicles (DAVs) appear to mediate the docking of the mother centriole to the plasma membrane. This observation regarding the role of DAVs in docking to the plasma membrane is not explicitly included or reconciled within the proposed working model presented in Figure 8e, which focuses primarily on the intracellular pathway intermediates (CCV, TCV, CV). Could the authors clarify if the docking of mother centriole to the plasma membrane mediated by early ciliogenesis vesicles is a common observation? If so, they need to integrate this step with their model in figure 8.

In Line 164, the authors report that 3/4 of RPE1 cells grown under serum-fed non-ciliating conditions display DAVs or CCVs. However, earlier electron microscopy studies often reported few or no discernible vesicles directly associated with the mother centriole in cycling cells. Could the authors discuss the discrepancy? Whether their cell culture conditions (e.g., cell density) might influence the prevalence or visibility of these early membrane intermediates compared to what has been observed in other studies?

The authors define CCVs as vesicles that cover more than 50% of the circumference of the DA region. In Line 130, they state that CCVs are present in approximately one-third of the cells. However, this raises the question that what occurs in the remaining two-thirds of the cells? The authors ought to present (quantitative) data on their observations of these cells. Also, there is a likelihood that not every cell forms a single CCV; instead, some cells may form two or more vesicles that subsequently fuse to create a TCV or CV. Live-cell super-resolution imaging (quantitatively) could be an appropriate method to either confirm or refute this possibility.

Figure 3g depicts the co-localization of EHD1 and smoothened. Nevertheless, when observed from a longitudinal perspective, it appears that these two proteins do not exhibit strong co-localization. The authors need to verify whether this is indeed the actual situation or if it is a result of misalignment of the two channels during the data acquisition process.

In Figure 3b, c, and d, the second bars lack annotations. To prevent confusion, the authors should label these bars as "no treatment". Also, some quantitative analyses are missing: b) no treatment vs siEHD1, no treatment vs CEP164 KO; c) serum vs no treatment, no treatment vs siEHD1; d) no treatment vs siEHD1; siEHD1 vs CCV. Same with Figure 6h.

The authors state that the EHD1 K483E mutant does not associate with tubular membranes, fails to rescue CP110 removal in EHD1-depleted cells, and shows negligible binding to GFP-CP110 compared to the wild-type protein. The above results do not support the claim in Line 321 that EHD1 membrane tubulation function is essential for interaction with CP110. There is an alternative scenario that the K483E mutation could independently impact EHD1’s association with both the tubular membrane and CP110. Similarly, the results do not support the conclusion drawn in Line 334, which posits that EHD1 on tubular CCV membranes directs MC uncapping via biochemical association with CP110 and CEPE97.The authors should either provide additional evidence or rephrase these conclusions to more accurately reflect the findings of their study.

Version 1:

Reviewer comments:

Reviewer #1

(Remarks to the Author)
In their revised manuscript, Lu et al. present updated findings on the process of ciliary membrane formation during ciliogenesis. They have helpfully improved their explanation and quantification of the data collected across different experiments, timepoints, and cell lines. These and other revisions have largely addressed the questions raised regarding the initially submitted manuscript. Collectively, this study showcases the power of new volumetric EM methods for high-resolution, quantitative analysis of complex and dynamic cellular processes. It is a technical tour de force and offers valuable new insights into ciliogenesis. I therefore recommend publication in Nature Communications and only highlight below a few minor points that the authors should ideally address as they finalize their manuscript.

1. Clearly the role of EHD1 in ciliogenesis is complex and multi-factorial, given the reported effects of EHD1 knockdown on CP110 removal and on EMC formation. Regarding the section starting at line 243 “EHD1 functions in fusion of enlarged DAVs into the CCV”, how compelling is the evidence that EHD1 RNAi impairs CCV formation? Figure 3 does illustrate that DAV membrane growth is unimpaired in EHD1-depleted cells (unlike in CEP164 KO cells), but it does not seem definitive that CCV formation is reduced. It is true that only two of eleven cells under this condition have CCV’s at 24 h, but is this significantly different than the 8/27 wildtype RPE1 cells with CCVs (combining the WT data in Table S1)? Is the size / radial span of the ‘C’ shaped CCV’s reduced in the EHD1 RNAi cells? While a role for EHD1 in CCV formation is certainly plausible, it may be helpful to clarify the evidence or temper the strength of the conclusions.

2. The presentation and interpretation of the RAB8 RNAi results remains somewhat confusing. The section title “RAB8 functions upstream of the membrane toroid structures in ciliogenesis” implies that TCV structures and downstream CV structures would be unable to form in RAB8 knockdown cells, but the quantification in Table S1 does not clearly match this prediction (e.g. CV’s can form; and while no TCV was observed, only one TCV was observed across the entire study, so its absence is not necessarily compelling). I think the clearest effect is that DAV intermediates are not seen at 24 hr, indicating that RAB8 is dispensable for the DAV->CCV transition. Whether RAB8 promotes TCV formation or CV formation or CV growth into a cilium does not seem entirely clear, and thus it may be helpful to temper statements regarding the proposed role in CCV closure to form the TCV. Also, the sentence “Strikingly, our concerns about the use of TEM for precisely defining ciliogenesis mechanisms were further confirmed” (lines 401-402) is not explained.

3. I suggest some further review/editing for grammar and clarity. In the case of lines 320-321, please revise to clarify. I am also unsure what is meant on lines 385-386 regarding the effect of EHD1 over-expression: it seems that one explanation could be that in some cases ciliogenesis progression is limited independently of EHD1-faciliated removal of CP110 – is that the point that is intended here? Lastly, while a number of the reported observations are indeed compelling, the frequent use of ‘strikingly’ may be a bit distracting to the reader.

Reviewer #2

(Remarks to the Author)
In the article “Characterization of membrane structures regulating primary ciliogenesis by quantitative isotropic ultrastructure imaging”, Lu and colleagues investigate the early steps of ciliary vesicle assembly during ciliogenesis. They show for the first time that the enlargement and fusion of small vesicles at the mother centriole (MC) into novel tubular, C-shaped, and toroidal membrane structures represent a critical step in primary cilium formation. The authors further demonstrate the importance of these membrane intermediates in recruiting and stabilizing transition zone complexes, as well as in the removal of the capping complex CP110/CEP97—both processes being essential for ciliogenesis.

In this study, the authors combine several state-of-the-art imaging approaches, including volumetric FIB-SEM, structured illumination microscopy (SIM), and expansion microscopy. This innovative work sheds light on previously undescribed phenomena occurring during the early stages of ciliogenesis.

In the revised version, the authors have made a tremendous effort to address the main weaknesses of the original manuscript. I particularly appreciate the additional quantifications and immunolabeling performed for the immunofluorescence experiments, as well as the increased number of samples and replicates, despite the considerable technical challenges faced during the revision process.

Overall, I am convinced that this study will be highly insightful for the community and is fully appropriate for publication in Nature Communications.

Reviewer #3

(Remarks to the Author)
The authors have satisfactorily addressed all my concerns. I recommend the manuscript for publication.

Version 2:

Reviewer comments:

Reviewer #1

(Remarks to the Author)
In their updated manuscript, Lu et al. have addressed all of my concerns. I appreciate their thorough responses and extra effort, and I strongly support publication of their study in Nature Communications. It is a technical tour de force that provides a newly detailed, comprehensive portrait of ciliogenesis that will be an important contribution for a broad audience.

Open Access This Peer Review File is licensed under a Creative Commons Attribution 4.0 International License, which permits use, sharing, adaptation, distribution and reproduction in any medium or format, as long as you give appropriate credit to the original author(s) and the source, provide a link to the Creative Commons license, and indicate if changes were made.
In cases where reviewers are anonymous, credit should be given to 'Anonymous Referee' and the source.
The images or other third party material in this Peer Review File are included in the article's Creative Commons license, unless indicated otherwise in a credit line to the material. If material is not included in the article's Creative Commons license and your intended use is not permitted by statutory regulation or exceeds the permitted use, you will need to obtain permission directly from the copyright holder.
To view a copy of this license, visit https://creativecommons.org/licenses/by/4.0/

We thank the reviewers for their constructive feedback and for the time invested in evaluating our work. In response, we have substantially revised the manuscript and added new data to strengthen our findings. We have provided two versions of the revised manuscript: one with all changes highlighted in red and a clean version without marks. Below, we provide a point-by-point response: reviewers' comments are in blue; our responses are in black; and quoted manuscript revisions are in red.

Reviewer #1

In this manuscript, Lu et al. use advanced microscopy methods to present a detailed analysis of early stages in primary cilium assembly. Using cutting edge volumetric electron microscopy and super-resolution fluorescence microscopy methods, the authors dissect key intermediate steps in ciliary membrane formation and assign some known regulators of ciliogenesis to specific aspects of ciliary membrane biogenesis. These finding include several previously unreported structural intermediates (e.g. C-shaped and toroidal membrane assemblies on the mother centriole) and new functional roles (e.g. for CEP164 in growth of distal appendage vesicles and for asymmetric, progressive CP110 removal in allowing progressive growth of membrane intermediates and recruitment of transition zone proteins). In many cases, characterization of such details of ciliogenesis would not have been possible without the FIB-SEM, CLEM, and SIM methods employed, illustrating the value of applying these advanced imaging modalities to study cilium assembly and organelle biogenesis.

Among the limitations of the study, the findings reported are nearly all from RPE1 cells (which illustrate one pathway of cilium assembly), and how broadly these findings can be generalized to other cell types/tissues remains unclear. This issue warrants further discussion in the manuscript. Additionally, since the most informative ultra-structural studies come from static FIB-SEM snapshots after initiation of ciliogenesis, some of the claims regarding the sequence of events in ciliogenesis should be tempered to allow for the possibility that there are multiple parallel pathways and for the general difficulty in inferring temporal order from "static" data (additionally most data are from one timepoint after initiation of ciliogenesis). These caveats notwithstanding, the study is a technical tour de force in imaging and provides new levels of detail in understanding cilium formation. As such, the work will likely be of interest to the cilia and microscopy communities and is well suited to Nature Communications's readership, provided the issues below can be addressed.

We thank the reviewer for these encouraging and constructive comments.

Main points

1. The presentation of the quantitative analysis of cells with different states of mother centriole-associated membranes is hard to follow. For Fig. 1b and Fig. 2d, these pie charts seem to describe the same set of data, but it is hard to follow some of the differences (e.g. 'Other' category in Fig. 1b was sub-divided into multiple sub-categories ['Naked' and 'DAV'] in Fig. 2d; but at the same time the 'cilia' category from 1b was removed in 2d?). Supplementary Table 1 provides a helpful visualization of the different structures observed by FIB-SEM, but since it is not a traditional table

is also difficult to parse. It would be helpful if numbers and fractions of cells in each group were also reported.

Response Reviewer #1 Main Point #1:

Thank you for highlighting the need for clearer quantification, which was also raised by Reviewer #2. We have added a numerical summary of the FIB-SEM observations in Supplementary Table 1 and removed the pie charts, as requested by reviewer#2. Fig. 2d now reports the percentage of each ciliary structure observed in RPE1 cells and human fibroblasts. Where appropriate, we also added counts and percentages in the Results to improve clarity.

2. As noted above, the findings presented here are presented, to some extent, as representative of all modes of cilium assembly. While some of the vesicle docking and fusion events may commonly occur, it is not yet clear that these processes are always required for ciliogenesis across cell types and species.

Response Reviewer #1 Main Point #2:

We appreciate the reviewer's thoughtful comments on the diversity of ciliogenesis pathways. In response, we revised the manuscript to more clearly distinguish between intracellular and extracellular ciliogenesis. We also strengthened the wording in the Introduction and Results to ensure that our conclusions are explicitly framed within the intracellular pathway as studied here (RPE1 cells and human fibroblasts).

Examples of these edits include:

Lines 199-206: "Interestingly, a fully PM docked MC was observed in a 3h serum-starved RPE1 reporter cell suggestive of the extracellular ciliogenesis pathway, whereby DAs can directly dock to the PM prior to axoneme growth. However, two similarly serum-starved RPE1 reporter cells showed MCs partially docked to the PM through EMC-like structures, with some DAs more than 30 nm away from the PM (Fig. 2d, e, and Supplementary Movie 6). These partially PM docked MCs with EMC-like structures support requirements for vesicular docking to DAs consistent with the intracellular ciliogenesis pathway."

Lines 449-458: "Recognizing the limitations of static EM in establishing strict temporal order, we leveraged the asynchronous nature of ciliogenesis resulting from serum-starvation and performed FIB-SEM on two different cultured cell types. To further enrich for early stages of this process, we used knock-out/knockdown of ciliogenesis regulators in RPE1 cells. Moreover, we incorporated CLEM with FIB-SEM and examined fixed and live-cell SRM imaged cells to strengthen dynamics correlations for membrane-MC ciliogenic processes. Together these studies revealed new insights into the intricate steps of MC-associated vesicle docking, fusion and reshaping, along with establishing new mechanistic connections to MC uncapping and TZ protein recruitment function in intracellular ciliogenesis in RPE1 and human fibroblasts (Fig. 8e)."

Lines 535-538: "Overall, our work indicates roles for proteins on the membrane and at the distal end of the MC in establishing the membrane curvature of CCV and TCV structures in cells using the intracellular ciliogenesis pathway."

The reviewer's comments regarding whether these steps are obligate are addressed further in Response to Reviewer #1, Main Point #3:

3. Some aspects of the sequence of events are not definitively established given the limitations of EM analysis in fixed cells (and at a single timepoint). For example, the proposed progression from DAV to CCV to TCV to CV to cilium relies on few instances of CVs being observed. Can it be concluded with certainty that the CV is an obligatory intermediate step? Alternatively, if the authors propose that it is an obligatory step, is its rarity explained by the timepoint chosen for analysis (seemingly a suitable one since ~30% of cells have formed cilia while others are at early stages) or the possibility that the CV stage is a particularly short-lived intermediate? Likewise, in how many instances were TCV states observed (it would be helpful if these were included in the summary presentation in Supplementary Table 1)? Given the rarity of TCV and CV observations, additional supporting evidence/explanations or tempered conclusions might be warranted.

Response Reviewer #1 Main Point #3:

We appreciate the reviewer's valuable comments and suggestions. We acknowledge that relying on single time-point EM snapshots poses limitations regarding inferences about strict temporal sequencing. As highlighted by the reviewer, the widespread presence of CCV and some cilia at 3 hours of starvation suggests that TCV and CV may represent transient structures. In reference to Reviewer #1, Point #2, we have incorporated a statement addressing this point in the discussion section on lines 164-172 as follows:

"The more prominent detection of CCV compared to CV in serum-starved RPE1 and human fibroblasts expressing GFP-CETN1 (Supplementary Table 1) suggests the CCV is longer-lived intermediate than the CV, which is surprising given CVs are a hallmark structure of the intracellular ciliogenesis pathway from previous TEM studies 7, 13. However, 3D structural analysis suggests that the CCV can be misidentified as CV and DAV stages of ciliogenesis depending on the specific longitudinal EM section of the MC being examined (Fig. 1g, h and Supplementary Fig.1b), which is typical of TEM studies. Likewise, the ciliary sheath/cilia could be misidentified as a CV depending on the sectioning plane, as can be seen in FIB-SEM sections shown in Fig.1e (i, image for DA3)."

In the revised manuscript and Supplementary Table 1, we now specify which cells were imaged at the 3 h and 6 h serum-starvation time points, also addressing Reviewer #2's related question. We agree that the relatively infrequent observation of TCV and CV raises the possibility that these are transient and/or non-obligate intermediates. Nevertheless, FIB-SEM analyses in knockout/knockdown backgrounds, together with complementary SRM imaging, support the proposed ciliogenesis progression model. As suggested, we expanded the Discussion to acknowledge these limitations and to provide alternative explanations for the rarity of TCV/CV (lines 553–561), as well as to discuss how CCV/TCV architectures may contribute to later cilium-base organization (lines 563–570). We also tempered statements in the Abstract, Results, and Discussion accordingly.

Lines 553-561: "While the majority of the axoneme-less RPE1 cells, regardless of serum treatment, and all the human fibroblasts displayed ciliogenic membrane structures consistent with the intracellular ciliogenesis pathway, the observed partially or full docked MC to the PM could

suggest that RPE1 cells may also employ the extracellular ciliogenesis pathway. An alternative explanation for these ciliogenesis structures is that DA docking with the PM requires at a minimum DAVs on the DAs to mediate fusion with the PM, rather than direct DA-PM docking. This could involve the tubulation of DAVs and the formation of an EMC similar to those observed on CCVs and TCVs."

Lines 563-570: "The frequent observation of EMC structures and partially PM-docked centrioles also points to a mechanism in which tubular membrane organization formed on the MC in the cytoplasm is maintained once the CCV/TCV docks to the PM. This is consistent with the toroidal membrane structure at the base of the ciliary sheath and cilium. This proposed mechanism, along with the infrequent observation of CVs in wild-type cells, raises questions about requirements for CV in ciliogenesis. One possibility is the CVs are only observed in cells where the uncapped MC was unable to establish or fully complete docking to the PM, as may be the case in RAB8 depleted cells."

4. It is surprising that so many RPE1 cells in serum-fed conditions have DAVs. Can these DAV stages be observed by any fluorescence microscopy markers? Did the authors make similar observations in other cell types and/or at low cell densities? It is likewise surprising that CEP164 is not needed for DAV docking. Is this true for other DAPs (e.g. DAPs such as CEP83 that lie at a more fundamental position in hierarchy of distal appendage assembly)? Loss of these DAV structures in a full distal appendage knockout background would help confirm that the structures identified as DAVs in FIB-SEM are in fact bona fide DAVs. Lastly, given the prevalence of DAVs under non-ciliating conditions, is the statement " Together these results confirm that DAV docking at the MC is associated with ciliogenesis progression" (lines 169-170) accurate?

<u>Response Reviewer #1 Main Point #4:</u>

We thank the reviewer for this insightful comment and suggestions. We were also surprised to observe DAVs in non-ciliating cells. To eliminate high cell density as a confounding factor, all FIB-SEM analyses were conducted on RPE1 cells maintained at low density (less than 50%), thereby preventing the initiation of ciliogenesis in serum-fed cells, which is consistent with published literature. We apologize that this detail was not included previously; we have now added it to the Methods (lines 718–721) as follows:

Lines 718-721: "cells were grown on alphanumerically coded gridded coverslips (MatTek) to ~30-50% confluence with and without serum as indicated. CLEM studies with RPE1 GFP-CETN1 or GFP-CETN1+SMO-tRFP cells were performed under serum-fed and/or serum-starvation (3 or 6h) treatments."

Unfortunately, we were unable to perform additional FIB-SEM studies in other serum-fed cell types during the revision period due to the limitations described in Response to Reviewer #2, Major Point #1.

As shown in Fig. 2a, FIB-SEM reveals numerous vesicles >30 nm from the DAs of the mother centriole. SIM^2 has ~80 nm lateral (xy) resolution and ~300 nm axial (z) resolution, which limits our ability to determine whether SMO-GFP-positive vesicles are physically docked to DAs. Accordingly, although SIM^2 shows SMO-GFP vesicles in the vicinity of the DA region under serum-fed conditions, we cannot confidently assign these as DA-docked. In live-cell SIM^2 imaging,

DAV docking can be inferred more reliably when vesicle association is followed by cilium formation (e.g., Fig. 4d).

The suggestion of knocking-out CEP83 is an interesting idea, and we have generated a CEP83-KO cell line. However, without the DAs it would not be possible to compare the DAV docking distances (no DAs present) to the DAs by FIB-SEM. We had also hoped it might be possible to use SRM SIM^2 to examine this docking behavior with DA protein KO and wild-type, however at this resolution it is not possible to discriminate MC docking versus vesicles nearby without subsequent cilia assembly as stated above. Consistent with this limitation, SMO-GFP-positive vesicles were detected near the MC in wild-type, CEP164-KO, and CEP83-KO cells using SRM SIM^2. Although CEP164-KO cells appeared to have more vesicles near the DA region than CEP83-KO cells, we cannot confidently conclude that these vesicles are DA-docked. We therefore did not include these preliminary SIM^2 observations in the revised manuscript. We have added the following statement to describe the DAVs observed:

Lines 546-547: "It remains unclear whether these vesicles are stably docked or dynamically associated with the MC DAs."

We also toned down the statement on the previous lines 169–170 and replaced it with the following:

Lines 229-231: "These results suggest that ciliogenesis progression is associated with bigger DAVs, which could result either from the docking of larger vesicles to the MC or from DAV fusion with smaller vesicles transported to the MC."

Other points

1. Can TZ recruitment relative to other steps be clarified/illustrated in the model shown in Fig. 8e?

Response Reviewer #1 Other Point #1:

We thank the reviewer for this suggestion. We have clarified TZ accumulation in the model presented in Fig. 8e.

2. Fig 4d: Why does the apparent SmoM2 diameter decrease significantly after the CCV stage? Is this expected for e.g. a CV stage?

Response Reviewer #1 Other Point #2:

We thank the reviewer for asking for clarification on this point. This could be due to the increased accumulation of SMOM2-GFP in a short cilium, which yields a smaller apparent diameter relative to earlier intermediates. We have added the following statement to the results section on lines 284-287: "SMOM2-GFP localization shifted from overlapping with CEP83 to centering within the CEP83 ring; this shift may coincide with axoneme growth, given SMOM2-GFP stronger accumulation in the ciliary membrane than in the preceding vesicular ciliogenesis structures."

We also added the following to the discussion on lines 502-504: "Live SRM analysis of ciliogenesis in RPE1 cells showed that SMOM2-GFP exhibited a stronger consolidated

localization at the center of the MC, consistent with the establishment of the ciliary membrane, axoneme and a functional TZ."

3. The analysis of EHD1 over-expression and its impact on CP110 removal in serum-fed cells is interesting. How does GFP-EHD1 over-expression impact initial stages of ciliogenesis in serum-fed cells (apart from CP110 removal) without leading to a significant increase in cilia formation? Can SRM imaging reveal if these cells form CCV or toroidal or CV structures (or are arrested at a specific stage in this progression)?

Response Reviewer #1 Other Point #3:

We apologize for any confusion regarding the effects of EHD1 overexpression on ciliation. As shown in the revised manuscript (now Fig. 6h, i), GFP–EHD1 expression significantly increases both CP110 removal and ciliation. However, the fraction of cells lacking CP110 exceeds the fraction of ciliated cells, indicating that CP110 loss does not invariably correspond to a detectable cilium under serum-fed conditions. This may reflect the asynchronous nature of ciliogenesis and/or ciliary resorption in cycling, serum-fed cultures. Variability in transient EHD1 expression levels may also contribute. We have added the following text to the Results (lines 382–386) to clarify this interpretation:

Lines 382-386: "Notably, not all RPE1 cells observed to have lost CP110 from the MC present with cilia. This could be attributed to the asynchronous nature of ciliogenesis or to cilia resorption occurring in cells cultured with serum. Alternatively, ciliogenesis progression could be limited by the levels of transiently expressed EHD1 proteins."

The reviewer also made a good suggestion to examine cells which did not form cilia for CCV and TCV structures. Because GFP–EHD1 is transiently associated with early ciliogenesis membranes and the ciliary pocket (Lu, Insinna et al. 2015), we included a stable membrane marker in this assay. Specifically, we assessed SMO-GFP-positive CCV/TCV-like structures in non-ciliated RPE1 cells after transient expression of HA–EHD1. Using SIM^2 we found a few examples where HA-EHD1 and SMO-GFP partial colocalization in CCV/TCV-like structures. We have included microscopy images for these findings in Supplemental Figure 9 and have added the following statement to the results section starting on line 386:

Lines 386-389: "Indeed ciliogenesis progression prior to the ciliary axoneme growth stages is suggested by the observed partial colocalization of SMO-GFP with transiently expressed HA-EHD1 on CCV-like structures in RPE1 cells that lacked a ciliary SMO-GFP signal in the central region of the MC (Supplementary Figure 9)."

4. The images in Supplementary Table 1 for EHD1 RNAi in a wildtype RPE1 background look different than for EHD1 RNAi in the background with fluorescent markers expressed. Specifically, the former show relatively numerous/large DAVs and some CCVs, whereas the latter showing smaller and fewer DAVs in a manner more reminiscent of the CEP164 KO cells. Can the authors clarify these apparent differences and which EHD1 RNAi FIB-SEM data are described in the Results?

Response Reviewer #1 Other Point #4:

We apologize for any confusion caused by our description of these datasets. For the CP110 analysis, we performed FIB-SEM specifically on siEHD1-treated cells in which GFP–CP110 was fully retained at the mother centriole, consistent with effective EHD1 depletion; in these cells, the absence of CCVs correlates with CP110 retention. By contrast, the siEHD1-treated RPE1 GFP-CETN1 cells shown in Fig. 3f were randomly selected and predominantly exhibited DAVs, with CCVs observed in only two cells. In the revised manuscript, we compared DAV number and surface area in siEHD1-treated RPE1 cells with versus without SMO-tRFP and did not detect a statistically significant difference, which we describe in the legend of Fig. 5b. We have also modified the results section as follows:

Lines 319-322: "Consistent with this proposed MC uncapping model, CLEM FIB-SEM imaging of RPE1 GFP-CP110 SMO-tRFP SNAP-CETN1 cells depleted of EHD1 to presuggesting7and CCV establishment showed that cells retaining GFP-CP110 at the MC had DAVs similar in size to RPE1 GFP-CETN1 cells depleted of EHD1 (Fig. 5b, Supplementary Table 1)."

One important oversight in our previous submission was the segmentation shown in the Supplementary Table 1 for a cell shown in Fig. 5b was incorrect. Two vesicles were missing in the Supplementary Table 1 images shown previously. We have corrected this mistake in the revised Supplementary Table 1.

5. Lines 363-365. "These observations support requirements for tubular membrane assembly in instructing MC uncapping and indicate that RAB8-dependent CCV "C" gap closure upstream of TCV assembly is important for completing CP110 removal" Is it not equally or more likely that CP110 removal enables RAB8-dependent "C" gap closure and TCV formation rather than vice versa?

Response Reviewer #1 Other Point #5:

We thank the reviewer for pointing this out. We have modified the following sentence to the results section as suggested.

Lines 420-422: "These observations support requirements for tubular membrane assembly in instructing MC uncapping and indicate that RAB8-dependent CCV "C" gap closure upstream of TCV assembly is associated with CP110 removal."

6. It seems possible that TZ protein recruitment versus TZ assembly (e.g. into Y-linked architecture) are distinct steps, with the former occurring at the CCV stage, while the second may occur later and be subject to additional regulation and prerequisite steps in cilium formation. It might be helpful to discuss this point in the context of the authors' observations.

Response Reviewer #1 Other Point #6:

We thank the reviewer for pointing out this important distinction. We have discussed this point further in modifying our statement in the discussion.

Lines 499-502: "Our observations that TZ proteins accumulate at sites of CP110 loss associated with CCV/TCV structures suggests that the TZ is organized at these stages, however, it is not clear if functional Y-links architecture of the TZ is established."

7. It is surprising that IFT88 KO leads to accumulation of cells with the MC docked to the PM, a stage that is not typically seen in the sequence of events associated with ciliogenesis in wildtype cells. Can the authors clarify how they feel this observation fits with their model and the step(s) at which IFT88 may act?

Response Reviewer #1 Other Point #7:

We thank the reviewer for the insightful comments. Although we do not typically see PM-docked MC, we think it is important to note that we did see this structure in RPE1 GFP-CETN1+SMO-tRFP cells (Supplementary Table 1 – "PM-docked" structure).

We clarified how observed structures align with our model by adding PM docking details to the model in Fig. 8e, as suggested by Reviewer #3. An important premise is that vesicular membranes on DAs are likely needed to dock to the PM in cells using the intracellular pathway. This may occur as early as the DAV stage. Partially PM docked MCs (Fig. 2e) may indicate prior fusion of DAVs or CCVs with the PM, followed by DAV docking to DAs not yet associated with the PM. In IFT88 KO cells, we observed DAVs and TCV by FIB-SEM, which is consistent with the vesicular membrane docking mechanism on the DA. Previous work also showed that SMO-GFP vesicles could be recruited to the basal body when IFT88 was knockdown by RNAi in RPE1 cells(Joo, Kim et al. 2013), and we have added this description on lines 425-427: "Notably, RNAi knockdown of IFT88 in RPE1 cells did not prevent SMO-GFP from localizing to the MC during ciliogenesis even though most cells failed to develop cilia."

As mentioned, we have expanded our discussion on this mechanism on lines 553-573 and how we think the IFT88 KO data fits this model.

Lines 553-573: "While the majority of the axoneme-less RPE1 cells, regardless of serum treatment, and all the human fibroblasts displayed ciliogenic membrane structures consistent with the intracellular ciliogenesis pathway, the observed partially or full docked MC to the PM could suggest that RPE1 cells may also employ the extracellular ciliogenesis pathway. An alternative explanation for these ciliogenesis structures is that DA docking with the PM requires at a minimum DAVs on the DAs to mediate fusion with the PM, rather than direct DA-PM docking. This could involve the tubulation of DAVs and the formation of an EMC similar to those observed on CCVs and TCVs. Notably, we did not observe EMCs on DAVs in EHD1 depleted cells, supportive of a role for EHD in the developing primary cilium gaining access to the extracellular space. The frequent observation of EMC structures and partially PM-docked centrioles also points to a mechanism in which tubular membrane organization formed on the MC in the cytoplasm is maintained once the CCV/TCV docks to the PM. This is consistent with the toroidal membrane structure at the base of the ciliary sheath and cilium. This proposed mechanism, along with the infrequent observation of CVs in wild-type cells, raises questions about requirements for CV in ciliogenesis. One possibility is the CVs are only observed in cells where the uncapped MC was unable to establish or fully complete docking to the PM, as may be the case in RAB8 depleted cells. This DA-membrane docking mechanism to the PM could also explain the range of ciliogenesis structures from DAV to TCV to MC-PM docked observed in IFT88 KO cells, although it remains to be determined how IFT88 affects pre-axonemal membrane organization at the MC."

8. What exactly is shown in Supp. Fig 6? A series of different cells imaged by SRM (or different z planes or time points from one cell)?

Response Reviewer #1 Other Point #8:

We apologize for the lack of clarity in the legend. Each panel shows a different cell. The legend for Supplementary Fig. 6 now reads:

"Images show cells at different stages of ciliogenesis initiation marked by CP110 removal with associated SMO-GFP positive membrane docking. Cells were imaged with OMX SR imaging system."

9. Lines 389-390: "dynamics in the low range nanoscale range". Typo

Response Reviewer #1 Other Point #9:

We thank the reviewer for finding this mistake and we have corrected the typo on lines 448-449.

10. Lines 435-437: "What is clear is that TZ protein accumulation at the MC coincides with CCV and TCV assemble suggestive that the TZ is being organized." This wording is confusing.

Response Reviewer #1 Other Point #10:

We agree with the reviewer and have modified this sentence as follows on lines 449-502: "Our observations that TZ proteins accumulate at sites of CP110 loss associated with CCV/TCV structures suggests that the TZ is organized at these stages, however, it is not clear if functional Y-links architecture of the TZ is established."

Reviewer #2

In the article "Characterization of membrane structures regulating primary ciliogenesis by quantitative isotropic ultrastructure imaging", Lu and colleagues investigate the early steps of ciliary vesicle assembly during ciliogenesis. They show, for the first time, that the enlargement and fusion of small vesicles at the mother centriole (MC) into novel tubular C-shaped and toroidal membrane structures is a critical step in primary cilium formation. The authors demonstrate the importance of these membrane intermediates in recruiting and stabilizing transition zone complexes, as well as in the removal of the capping complex CP110/CEP97—both of which are essential for ciliogenesis.

Furthermore, they describe the involvement of molecular regulators such as EHD1 and RAB8 in orchestrating the membrane trafficking and fusion events that lead to the formation of these intermediates. This study highlights the power of 3D isotropic ultrastructure imaging in uncovering dynamic membrane remodeling processes during organelle biogenesis. The combination of vEM-FIB-SEM with correlative light and electron microscopy (CLEM), and photonic super-resolution microscopy—including live and fixed SIM, STED, and expansion microscopy—constitutes an impressive tour de force.

While I acknowledge the tremendous amount of work, the high quality of the data, and the novel biological insights presented, I would like to raise several crucial points that need to be addressed before this article can be considered for publication.

We appreciate the thoughtful suggestions and comments made by the reviewer about this study, and we have addressed all their comments below.

Major points:

1) Throughout the paper, the authors imply a notion of temporality in the maturation of ciliary vesicles. This is an important point of the paper that is not well supported by the data. Indeed, only one time point of ciliogenesis is used. Although the authors performed experiments at 3h and 6h post-serum starvation, they pooled the results from both time points, making the temporality impossible to establish. They based their conclusion on rare events (sometimes only one cell) observed in non-starved conditions to conclude on the temporality (Figure 1 and 2). The authors should consider: i) increasing the number of events in the "non-starved condition"; ii) adding intermediate time points during ciliogenesis (e.g., 3h versus 6h); or iii) removing / down tuning their interpretation regarding temporality

Response Reviewer #2 Major Point #1:

We thank the reviewer for the comments and agree that inferring temporality from single time-point EM is inherently limited (see also Response to Reviewer #1, Main Point #3). Additionally, we have addressed the reviewer's points (i–iii) below and have expanded the Results and Discussion to more clearly describe our approach, including the following statement (Discussion, lines 449–454):

Lines 449-454: "Recognizing the limitations of static EM in establishing strict temporal order, we leveraged the asynchronous nature of ciliogenesis resulting from serum-starvation and performed FIB-SEM on two different cultured cell types. To further enrich for early stages of this process, we used knock-out/knockdown of ciliogenesis regulators in RPE1 cells. Moreover, we incorporated CLEM with FIB-SEM and examined fixed and live-cell SRM imaged cells to strengthen dynamics correlations for membrane-MC ciliogenic processes."

i) Per the reviewer's suggestion, we performed additional FIB-SEM imaging on five serum-fed ("non-starved") RPE1 cells. These new datasets support our model that vesicles consistent with DAVs are present at the distal appendage region of the mother centriole in most serum-fed cells. We could only obtain a limited number of new FIB-SEM data sets from the NCI core facility during the revision period due to several factors that were out of our control: 1) materials needed for FIB-SEM were affected by tariffs imposed prior and after receiving the reviewed manuscript in May 2025, 2) NCI then faced government budgetary cutbacks of contractors which resulted in a contractor staff who performed the FIB-SEM in the NCI core facility being laid off in August 2025, 3) the remaining core staff member who could operate the FIB-SEM subsequently left the facility in September 2025, and a replacement has not been found at this time. Another challenge faced throughout the course of this study is the sole FIB-SEM in the NCI core is typically down

for repair a few months of the year. Finally, we estimate that >50% of the CLEM samples prepared provided to the facility are unusable due to machine malfunction and image alignment issues.

ii) We apologize for not explicitly describing the FIB-SEM structures observed in the 3 h and 6 h serum-starved samples. We agree that our prior description of these datasets was too brief. As suggested, we now provide a more detailed description of the cells and conditions analyzed in Supplementary Table 1. These data show that early ciliogenesis structures, including DAVs and CCVs, are present in both the 3 h and 6 h serum-starved samples. These clarifications do not alter our proposed model; the relevant text now appears beginning at line 126: "To enrich for cells at pre-axonemal ciliogenesis stages, we expressed a tagRFP (tRFP)-fused SMO, a ciliary transmembrane receptor involved in the Hedgehog pathway, to facilitate monitoring of cilia formation. Serum starvation promotes asynchronous ciliation in RPE1 cells, with early ciliogenesis structures such as DAVs and CVs expected after 3h or 6h serum removal. FIB-SEM imaging was performed on cells subjected to 3h and 6h of serum-starvation, generating high resolution aligned image stacks for five and six cells, respectively, under these treatment conditions."

iii) Given the limited ability to acquire additional FIB-SEM datasets (as noted in i), and as recommended by Reviewers #1 and #2, we have tempered language throughout the manuscript regarding strict temporal ordering of events.

2) Quantifications are missing in several key experiments to support the conclusions:

Response Reviewer #2 Major Point #2:

We thank the reviewer for these suggestions.

• Additional graphs showing the quantification regarding the intermediate stage of vesicle formation would be welcome, notably for the tubulogenic extension from the CCV phase and the EMC events, which seem to be quantifiable according to the main text, but no apparent quantification appears in Figure 1.

We apologize for any confusion regarding our presentation of the CCV-tubule and CCV-EMC stages. This quantification was previously shown in Supplementary Fig. 1b; we have now consolidated all intermediate-stage quantification into Supplementary Table 1.

• Quantification of the behavior of GFP-SMO over time (from dots to C-shape to donut) in relation to the appearance of the cilium would be appreciated to give a temporal dimension to ciliary vesicle maturation.

We agree with the reviewer that quantifying SMO-GFP behavior would have been informative. However, several technical limitations prevent accurate measurement using SIM in this study. SIM and SIM^2 reconstruction steps (phase demodulation, OTF weighting, Wiener filtering/deconvolution, optical-sectioning, background subtraction) can alter signal intensities. Additionally, live-SIM imaging is constrained by photobleaching and phototoxicity at the frame rates, as well as by cell-cell variability in protein expression-levels, which preclude rigorous accurate bulk signal intensity comparison. Due to these factors, we believe it is not possible to accurately quantify SMO-GFP intensity from dots to C-shape to donut in this study. Accordingly, we have toned down our conclusion from this study as follows:

Lines 284-289: “SMOM2-GFP localization shifted from overlapping with CEP83 to centering within the CEP83 ring; this shift may coincide with axoneme growth, given SMOM2-GFP stronger accumulation in the ciliary membrane than in the preceding vesicular ciliogenesis structures7. Remarkably, prior to this ciliogenic state the DA associated SMOM2-GFP shifts from an asymmetric DAV or CCV-like appearance to a ring-like structure within a 10-minute interval (Fig. 4d, Supplementary Fig. 4 and Supplementary Movie 8).”

• Quantification of the correlation between CP110 removal and SMO-vesicle accumulation, as well as TZ accumulation, is necessary to support the conclusion regarding CP110 regulation.

We thank the reviewer for this suggestion. Because of challenges we described above in quantifying our SRM SIM data with SMO-GFP and CP110, we performed new spinning-disk confocal experiments (Supplementary Fig. 8a) to quantify the relationship between CP110 loss and SMO accumulation. We performed a parallel analysis for the TZ protein GFP–B9D2 (Supplementary Fig. 8e). In both assays, cells were categorized as CP110-capped, partially uncapped, or fully uncapped based on CP110 signal at the MC. SMO-GFP and GFP–B9D2 intensities increased significantly with progressive CP110 removal. We incorporated these quantitative results into the revised Results section.

Lines 305-306: “We show quantitatively that SMO-GFP accumulation on the MC is correlated with the progressive removal of CP110 from the MC (Supplementary Fig. 8a).”

Lines 332-334: “Similar to results with SMO-GFP (Supplementary Fig. 8a), a correlation was observed with the accumulation of the TZ protein GFP-B9D2 at the MC and progressive CP110 removal by confocal imaging (Supplementary Fig. 8e).”

We also assessed the relationship between CP110 levels and the TZ proteins TMEM67 and RPGRIP1L at the MC using the ExM datasets in Fig. 5d,e. Across experiments, CP110 intensity was negatively correlated with both TZ markers. Pairwise comparisons of regression slopes revealed no significant differences among experiments indicating statistically similar slopes within the limits of experimental power. These analyses further support an association between CP110 loss and TZ protein recruitment to the MC. We report this analysis in the Fig. 5d,e legends and in the accompanying Source Data spreadsheet.

Finally, to clarify the spatial distributions of CP110 and TZ proteins in our ExM datasets, we added fluorescence intensity profiles to Fig. 5d,e and included an additional panel in Supplementary Fig. 8d, which quantitatively illustrates differences in MC localization.

• All quantifications represented as ‘pie charts’ need to be revised and presented as graphs that allow for visualization of i) experimental reproducibility; ii) percentage; and iii) number of total events quantified.

We thank the reviewer for this suggestion. All pie charts have been replaced with plots and corresponding totals, and total events quantified are indicated in legends/tables.

3) The use of ‘pro-ExM’ to study centrioles is questionable here. As demonstrated in Gambarotto et al., 2019 (https://doi.org/10.1038/s41592-018-0238-1), the ‘pro-ExM’ protocol is not suitable for isotropic expansion of centrioles. Pre-expansion staining of such a compact structure leads to limited epitope accessibility, and sample fixation triggers non-isotropic expansion of this large

macromolecular complex. For these reasons, interpretation of asymmetric CP110 staining is questionable, as it could be due to poor expansion. I therefore strongly recommend using Ultrastructure Expansion Microscopy as described in this paper (https://doi.org/10.15252/embj.2022112107), which uses U-ExM to study centriolar architecture in RPE1 cells, including similar stainings (CEP164 and CP110). Moreover, expansion validation (expansion factor) is missing.

Response Reviewer #2 Major Point #3:

We apologize for any confusion our description of this experimental procedure may have caused. We acknowledge that our approach was not adequately described in the Methods section. We were aware of the limitations of the 'pro-ExM' protocol for under-expansion of centrioles that was described by the reviewer. We had modified the 'pro-ExM' protocol to ensure isotropic expansion of centrioles and we realized, thanks to the reviewer, that we failed to adequately describe these modifications in the original submission. The buffer composition and digestion time listed in the Methods section reflect our modified protocol, which was partially adapted from other published papers(Halpern, Alas et al. 2017, Zhao, Bucur et al. 2017). Importantly, we used acetylated-tubulin and CEP164 antibodies as internal checks for isotropy for this modified protocol. In our revised manuscript, we have clarified this in the updated Methods section starting at line 657.

Lines 657-659: "The ExM protocol generally followed proExM methods with modifications to improve uniformity of centrosome expansion. The acrylamide concentration was increased as described in (ref 80), and EDTA concentration was increased as described in ref(81)."

To further ensure that our modified proExM protocol yields results comparable to U-ExM, we performed U-ExM staining for CEP164, CP110, and RPGRIP1L as suggested by the reviewer. These experiments confirmed the findings obtained with the modified proExM approach (new Supplementary Fig. 8d). The TMEM67 antibody, which targets a transmembrane TZ protein, did not perform reliably under our conditions using U-ExM. We now report the measured expansion factor and describe the validation in the Methods (lines 668 and 682).

4) A thorough description of the culture conditions—particularly medium composition and seeding density for the ciliogenesis experiments—is missing. Given the variety of protocols used to study ciliogenesis and the variability in results in the literature, providing this information is essential.

Response Reviewer #2 Minor Point A #4:

We thank the reviewer for this suggestion, and a similar point was raised by Reviewer #2 about cell density (see response to Response Reviewer #1 Main Point #4). We have added a comprehensive description of medium composition, serum conditions, and seeding density used for ciliogenesis experiments to the methods sections "**Cell lines**", "**Immunofluorescence**" and "**CLEM sample preparation**".

Minor points:

A. General comments:

1. I recommend a careful read of the manuscript to check for spelling and typos, particularly in the figure legends.

Response Reviewer #2 Minor Point A #1:

We thank the reviewer for this comment. We carefully proofread the manuscript and corrected spelling and typos.

2. The definitions of PCV, CV, CCV, EMC, etc., should be listed in a table or in an inset present in all figures where these abbreviations are used. In Figure 1, for instance, the definition of CCV appears too late in the description (only when mentioning Figure 1g in the main text), while the abbreviation is already used in the quantification of Figure 1d, making reading difficult.

Response Reviewer #2 Minor Point A #2:

We thank the reviewer for this suggestion. We have included the abbreviations in Supplementary Table 2, as suggested.

3. The use of the dash symbol (–) is not consistent across the manuscript. Sometimes it indicates the presence of serum (e.g., Figure 1b legend: "Quantification of ciliary structures identified by FIB-SEM in serum-fed RPE1 cells") and elsewhere the absence (e.g., Figure 1h and 1i: "CCV, human fibroblast – serum"). For consistency, the symbol should not be used to indicate serum absence, which should be referred to as "starved condition."

Response Reviewer #2 Minor Point A #3:

We thank the reviewer for pointing this out. Throughout the manuscript, we replaced "– serum" with "serum-starved" and "+ serum" with "serum-fed" for consistency.

4. The current names of the supplementary videos are unclear and do not allow easy identification of their correspondence to the text. Additionally, a legend for each video would be welcome (especially for Movie S8).

Response Reviewer #2 Minor Point A #4:

We thank the reviewer for this suggestion. We have renamed videos and added legends to each video in the supplementary file as suggested.

B. Results interpretation:

1. In Figure 3a, CEP164 KO cells show DAV docking, likely due to the presence of intact DAs. The proportion of cells still containing DAs should be quantified to support the presence of DAV.

Response Reviewer #2 Minor Point B #1:

We appreciate the reviewer's suggestion. All CEP164 KO FIB-SEM samples showed nine DAs, and although Supplementary Table 1 displayed the DAs for CEP164-KO cells, we had not explicitly stated that there were nine presents. To make this clear, we have now included the following statement in the results section on lines 215-216: "Nine DAs were observed in the CEP164 KO cells based on FIB-SEM analysis (Fig. 3a, Supplementary Table 1)."

2. Figure 4a – The conditions are unclear. Are these images from cells in the presence or absence of serum? A sign (+/–) is missing in the right panel. It is also surprising to use a non-starved (+ serum) image to highlight CCV presence, which (according to Figure 1) is observed in only one cell in serum. Could the authors clarify whether the 'CCV + DAV' situation represents an intermediate stage in serum-fed cells? If not, they should use starved cells for this observation for consistency.

Response Reviewer #2 Minor Point B #2:

We apologize for any confusion caused by the figure labeling; we have corrected the panel labels and legend accordingly. Although we observed only one CCV-containing MC in the serum-fed FIB-SEM datasets, we believe this observation is relevant because serum-fed RPE1 cultures can exhibit a low baseline level of ciliation (~5%). Consistent with this, among 13 serum-fed RPE1 cells imaged by FIB-SEM, we observed one cell with a short cilium/ciliary sheath. Together, these observations indicate that CCV-associated MCs can occur under both serum-fed and serum-starved conditions. Importantly, CCV-containing MCs can retain DAVs, which supports our proposed assembly of TCVs. Consequently, we have included CCV from serum-starved and serum-fed conditions to describe these events. We clarified this point in the Results (lines 265–268), as follows: Notably, FIB-SEM analysis revealed that ~40% of all cells observed with CCVs had associated DAVs in the gap between the ends of the CCV (Fig. 4a, Supplementary Fig. 2b, Supplementary Movie 7 and Supplementary Table 1), that we refer to as the "C" gap (Fig. 4b)."

As suggested, we removed the serum-fed MC example with a CCV in Fig. 4a.

3. The use of SMO-GFP/SMO-tRFP (Figures 3g, 4e, Supp6) versus GFP-SMOM2 (Figure 4d) is puzzling. How could the authors visualize the pre-ciliary membrane with regular SMO in Figures 3a/4e but require a specific SMO variant in Figure 4d? This difference should be clarified.

Response Reviewer #2 Minor Point B #3:

We thank the reviewer for this question. Both SMO-GFP and SMOM2-GFP label early ciliary vesicles in fixed RPE1 cells, as reported previously (Lu, Insinna et al. 2015, Insinna, Lu et al. 2019). For live-SIM experiments, we selected SMOM2-GFP versus SMO-GFP based on expression levels and signal-to-background when co-expressed with other markers. We have clarified this in the Results (lines 276–278), as follows: "we established an RPE1 cell line expressing the M2 variant of SMO (SMOM2-GFP), which shows less cytoplasmic signal around the MC than SMO-GFP, and the DA protein CEP83 fused to a SNAP-tag."

4. Figure 4d – It's unclear whether the same centriole/cilium is followed over time. The change in orientation from transverse to longitudinal view at t:70, 80, and 90 is confusing and may suggest

two different samples. If the same sample is followed from t:0 to t:90, the change in orientation should be clearly noted.

Response Reviewer #2 Minor Point B #4:

We apologize for any confusion caused by this figure. We confirm that the same centriole is followed throughout the time series. We added an explicit note that DA-ring orientation can change as the MC rotates relative to the coverslip during ciliogenesis on lines 1268-1269: "Note that the MC has rotated to a side view in the imaging plane at the 70, 80, and 90 min time points."

5. Figures 5d, e – The interpretation of CP110 staining evolution exceeds the presented results. Adding a temporal element or correlating with ciliary growth would help evaluate CP110 removal more accurately.

Response Reviewer #2 Minor Point #5:

We appreciate the reviewer's concern. We address this point in Response to Reviewer #2, Major Point #2, and we have also added new quantitative data supporting progressive CP110 loss from the MC as SMO-GFP or the TZ protein B9D2 accumulates, as requested. We also rephrased the Results to avoid over-interpretation (lines 336–345), as follows: "Strikingly, using higher-resolution ExM SRM imaging approaches with fluorescence profile plotting the MC accumulation of the TZ proteins TMEM67 and RPGRIP1L could be observed at sites lacking CP110 (Fig. 5d, 5e and Supplementary Fig. 8d, f). Notably, MCs with SMO-GFP donut-like structures observed by SIM imaging, suggestive of pre-axonemal structures, displayed TZ proteins CEP290 and TMEM67 accumulation either within or at the edge of the GFP-positive 'donut-hole', respectively (Supplementary Fig. 8g), consistent with the expected position of the TZ on the BB in ciliated cells. Together these findings indicate that MC cap removal correlates with assembly of tubular CCV and TCV structures, and this process is coordinated with recruitment of TZ proteins associated with these membranes during ciliogenesis."

6. Figures 6c, d – The text states: "Immunoblot analysis of coexpression (CO)-IP studies confirmed GFP-CP110 and GFP-CEP97 can interact with HA-tagged EHD1, but not with the other ciliogenesis membrane trafficking regulators tested (Fig. 6c, d)," yet the figures show GFP-CP110 also interacts weakly with PASCIN1, and GFP-CEP97 shows interaction with RAB8 and RABIN8. This discrepancy should be addressed.

Response Reviewer #2 Minor Point B #6:

We thank the reviewer for this suggestion. We revised the text accordingly (lines 358–361), as follows: "Immunoblot analysis of co-immunoprecipitation studies confirmed GFP-CP110 and GFP-CEP97 can interact with HA-tagged EHD1, whereas other ciliogenesis membrane trafficking regulators tested show weak or negligible interaction with the MC cap proteins (Fig. 6c, d)."

7. Figure 6e – The experimental design is unclear. Are cells co-transfected with GFP-CP110 and GFP-CEP97 at various plasmid amounts? The experimental description and figure legend need clarification.

Response Reviewer #2 Minor Point B #7:

We apologize for any confusion about the experimental design our description caused. We have clarified the legend for Fig. 6e on lines 1353-1356 as follows: "293T cells were transfected with plasmids for HA-EHD1 with GFP-CP110 or HA-EHD1 and different amounts of GFP-CEP97. Lysates were immunoprecipitated with anti-HA antibodies. The first and second lanes were transfected with same amounts of GFP-CP110 or GFP-CEP97 plasmids."

C. Figure typos and others:

1. Figure 2a – The green numbers (distances) are hard to read and should be either recolored or numbered and referenced in the legend.

Response Reviewer #2 Minor Point C #1:

We thank the reviewer for this suggestion. We have referenced the numbers in the legend and/or recolored where possible.

2. Figure 2a i) and ii) – Color codes should match across both panels (currently DA is blue in i) and orange in ii)).

Response Reviewer #2 Minor Point C #2:

We thank the reviewer for this suggestion. We have modified the colors to show DA in orange in both i and ii.

3. Supplementary Figure S3a – The "AcTub" channel is magenta, but the legend indicates red. Also, a scale bar is missing. Check S3b as pericentrin in wrong color

Response Reviewer #2 Minor Point C #3:

We thank the reviewer for noticing this issue. We have modified the figure label to match the color code in the figure and added the scale bar.

4. Figure 4c – The largest angle mentioned in the text (149°) does not match the one shown in the figure (153°).

Response Reviewer #2 Minor Point C #4:

We again thank the reviewer for catching this error. We have corrected the angle in the text to match the figure.

5. Figure 4d (and Supp. 4) – Annotations would help visualize vesicular features. The bottom panel of 4d is not explained well. The legend ("MC transverse and longitudinal images...") doesn't clarify its purpose.

Response Reviewer #2 Minor Point C #5:

We thank the reviewer for this suggestion. We have revised the legend and added xyz annotations to clarify what is showing in Fig. 4d.

6. Figure 5a – The left-hand scheme indicating SMO-tRFP position does not match the SIM image. The bottom panel shows a ring-shaped vesicle, while the image depicts a C-shaped one. This should be revised.

Response Reviewer #2 Minor Point C #6:

We apologize for any confusion caused by the fluorescent image. It is not possible to tell the orientation of the MC from GFP-CETN1, and the resolution of this image is lower due to imaging in PBS. We could not use mounting media needed for the highest SIM resolution as these cells were processes for FIB-SEM. Though it appears C-shaped, the signal in the left square likely represents the TCV, while the right square shows the EMC-like portion (see image below). While this looks like C-shape it is more likely that the signal in the left square on the centriole is the TCV and the right square part is the EMC-like portion. We have clarified this point in the figure legend of Fig. 5a as follows,

Lines 1293-1294: "SIM images of the bottom panel showed the side view of the TCV and associated EMC. SIM images of the bottom panel showed the side view of the TCV and associated EMC."

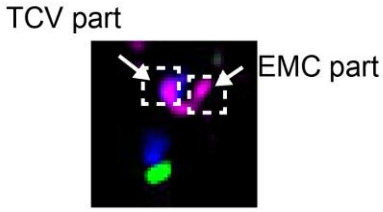

7. Figure S8a – The centriole schematic appears misoriented compared to the microscopy image.

Reviewer #2 Minor Point C #7:

We thank the reviewer for pointing this out. We have removed the centriole schematics as we believe they may have been causing some confusion about what the data shows.

8. Figures 6c, d – The phrase "coexpression (CO)-IP" should be replaced with "co-immunoprecipitation."

Response Reviewer #2 Minor Point C #8:

We thank the reviewer and have changed the text as suggested.

9. In the sentence: “To examine the ciliogenic potential of DAVs, we examined the distribution and organization of these membranes in RPE1 cells grown under non-ciliating and ciliating conditions and in starved cells lacking CEP164,” the conditions are unclear. Is this a repetition, or are “ciliating condition” and “starved cells” different?

Response Reviewer #2 Minor Point C#9:

We apologize for the confusion our statement caused. The text on line # has been revised to provide a separate description of DAVs in RPE1 and CEP164 KO cells.

Lines 223-225: “To examine the ciliogenic potential of DAVs, we examined the distribution and organization of these membranes in the RPE1 reporter cell lines imaged by FIB-SEM grown under non-ciliating (serum-fed) and ciliating conditions (serum-starved).”

Reviewer #3

In this manuscript Lu and colleagues investigate the early stages of primary cilium formation, focusing on the 3D membrane ultrastructure. Utilizing FIB-SEM and super-resolution microscopy, the authors identify two unknown membrane intermediates that are formed prior to the well-established ciliary vesicle (CV): the tubular C-ciliary vesicle (CCV) and the toroidal ciliary vesicle (TCV). Furthermore, the authors analyze the functions of CEP164, EHD1, RAB8 and IFT88 in regulating the formation of these membrane intermediates. Key findings include the requirement for EHD1 and RAB8 in CCV/TCV formation, a novel requirement for CEP164 in vesicle expansion, and confirmation of IFT88's role in the initiation of ciliogenesis. This study proposes a revised architectural framework for early ciliogenesis, emphasizing the tubular membrane intermediates, and highlights the power of isotropic ultrastructure imaging and quantitative 3D analysis. I would support the publication of the manuscript in Nature Communications once the following concerns are addressed.

We appreciate all the comments made by the reviewer about this work.

Specific comments for the authors:

1.RPE1 cells are typically understood to employ the intracellular pathway for ciliogenesis. However, the data presented includes observations of mother centriole docking directly to the plasma membrane, a characteristic of the extracellular pathway. Could the authors discuss these observations in the context of the known RPE1 ciliogenesis mechanism? Does this suggest that RPE1 cells might utilize both pathways, or are these observations indicative of a specific cellular state or context that influences the chosen pathway?

Response Reviewer #3 Point #1:

We thank the reviewer for the insightful question about ciliogenesis pathways. Reviewer #1 raised a similar point. We have addressed this in the response to Reviewer #1 Other Point #7 and included discussion of alternative explanations for the observations in RPE1 cells in the discussion as follows,

Lines 199-206: "Interestingly, a fully PM docked MC was observed in a 3h serum-starved RPE1 reporter cell suggestive of the extracellular ciliogenesis pathway, whereby DAs can directly dock to the PM prior to axoneme growth6. However, two similarly serum-starved RPE1 reporter cells showed MCs partially docked to the PM through EMC-like structures, with some DAs more than 30 nm away from the PM (Fig. 2d, e, and Supplementary Movie 6). These partially PM docked MCs with EMC-like structures support requirements for vesicular docking to DAs consistent with the intracellular ciliogenesis pathway."

2.Figure 2 shows instances where Distal Appendage Vesicles (DAVs) appear to mediate the docking of the mother centriole to the plasma membrane. This observation regarding the role of DAVs in docking to the plasma membrane is not explicitly included or reconciled within the proposed working model presented in Figure 8e, which focuses primarily on the intracellular pathway intermediates (CCV, TCV, CV). Could the authors clarify if the docking of mother centriole to the plasma membrane mediated by early ciliogenesis vesicles is a common observation? If so, they need to integrate this step with their model in figure 8.

Response Reviewer #3 Point #2:

We appreciate the reviewer's points and have addressed the DA-PM docking mechanism further in the new section described in Response Reviewer #3 Point #1in the discussion, as follows:

Lines 553-563: "While the majority of the axoneme-less RPE1 cells, regardless of serum treatment, and all the human fibroblasts displayed ciliogenic membrane structures consistent with the intracellular ciliogenesis pathway, the observed partially or full docked MC to the PM could suggest that RPE1 cells may also employ the extracellular ciliogenesis pathway. An alternative explanation for these ciliogenesis structures is that DA docking with the PM requires at a minimum DAVs on the DAs to mediate fusion with the PM, rather than direct DA-PM docking. This could involve the tubulation of DAVs and the formation of an EMC similar to those observed on CCVs and TCVs. Notably, we did not observe EMCs on DAVs in EHD1 depleted cells, supportive of a role for EHD in the developing primary cilium gaining access to the extracellular space."

We also thank the reviewer for suggesting revisions to the working model in Fig. 8e. We have now incorporated cartoons depicting EMC structures at the DAV-to-CV stage.

3. In Line 164, the authors report that 3/4 of RPE1 cells grown under serum-fed non-ciliating conditions display DAVs or CCVs. However, earlier electron microscopy studies often reported few or no discernible vesicles directly associated with the mother centriole in cycling cells. Could the authors discuss the discrepancy? Whether their cell culture conditions (e.g., cell density) might influence the prevalence or visibility of these early membrane intermediates compared to what has been observed in other studies?

Response Reviewer #3 Point #3:

Reviewer #1 raised a similar point regarding the prevalence of DAVs under serum-fed conditions and potential effects of cell density; we address this in more detail in Response to Reviewer #1, Main Point #4. Briefly, we now specify the cell density used for FIB-SEM (30–50% confluence) in the Methods (line 719). In addition, we acquired FIB-SEM datasets from five additional

independently prepared serum-fed RPE1 GFP-CETN1 cells; four cells exhibited DAV-associated MCs and one cell displayed a short cilium, supporting our model.

We also expanded the Discussion to explain why conventional TEM (single thin sections) could miss these intermediates. In our FIB-SEM datasets, not all distal appendages contain DAVs, so a single longitudinal section may not intersect a DAV-bearing appendage. In addition, with ~70–80 nm section thickness, conventional TEM provides limited axial information, making it difficult to unambiguously assess vesicle docking distances. We added the following statements to address these points:

Lines 460-462: "This enabled the quantitative characterization of different ciliogenesis membrane intermediates through examining their frequencies, sizes, and distributions on the MC, not possible using ~70-80 nm section TEM section imaging."

Lines 545-546: "These DAVs could be missed by single TEM image analysis of MCs (Fig. 1g, h), in part because not all DAs contain these docked vesicles."

4. The authors define CCVs as vesicles that cover more than 50% of the circumference of the DA region. In Line 130, they state that CCVs are present in approximately one-third of the cells. However, this raises the question that what occurs in the remaining two-thirds of the cells? The authors ought to present (quantitative) data on their observations of these cells. Also, there is a likelihood that not every cell forms a single CCV; instead, some cells may form two or more vesicles that subsequently fuse to create a TCV or CV. Live-cell super-resolution imaging (quantitatively) could be an appropriate method to either confirm or refute this possibility.

Response Reviewer #3 Point #4:

We thank the reviewer for this suggestion about presenting the quantified data, which was also made by Reviewer #1 and #2. We have added a numerical summary in Supplementary Table 1 for the FIB-SEM data. Please see Response Reviewer #1 Main Point #1 for changes made to address this point. We believe this helps clarify the other structures observed in our FIB-SEM analysis.

We agree it may be possible that two or more vesicles fuse to form the TCV. However, we do not have evidence to support this mechanism, and unfortunately the resolution of live cell super-resolution imaging is not high enough (~80nm) to discriminate a CCV and from 2 large DAVs.

5. Figure 3g depicts the co-localization of EHD1 and smoothened. Nevertheless, when observed from a longitudinal perspective, it appears that these two proteins do not exhibit strong co-localization. The authors need to verify whether this is indeed the actual situation or if it is a result of misalignment of the two channels during the data acquisition process.

Response Reviewer #3 Point #5:

We apologize for confusion caused by this image; the channels are not misaligned. We should have indicated that EHD1 and SMO-GFP are partially colocalized. We have modified the description to reflect this 'partial' localization.

Lines 250-252: "EHD1 functioning at the CCV stage is further supported by structured illumination microscopy (SIM) observations of EHD1 on CCV-like structures that partially colocalized with asymmetrically accumulated SMO-GFP (Fig. 3g)."

This partial co-localization at the MC is consistent with our previous description of EHD1 and SMO-GFP during ciliogenesis and the dynamic nature of EHD1 on the developing ciliary membrane (Lu, Insinna et al. 2015).

6. In Figure 3b, c, and d, the second bars lack annotations. To prevent confusion, the authors should label these bars as "no treatment". Also, some quantitative analyses are missing: b) no treatment vs siEHD1, no treatment vs CEP164 KO; c) serum vs no treatment, no treatment vs siEHD1; d) no treatment vs siEHD1; siEHD1 vs CCV. Same with Figure 6h.

Response Reviewer #3 Point #6:

We thank the reviewer for this suggestion. We modified the labels for the bars lacking annotations in Fig 3b, c and d.

For the quantitative analysis shown in Figure 3b-d, we compared all treatment conditions as recommended but only reported comparisons that reached statistical significance. All p values for the figures are provided in a spreadsheet with the revised manuscript, alongside the quantified data discussed. We also added notes to the figure legends to clarify these points. In Figure 6h, comparisons with the GFP control and GFP-EHD proteins were conducted to assess their effects on ciliogenesis; this clarification has been reflected in the legend shown below,

Lines 1230-1234: "Quantification of the total number of DAVs docked to individual MCs in RPE1 (GFP-CETN1 and GFP-CETN1 + SMO-tRFP) cells treated with serum (11 cells), serum-starved 3 or 6h (6 cells), lacking CEP164 serum-starved 24h (5 cells), and treated with siEHD1 (9 cells, described in e) in cells found to only have DAVs and lacking EMCs. Corresponding MC are shown in Supplementary Table 1."

7. The authors state that the EHD1 K483E mutant does not associate with tubular membranes, fails to rescue CP110 removal in EHD1-depleted cells, and shows negligible binding to GFP-CP110 compared to the wild-type protein. The above results do not support the claim in Line 321 that EHD1 membrane tubulation function is essential for interaction with CP110. There is an alternative scenario that the K483E mutation could independently impact EHD1's association with both the tubular membrane and CP110. Similarly, the results do not support the conclusion drawn in Line 334, which posits that EHD1 on tubular CCV membranes directs MC uncapping via biochemical association with CP110 and CEPE97.The authors should either provide additional evidence or rephrase these conclusions to more accurately reflect the findings of their study.

Response Reviewer #3 Point #7:
We thank the reviewer for pointing out this alternative scenario. To test this model further, we compared CP110 binding to the EHD1 W485A mutant which is unable to associate with membranes, and which also does not rescue EHD1 siRNA effects on ciliogenesis. We now show

in new Fig. 6g that this mutant fails to bind to CP110, demonstrating that EHD1 membrane association is not sufficient for CP110 binding, but rather the membrane tubulation properties of EHD1 are needed for CP110 binding. These findings are described on lines 370-373 of the revised manuscript. We have also rephrased our conclusion on the previous line 334 to reflect potential concerns the Reviewer to the following on lines 389-391: "Collectively, these results support functioning of EHD1 on tubular CCV membranes in directing MC uncapping during ciliogenesis via biochemical association with CP110 and CEP97."

References described in Reviewer rebuttal:

Halpern, A. R., G. C. M. Alas, T. J. Chozinski, A. R. Paredez and J. C. Vaughan (2017). "Hybrid Structured Illumination Expansion Microscopy Reveals Microbial Cytoskeleton Organization." ACS Nano **11**(12): 12677–12686.
Insinna, C., Q. Lu, I. Teixeira, A. Harned, E. M. Semler, J. Stauffer, V. Magidson, A. Tiwari, A. K. Kenworthy, K. Narayan and C. J. Westlake (2019). "Investigation of F-BAR domain PACSIN proteins uncovers membrane tubulation function in cilia assembly and transport." Nat Commun **10**(1): 428.
Joo, K., C. G. Kim, M. S. Lee, H. Y. Moon, S. H. Lee, M. J. Kim, H. S. Kweon, W. Y. Park, C. H. Kim, J. G. Gleeson and J. Kim (2013). "CCDC41 is required for ciliary vesicle docking to the mother centriole." Proc Natl Acad Sci U S A **110**(15): 5987–5992.
Lu, Q., C. Insinna, C. Ott, J. Stauffer, P. A. Pintado, J. Rahajeng, U. Baxa, V. Walia, A. Cuenca, Y. S. Hwang, I. O. Daar, S. Lopes, J. Lippincott-Schwartz, P. K. Jackson, S. Caplan and C. J. Westlake (2015). "Early steps in primary cilium assembly require EHD1/EHD3-dependent ciliary vesicle formation." Nat Cell Biol **17**(3): 228–240.
Zhao, Y., O. Bucur, H. Irshad, F. Chen, A. Weins, A. L. Stancu, E. Y. Oh, M. DiStasio, V. Torous, B. Glass, I. E. Stillman, S. J. Schnitt, A. H. Beck and E. S. Boyden (2017). "Nanoscale imaging of clinical specimens using pathology-optimized expansion microscopy." Nat Biotechnol **35**(8): 757–764.

We thank the reviewers for their constructive feedback and for the time invested in evaluating our revised manuscript. In response, we have revised the manuscript as suggested by Reviewer #1. We are submitting two versions of the revised manuscript: one with all changes highlighted in red and a clean version without marks.

Below, we provide a point-by-point response: reviewers' comments are in blue; our responses are in black; and quoted manuscript revisions are in red.

Reviewer #1 (Remarks to the Author):

In their revised manuscript, Lu et al. present updated findings on the process of ciliary membrane formation during ciliogenesis. They have helpfully improved their explanation and quantification of the data collected across different experiments, timepoints, and cell lines. These and other revisions have largely addressed the questions raised regarding the initially submitted manuscript. Collectively, this study showcases the power of new volumetric EM methods for high-resolution, quantitative analysis of complex and dynamic cellular processes. It is a technical tour de force and offers valuable new insights into ciliogenesis. I therefore recommend publication in Nature Communications and only highlight below a few minor points that the authors should ideally address as they finalize their manuscript.

Response: We are grateful for the reviewer's positive assessment of our work and their recognition of the potential of volumetric EM methods in studying complex cellular processes.

1. Clearly the role of EHD1 in ciliogenesis is complex and multi-factorial, given the reported effects of EHD1 knockdown on CP110 removal and on EMC formation. Regarding the section starting at line 243 "EHD1 functions in fusion of enlarged DAVs into the CCV", how compelling is the evidence that EHD1 RNAi impairs CCV formation? Figure 3 does illustrate that DAV membrane growth is unimpaired in EHD1-depleted cells (unlike in CEP164 KO cells), but it does not seem definitive that CCV formation is reduced. It is true that only two of eleven cells under this condition have CCV's at 24 h, but is this significantly different than the 8/27 wildtype RPE1 cells with CCVs (combining the WT data in Table S1)? Is the size / radial span of the 'C' shaped CCV's reduced in the EHD1 RNAi cells? While a role for EHD1 in CCV formation is certainly plausible, it may be helpful to clarify the evidence or temper the strength of the conclusions.

Response: We appreciate the reviewer's question about the evidence for EHD1 requirements in the formation of CCV structures. As suggested, we have quantified and statistically analyzed the differences in DAV and CCV structures in WT and siEHD1-treated cells. To ensure a consistent comparison across conditions, we restricted this analysis to experiments in the same reporter cell line background (RPE1 GFP-CETN1, n=16 cells for WT). Because WT cells can progress to full ciliogenesis, we restricted our analysis to DAVs and CCVs only. From this analysis, 25% of WT 3hr starved cells had DAVs (2 out of 8) and 75% exhibited CCVs (6 out 8 cells). In contrast, as noted by the reviewer, 24 h serum starved siEHD1-treated cells had 18% CCV (2 of 11 cells) and 82% DAV (9 of 11 cells). From the analysis we performed, a significant difference (p=0.024) was observed in the proportion of DAV and CCV between WT and siEHD1-knockdown cells. We also examined these ciliogenesis states in CEP164 KO RPE1 GFP-CETN1 cells that were starved for 24hr, similar to siEHD1 treated cells, and observed that all 5 cells examined had DAVs and none contained CCVs, and no statistically significant difference was observed in the proportion of these structures between siEHD1-treated and CEP164 KO cells. In the revised manuscript, we have incorporated this quantification on the following lines:

Lines 249-255: "The predominant arrest at the DAV stage suggests requirements for EHD1 upstream of CCV assembly. Consistent with this EHD1 function, RPE1 GFP-CETN1 EHD1-knockdown cells did not differ significantly in the proportion of cells with DAVs relative to CCVs compared with RPE1 GFP-CETN1 CEP164 KO cells starved for 24h (p=1). In contrast, a significant difference in the proportion of these structures (p=0.024) was observed between EHD1-knockdown cells and WT RPE1 GFP-CETN1 cells starved for 3h (Supplementary Table 1)."

Line 818: "A Fisher's exact test was used to compare DAV and CCV levels."

In addition, as suggested by the reviewer we have tempered conclusions using appropriate cautious language, such as 'largely' about EHD1 function and modified this sentence.

Lines 267-269: "Moreover, sufficient DAV membranes are present on the MC of these EHD1-knockdown cells to account for the size of an average CCV (Fig. 3d), yet these membranes largely failed to organize into the CCV."

Line 477: "The membrane shaping and fusion regulator EHD1 was found to be associated with the function in the efficient formation of tubular CCV membranes from enlarged DAVs."

We also added a potential explanation for the presence of two CCVs in EHD1-knockdown cells. This could be explained by these cells having incomplete deletion of EHD1 by siRNA

compared to cells with DAVs. We have also standardized the terminology from "EHD1-depleted" to "EHD1-knockdown" cells throughout the text for consistency. To clarify these results, we have added the following statement.

Lines 255-257: "One explanation for the presence of CCVs in a minority of EHD1-knockdown cells is that residual EHD1 protein levels remaining after siRNA treatment are sufficient to drive CCV membrane assembly."

Regarding the reviewer's question on morphology, the two CCVs identified in the EHD1-knockdown cells displayed "C" gaps (106° and 107°) dimensions comparable to those found in wild-type cells. But further statistical comparisons are limited by having only two cells with these identified structures and only one CCV was suitable for this analysis, as we only determined surface areas in CCVs lacking variable tubule extensions.

2. The presentation and interpretation of the RAB8 RNAi results remains somewhat confusing. The section title "RAB8 functions upstream of the membrane toroid structures in ciliogenesis" implies that TCV structures and downstream CV structures would be unable to form in RAB8 knockdown cells, but the quantification in Table S1 does not clearly match this prediction (e.g. CV's can form; and while no TCV was observed, only one TCV was observed across the entire study, so its absence is not necessarily compelling). I think the clearest effect is that DAV intermediates are not seen at 24 hr, indicating that RAB8 is dispensable for the DAV->CCV transition. Whether RAB8 promotes TCV formation or CV formation or CV growth into a cilium does not seem entirely clear, and thus it may be helpful to temper statements regarding the proposed role in CCV closure to form the TCV.

Response: We understand the reviewer's point and agree that the primary defect in RAB8-knockdown cells manifests after the DAV stage. Similar to our explanation to Reviewer #1 Comment #1, the observation of a CV and cilium in siRAB8-treated cells may be explained by cell to cell differences in protein ablation, and we modified the text to be more specific about these observations in the following statement.

Lines 412-413: "The observation of cells with CV or cilium likely reflects incomplete siRNA-mediated depletion of RAB8 isoforms."

We agree with the reviewer that the clearest effect of RAB8-knockdown is that DAV intermediates are not seen at 24 hr, indicating that RAB8 is dispensable for the DAV->CCV transition and have added the following statement.

Lines 413-414: "The absence of accumulated DAVs in these cells indicates that RAB8 is dispensable for the initial DAV-to-CCV transition."

Regarding the role of RAB8 in promoting TCV or CV formation or CV growth into a cilium, we have taken the reviewer's suggestion to temper the statement and have modified the subtitle and results sections given the limited TCV structures observed. These are the changes made to address this comment:

Line 400 (section title change): "RAB8 is important for ciliogenesis at the CCV stage"

Lines 421-423: "However, RAB8's function at the CCV stage appears to be unrelated to organizing membranes into tubular structures since RAB8 protein ablation does not affect this structure's assembly."

Lines 431-433: "These observations support requirements for tubular membrane assembly in instructing MC uncapping and suggest that RAB8-dependent CCV "C" gap closure upstream of TCV assembly is associated with CP110 removal."

Lines 482-483: "Specifically, our results suggest that RAB8 functions on CCVs, possibly through regulating membrane fusion."

Also, the sentence "Strikingly, our concerns about the use of TEM for precisely defining ciliogenesis mechanisms were further confirmed" (lines 401-402) is not explained.

Response: We thank the reviewer for the suggestion to clarify this sentence. We have modified this section as follows.

Lines 408-412: "FIB-SEM imaging data revealed that the majority of cells had CCVs (Fig. 7a-e, Supplementary Fig. 10a, b and Supplementary Table 1), which could potentially be misidentified as CVs by TEM depending on the sectioning plane. This confirms our concern that 2D TEM imaging can misidentify ciliogenesis intermediates."

3. I suggest some further review/editing for grammar and clarity. In the case of lines 320-321, please revise to clarify. I am also unsure what is meant on lines 385-386 regarding the effect of EHD1 over-expression: it seems that one explanation could be that in some cases ciliogenesis progression is limited independently of EHD1-faciliated removal of CP110 – is that the point that is intended here? Lastly, while a number of the reported observations are indeed compelling, the frequent use of 'strikingly' may be a bit distracting to the reader.

Response: We apologize for the grammatical errors and lack of clarity. We have carefully reviewed and edited the text for grammar and flow, including the specific revisions requested for lines 320-321.

Lines 327-330: "Consistent with this proposed MC uncapping model, CLEM FIB-SEM imaging of EHD1-knockdown RPE1 GFP-CP110 SMO-tRFP SNAP-CETN1 cells showed that cells

retaining GFP-CP110 at the MC had DAVs similar in size to those in EHD1-knockdown RPE1 GFP-CETN1 cells (Fig. 5b, Supplementary Table 1)."

Regarding the effects of EHD1 overexpression (lines 385-386), we agree with the reviewer's interpretation. It is possible that while the levels of transiently expressed EHD1 were sufficient to drive CP110 removal in some cells, they were not sufficient to drive the subsequent membrane assembly steps required for axoneme extension. We have revised the text to clarify this point as follows:

Lines 392-394: Alternatively, the levels of transiently expressed EHD1 may be insufficient to drive ciliogenesis progression in all transfected cells."

Finally, we have reduced the frequency of the word "strikingly" and "notably" throughout the manuscript to improve readability as suggested

Reviewer #2 (Remarks to the Author):

In the article "Characterization of membrane structures regulating primary ciliogenesis by quantitative isotropic ultrastructure imaging", Lu and colleagues investigate the early steps of ciliary vesicle assembly during ciliogenesis. They show for the first time that the enlargement and fusion of small vesicles at the mother centriole (MC) into novel tubular, C-shaped, and toroidal membrane structures represent a critical step in primary cilium formation. The authors further demonstrate the importance of these membrane intermediates in recruiting and stabilizing transition zone complexes, as well as in the removal of the capping complex CP110/CEP97—both processes being essential for ciliogenesis.

In this study, the authors combine several state-of-the-art imaging approaches, including volumetric FIB-SEM, structured illumination microscopy (SIM), and expansion microscopy. This innovative work sheds light on previously undescribed phenomena occurring during the early stages of ciliogenesis.

In the revised version, the authors have made a tremendous effort to address the main weaknesses of the original manuscript. I particularly appreciate the additional quantifications and immunolabeling performed for the immunofluorescence experiments, as well as the increased number of samples and replicates, despite the considerable technical challenges faced during the revision process.

Overall, I am convinced that this study will be highly insightful for the community and is fully appropriate for publication in Nature Communications.

Response: We sincerely thank the reviewer for their comments.

Reviewer #3 (Remarks to the Author):

The authors have satisfactorily addressed all my concerns. I recommend the manuscript for publication.

Response: We thank the reviewer for their comments.
